# HNF3α Targets Nckap1l and Promotes Renal Fibrosis Following Ischemia‐Reperfusion Injury

**DOI:** 10.1002/advs.202410764

**Published:** 2025-03-17

**Authors:** Ling Hou, Yan Guo, Shuang Xu, Mi Bai, Weidong Cao, Yue Zhang, Zhanjun Jia, Aihua Zhang

**Affiliations:** ^1^ Department of Nephrology Children's Hospital of Nanjing Medical University Nanjing 210008 China; ^2^ Department of Pediatrics Shengjing Hospital of China Medical University Shenyang 110004 China; ^3^ Nanjing Key Laboratory of Pediatrics Children's Hospital of Nanjing Medical University Nanjing 210008 China; ^4^ Jiangsu Key Laboratory of Early Development and Chronic Diseases Prevention in Children Nanjing Medical University Nanjing 210029 China

**Keywords:** chronic kidney disease, hepatocyte nuclear factor 3 alpha, ischemia‐reperfusion injury, NCK associated protein 1 like, renal fibrosis

## Abstract

Chronic Kidney Disease (CKD) is a global health challenge, with acute kidney injury (AKI) from ischemia‐reperfusion injury (IRI) as a common cause. This study explored the role of Hepatocyte Nuclear Factor 3 alpha (HNF3α/FOXA1) in renal fibrosis and CKD after IRI. Kidney biopsy specimens from CKD patients and mouse models (IRI or unilateral ureteral obstruction) showed HNF3α upregulation in fibrotic kidneys, linked to renal function decline. Additional experiments demonstrated that deletion of HNF3α mitigated IRI‐induced renal fibrosis, and that overexpression of HNF3α led to increased fibrosis. Examination of the potential mechanism by transcriptome sequencing and CUT&Tag sequencing suggested that HNF3α promoted renal fibrosis by increasing the expression of the NCK associated protein 1 like (Nckap1l, formerly known as hematopoietic protein 1 [Hem1]), a vital component of the WAVE complex which plays a significant role in cytoskeletal regulation and cell migration. These results underscore the critical function of HNF3α in renal fibrosis following IRI, and also identify Nckap1l as a potential therapeutic target, thus opening new avenues for research and potential therapeutic interventions for CKD and renal fibrosis.

## Graphical abstract

Hepatocyte Nuclear Factor 3 alpha (HNF3α) is significantly upregulated in renal tubular epithelial cells of CKD patients and fibrotic mice. Deletion of HNF3α mitigates IRI‐induced renal fibrosis, while overexpression worsens it. HNF3α directly regulates NCK‐associated protein 1‐like (Nckap1l), whose overexpression exacerbates fibrosis. These findings suggest that the HNF3α/Nckap1l axis is a potential therapeutic target for CKD and renal fibrosis.

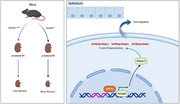

## 1 Introduction

Chronic kidney disease (CKD) is a significant global public health challenge, and has an estimated prevalence ranging from 8.2% to 9.1%.^[^ ^1^, ^2^ ^]^ CKD profoundly impacts quality‐of‐life and life expectancy in tens of millions of patients, and markedly increases the burden of cardiovascular disease and other diseases.^[^ ^1^ ^]^ In 2017, CKD was responsible for 1.2 million deaths, 41.4% more than in 1999.^[^ ^2^ ^]^ Renal ischemia‐reperfusion injury (IRI), particularly after kidney transplantation and acute kidney injury (AKI), is a major cause of CKD.^[^ ^3^, ^4^, ^5^ ^]^ IRI can occur when renal blood flow is transiently interrupted (ischemia) and then restored (reperfusion), and it sets off a cascade of cellular events that culminate in tissue damage, inflammation, and ultimately fibrotic scarring. Thus, renal fibrosis is a maladaptive repair process that is characterized by the formation and accumulation of extracellular matrix, disruption of kidney architecture and blood supply, and impaired renal function that culminates in irreversible kidney failure. This pathogenic pathway occurs in almost all patients who develop CKD.^[^ ^6^ ^]^ Hence, identification of the molecular mechanisms of renal fibrosis is of paramount importance for the development of new targeted therapies.

Hepatocyte nuclear factor 3 α, (HNF3A, also known as FOXA1), is a transcription factor in the forkhead box (Fox) family that functions in various pathological processes, and also functions in normal embryonic development, cell differentiation, cell proliferation, and apoptosis.^[^ ^7^, ^8^, ^9^, ^10^, ^11^, ^12^, ^13^ ^]^ Mice deficient in *Hnf3a* can survive postnatally, but die after 2 to 12 days due to hypoglycemia and nephrogenic diabetes insipidus.^[^ ^9^, ^13^ ^]^ HNF3α exhibits tissue‐specific expression in adult tissues, and primarily functions in the liver, pancreas, and prostate. Recent research has also reported that this protein functions in tumorigenesis and tumor progression.^[^ ^7^, ^8^, ^14^, ^15^, ^16^, ^17^ ^]^

A 2003 study that used DNA microarray analysis of kidney tissues showed that angiotensin II upregulated HNF3α in human proximal tubular cells.^[^ ^18^ ^]^ Subsequent studies demonstrated upregulation of HNF3α in sepsis‐induced AKI^[^ ^19^, ^20^ ^]^ and that HNF3α attenuated podocyte apoptosis and progression of diabetic kidney disease.^[^ ^21^, ^22^ ^]^ There is also evidence that overexpression of HNF3α mitigated ferroptosis in HK‐2 cells (normal human proximal tubular cells) that were cultured under high glucose conditions.^[^ ^23^ ^]^ In addition, a 2024 study reported that HNF3α exacerbated radiation‐induced AKI by increasing oxidative stress, cell apoptosis, and NLRP3 inflammasome activity by targeting the ITCH/TXNIP axis.^[^ ^24^ ^]^ These findings suggest that HNF3α may also function in the progression of fibrosis after IRI, although the specific mechanisms remain elusive.

The present study aimed to elucidate the effect and mechanism of HNF3α in renal fibrosis after IRI. We utilized conditional HNF3α knockout mice and intravenous injection of plasmids that overexpressed *Hnf3α* to alter HNF3α expression in mouse kidney tissues, constructed an IRI‐induced renal fibrosis model, and evaluated collagen fiber deposition, fibrosis‐related molecules, and inflammatory cytokines to determine the effect of HNF3α on renal fibrosis. We also used transcriptome sequencing with CUT&Tag sequencing to identify the potential pathways downstream of HNF3α and elucidate its mechanism.

Our general aim was to provide novel insights into the role of HNF3α in the molecular pathogenesis of renal fibrosis, and thereby lay the groundwork for new targeted therapeutic interventions that mitigate or reverse the progression of renal fibrosis and CKD.

## 2 Results

### 2.1 HNF3α is Upregulated in the Kidneys of CKD Patients and Mice with Renal Fibrosis

**Figure 1:**
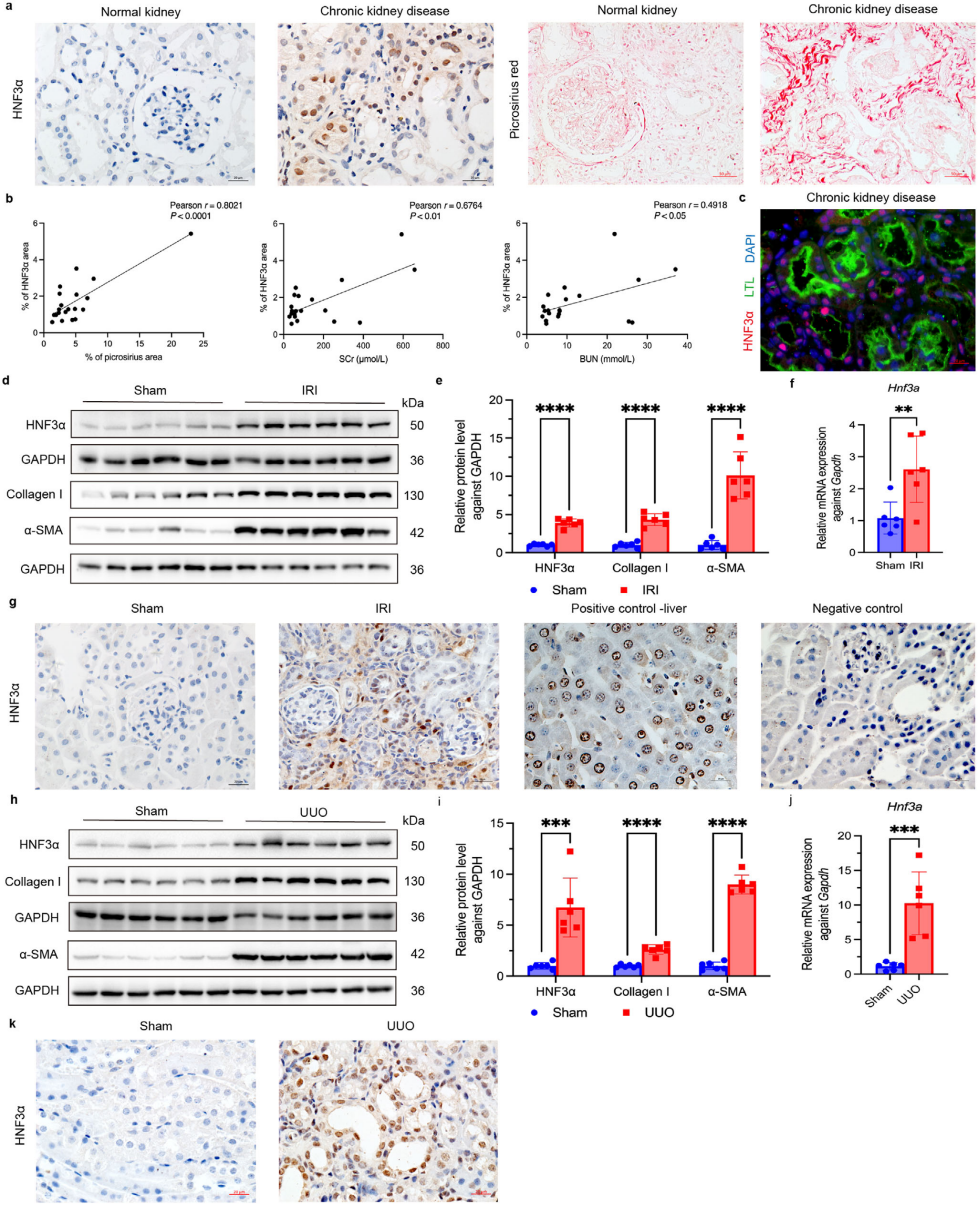
HNF3α is Upregulated in the Kidneys of CKD Patients and Mice with Renal Fibrosis. a) Biopsy samples were subjected to serial sectioning, immunohistochemical detection of HNF3α and Sirius Red staining for collagen in normal (control) kidney tissues and kidney specimens from CKD patients (*n* = 20, scale bars for HNF3α: 20 µm, scale bars for Sirius Red: 50 µm). b) Pearson correlation analysis of the relationship of HNF3α expression with collagen deposition, SCr, and BUN in CKD patients (*n* = 20). c) Immunofluorescence detection of HNF3α and *Lotus tetragonolobus* lectin (LTL) in CKD patients. d–e) Immunoblotting of HNF3α, collagen I, and α‐SMA in kidney tissues of mice at 2 weeks after IRI or sham treatment, and quantitative analysis with samples, with comparisons using a *t*‐test. f) qPCR of *Hnf3a* expression in mice that received IRI or sham treatment. g) Immunohistochemical detection of HNF3α in samples of kidney tissues from the sham, IRI, positive control, and negative control groups (scale bars: 20 µm, 3 per group). h–i) Immunoblotting of HNF3α, collagen I, and α‐SMA in mouse kidney tissues at 7 days after UUO or sham treatment, and quantitative analysis. j) qPCR of *Hnf3a* expression after UUO or sham treatment. k) Immunohistochemical detection of HNF3α in samples of kidney tissues in the sham and UUO groups (scale bars: 20 µm, 3 per group).

We obtained kidney biopsy specimens from 20 CKD patients and control specimens of normal renal tissues adjacent to tumors from 5 patients who underwent nephrectomy due to renal cancer. All patients were treated at the Department of Pediatric Nephrology and Rheumatology, Shengjing Hospital of China Medical University. We then performed immunohistochemical and immunofluorescence staining to measure HNF3α and Sirius Red staining to assess collagen fiber deposition in the renal interstitium. The results show a notable upregulation of HNF3α expression in CKD patients, predominantly in the renal tubular epithelial cells (**Figure** **1**a,c). Moreover, there were significant positive correlations of HNF3α expression with collagen fiber deposition, serum creatinine (SCr), and blood urea nitrogen level (BUN) (Figure 1b). We also analyzed microarray dataset GSE66494 from the Gene Expression Omnibus (GEO, https://www.ncbi.nlm.nih.gov/gds), which consists of kidney biopsy samples from 48 histopathologically confirmed CKD patients and 8 control samples.^[^ ^25^ ^]^ In agreement, these results also showed a marked upregulation of HNF3α in CKD patients.

To characterize HNF3α expression in fibrotic mouse kidneys, we developed two animal models of renal fibrosis: unilateral IRI and UUO. In the unilateral IRI model, immunoblotting and qPCR analyses demonstrated significant elevation of HNF3α (Figure 1d–f). In addition, the immunohistochemical findings from the IRI model aligned with those from the CKD patients, in that there was increased HNF3α expression in fibrotic kidneys, primarily in the renal tubular epithelial cells (Figure 1g). The results were also similar in the UUO mouse model (Figure 1h–j), and these mice also had increased expression of HNF3α predominantly in renal tubular epithelial cells (Figure 1k).

### 2.2 HNF3α Knockout Mitigates Renal Fibrosis in Mice with IRI

**Figure 2:**
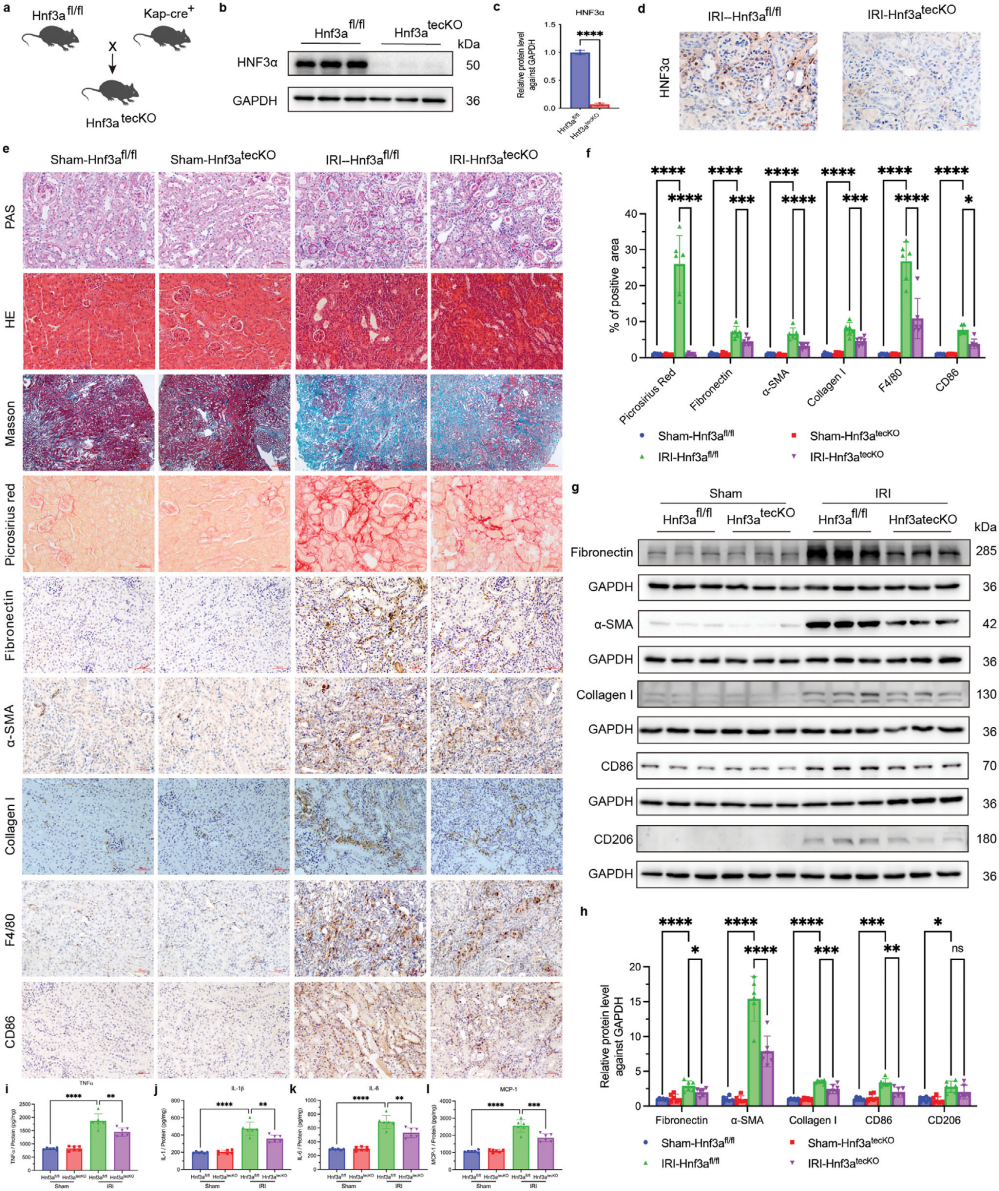
HNF3α Knockout Mitigates Renal Fibrosis in Mice with IRI. a) Floxing strategy used to breed mice with renal tubular epithelial cell‐specific knockout of *Hnf3a*. b,c) Immunoblotting of HNF3α in primary renal epithelial cells from the *Hnf*3a^fl/fl^ and *Hnf3a* ^tecCKO^ groups, and quantitative analysis (3 per group). d) Immunohistochemical imaging of HNF3α in the IRI+*Hnf3a* ^fl/fl^ and IRI+*Hnf3a* ^tecCKO^ groups (scale bars: 20 µm, 3 per group). e) Histology images at 2 weeks after renal IRI in the Sham‐*Hnf3a* ^fl/fl^, Sham‐*Hnf3a* ^tecCKO^, IRI‐*Hnf3a* ^fl/fl^ and IRI‐*Hnf3a* ^tecCKO^ groups based on PAS staining, HE staining, Masson staining, Sirius Red staining, immunohistochemistry of fibronectin, α‐SMA, collagen I, F4/80 and CD86 (scale bars for Masson staining: 200 µm, scale bars for other stains: 50 µm, 6 per group). f) Quantitative analysis of results from Sirius Red, fibronectin, α‐SMA, collagen I, F4/80 and CD86 staining. g, h). Immunoblotting of fibronectin, α‐SMA, collagen I, CD86 and CD206 in the different groups, and quantitative analysis. i–l). Renal tissue samples of mice were measured by ELISA of TNFα, IL‐1β, IL‐6, and MCP‐1 (6 per group). A one‐way ANOVA followed by Tukey's multiple comparisons test was used for comparisons.

Although the level of HNF3α was markedly elevated in the kidneys of CKD patients and mice with IRI, these results did not demonstrate a causal relationship of HNF3α with renal fibrosis. We therefore utilized CRISPR/Cas9 technology to generate *Hnf3a*‐flox mice, which were subsequently crossbred with Kap‐Cre mice to yield mice with a targeted knockout of *Hnf3a* in renal tubular epithelial cells (**Figure** **2a**). We performed genotyping of mice that were *Hnf3a* heterozygotes (±), homozygotes (+/+), wild‐type (−/−), and Kap‐cre (+) (Figure S1, Supporting Information). Next, we created the unilateral IRI model in these genetically modified mice, and collected the left kidneys after 2 weeks for evaluation. Primary proximal tubular epithelial cells isolated from the *Hnf3a* ^fl/fl^ and *Hnf3a* ^tecCKO^ mice (Figure S2, Supporting Information) showed notable decreases in the level of the HNF3α protein (Figure 2b,c). Immunohistochemistry confirmed the efficient knockout of *HNF3α*, with little or no expression in the renal tubular epithelial cells of the IRI‐*Hnf3a* ^tecCKO^ mice (Figure 2d).

Histopathological analysis (PAS, HE and MASSON staining) demonstrated that the IRI mice had renal tubular atrophy, widespread interstitial inflammation and infiltration, and collagen deposition, all hallmarks of fibrosis (Figure 2e). There was also a significantly lower level of collagen deposition in the IRI‐*Hnf3a* ^tecCKO^ mice than in the control IRI‐*Hnf3a* ^fl/fl^ mice (Figure 2e,f). Immunohistochemical analysis demonstrated markedly lower levels of the fibrosis‐associated proteins fibronectin, α‐SMA, collagen I, F4/80‐positive macrophages, and CD86‐positive M1 macrophages in the IRI‐*Hnf3a* ^tecCKO^ mice (Figure 2e,f). Similarly, immunoblotting confirmed lower expression of fibronectin, α‐SMA, collagen I, and CD86 in mice with HNF3α knockout, but no difference in the levels of CD206 (M2 macrophage marker) (Figure 2g,h). Renal tissue samples of mice were measured by ELISA of TNFα, IL‐1β, IL‐6, and MCP‐1. The results demonstrated significantly reduced levels of four inflammatory cytokines (TNFα, IL‐1β, IL‐6, and MCP‐1) in the IRI‐*Hnf3a* ^tecCKO^ mice (Figure 2i‐l). These findings indicate that the deletion of HNF3α in renal tubular epithelial cells of mice significantly mitigated renal fibrosis and the inflammatory response that was triggered by IRI. At 3 weeks post‐IRI and 7 days post‐UUO, we also found that HNF3α knockout had a protective effect (Figures S3 and S4, Supporting Information).

### 2.3 HNF3α Overexpression Aggravates Renal Fibrosis in Mice with IRI

**Figure 3:**
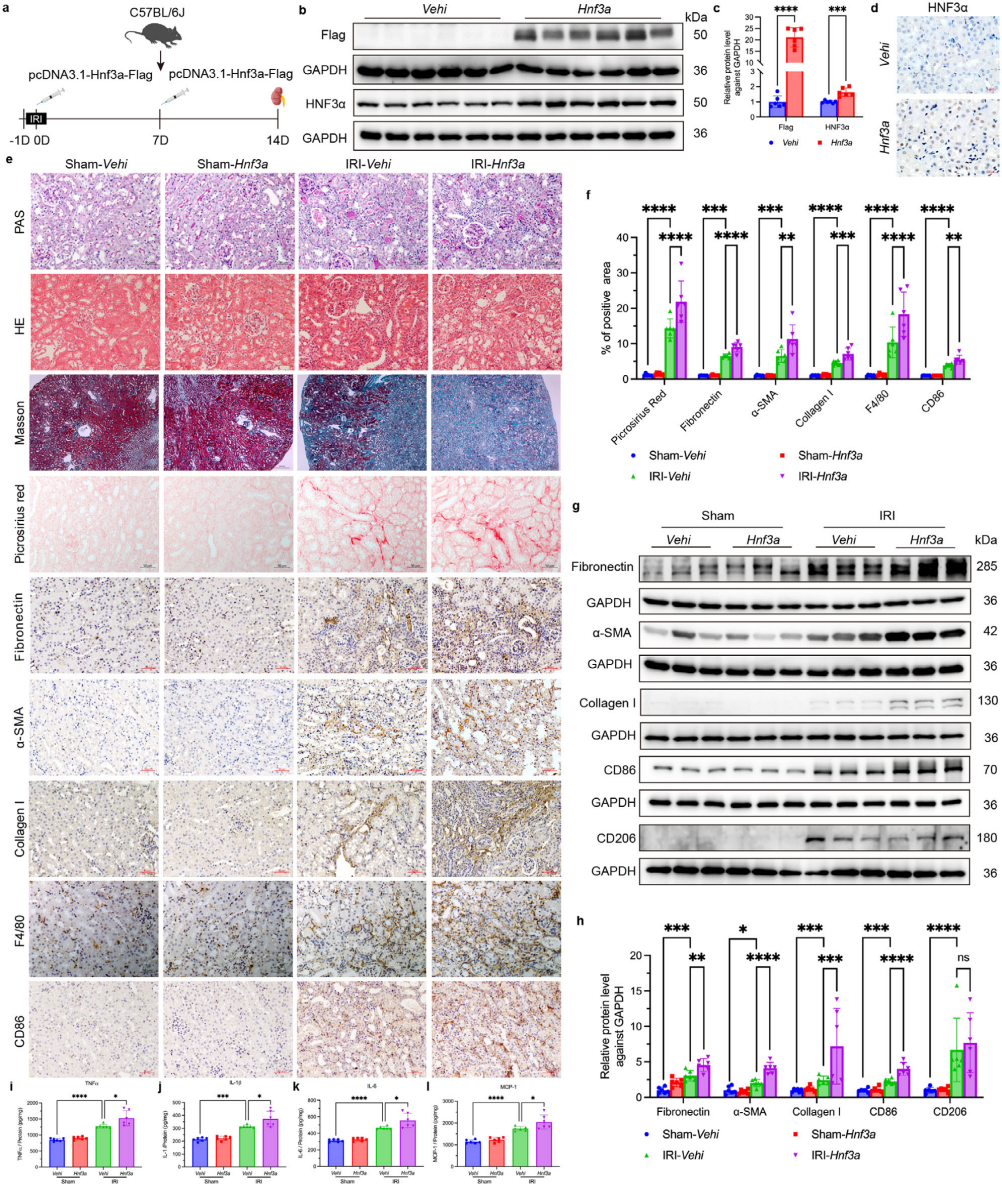
Overexpression of HNF3α Aggravates Renal Fibrosis in Mice with IRI. a) Strategy for establishment of a renal unilateral IRI model, in which mice were injected with pcDNA3.1‐*Hnf3a*‐Flag. b, c) Immunoblotting of Flag and HNF3α in the Vehi and *Hnf3a* groups, and quantitative analysis (6 per group). d) Immunohistochemical detection of HNF3α in the Vehi and *Hnf3a* groups (scale bars: 20 µm, 3 per group). e) Histology at 2 weeks after renal IRI in the Sham‐Vehi, Sham‐*Hnf3a*, IRI‐Vehi, and IRI‐*Hnf3a* groups based on PAS staining, HE staining, Masson staining, Sirius Red staining, immunohistochemistry of fibronectin, α‐SMA, collagen I, F4/80, and CD86 (scale bars for Masson staining: 200 µm, scale bars for other stains: 50 µm, 6 per group). f) Quantitative analysis of the results from Sirius Red, fibronectin, α‐SMA, collagen I, F4/80, and CD86 staining. g, h). Immunoblotting of fibronectin, α‐SMA, collagen I, CD86 and CD206 in different groups, and quantitative analysis. i–l). Renal tissue samples of mice were measured by ELISA of TNFα, IL‐1β, IL‐6, and MCP‐1 (6 per group). A one‐way ANOVA followed by Tukey's multiple comparisons test was used for comparisons.

We investigated the impact of HNF3α overexpression on renal fibrosis after IRI by administration of an HNF3α overexpression plasmid pcDNA3.1‐*Hnf3a*‐Flag to mice, and then establishing the IRI model (**Figure** **3**a). Immunohistochemical analysis confirmed the successful overexpression of HNF3α in renal tubular epithelial cells (Figure 3d). In addition, immunoblotting 3 days after injection revealed the presence of the exogenous Flag‐tag in the renal tissues, and a marked increase in the level of HNF3α (Figure 3b,c). Two weeks after IRI, the results from PAS, HE and MASSON staining demonstrated pronounced renal fibrosis (Figure 3e). Sirius Red staining showed a significant increase in deposition of collagen fiber in the IRI‐*Hnf3a* mice relative to the IRI‐Vehi mice (Figure 3e,f). Moreover, immunohistochemistry revealed significant increases in the level of fibronectin, α‐SMA, collagen I, F4/80‐positive macrophages, and CD86‐positive M1 macrophages in the IRI‐*Hnf3a* group (Figure 3e,f). This is consistent with the immunoblotting results, which showed increased levels of fibronectin, α‐SMA, collagen I, and CD86, with no difference of CD206 (Figure 3g,h). Renal tissue samples of mice were measured by ELISA of TNFα, IL‐1β, IL‐6, and MCP‐1. The results showed significant elevation of four inflammatory cytokines (TNFα, IL‐1β, IL‐6, MCP‐1) in the IRI‐*Hnf3a* mice relative to the IRI‐Vehi mice (Figure 3i–l). Taken together, these findings indicate that overexpression of HNF3α in renal tissues led to significant exacerbation of renal fibrosis and inflammation following IRI.

### 2.4 HNF3α Mediates the TGF‐β1 Induced Pro‐Fibrotic Response in Renal Tubular Epithelial Cells

**Figure 4:**
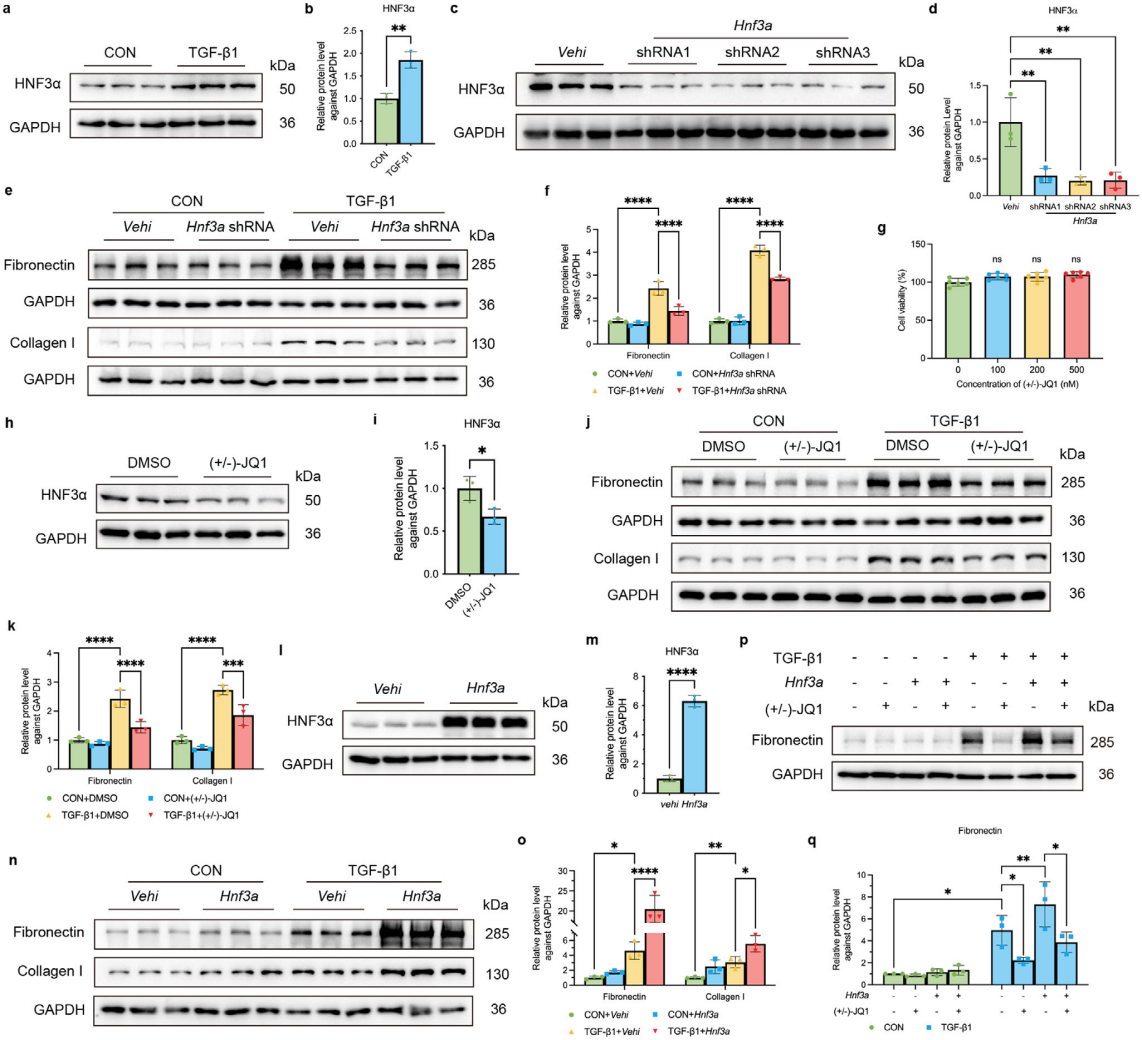
HNF3α mediates the TGF‐β1‐induced pro‐fibrotic response in renal tubular epithelial cells. a,b) Immunoblotting of HNF3α in TKPTS cells at 24 h after TGF‐β1 stimulation, and quantitative analysis (3 per group). c,d) Immunoblotting of HNF3α in different groups of TKPTS cells at 24 h after transfection, and quantitative analysis (3 per group). One‐way ANOVA followed by Tukey's multiple comparisons test was used for comparisons. e,f) Immunoblotting of fibronectin and collagen I in different groups of TKPTS cells, and quantitative analysis (3 per group). One‐way ANOVA followed by Tukey's multiple comparisons test was used for comparisons. g) Effect of (±)‐JQ1 concentration and the viability of TKPTS cells (CCK8 assay). h,i) Immunoblotting of HNF3α in TKPTS cells that were treated with 500 nM (±)‐JQ1 or DMSO (control), and quantitative analysis (3 per group). j,k) Immunoblotting of fibronectin and collagen I in different groups of TKPTS cells, and quantitative analysis. l,m) Immunoblotting of HNF3α in TKPTS cells that were transfected with an *Hnf3a* overexpression plasmid or control plasmid, and quantitative analysis (3 per group). n,o) Immunoblotting of fibronectin and collagen I in different groups of TKPTS cells, and quantitative analysis (3 per group). p,q) Immunoblotting of fibronectin in different groups of TKPTS cells, and quantitative analysis (3 per group). One‐way ANOVA followed by Tukey's multiple comparisons test was used for comparisons.

To further elucidate the mechanism of HNF3α in the TGF‐β1‐induced pro‐fibrotic processes in renal tubular epithelial cells, we conducted experiments using mouse kidney proximal tubule cells (TKPTS). Stimulating TKPTS with TGF‐β1 (10 ng mL^−1^ for 24 h) led to a significant increase in the expression of HNF3α (**Figure** **4**a,b). Thus, we transfected these cells with different *Hnf3a* shRNAs or an overexpression plasmid, or we treated them with the HNF3α inhibitor (±)‐JQ1,^[^ ^26^ ^]^ followed by TGF‐β1 stimulation (10 ng mL^−1^ for 24 h), and then measurement of fibronectin and collagen I. Immunoblotting demonstrated that 3 *Hnf3a* shRNAs were effective in decreasing the level of HNF3α (Figure 4c,d), so we used them for subsequent experiments. Notably, TGF‐β1‐stimulation of cells with *Hnf3a* shRNAs had significantly reduced levels of fibronectin and collagen I relative to the vehicle‐treated group (Figure 4e,f). Treatment of TKPTS cells with 500 nM (±)‐JQ1 (which reduces the stability of the HNF3α protein^[^ ^26^ ^]^), had no detrimental effects on cell viability (Figure 4g) and led to decreased levels of the HNF3α protein (Figure 4h,i). This treatment also led to a marked decrease in fibronectin and collagen I expression relative to cells that received TGF‐β1+DMSO (Figure 4j,k), suggesting that downregulation or inhibition of HNF3α mitigates the TGF‐β1‐induced pro‐fibrotic response. Conversely, relative to the vehicle group, TKPTS cells that overexpressed HNF3α had an increased level of fibronectin and collagen I following stimulation by TGF‐β1 (Figure 4l–o), suggesting that overexpression of HNF3α promotes the pro‐fibrotic effect of TGF‐β1 in renal tubular epithelial cells. In addition, when HNF3α was overexpressed and TGF‐β1 was added, the administration of (+)‐JQ1 inhibited the increased fibronectin expression that was induced by HNF3α overexpression (Figure 4p,q). Our examination of the human proximal tubular cell line HK‐2 led to similar results (Figure S5, Supporting Information). Examination of the rat fibroblast cell line NRK49F also demonstrated that overexpression of HNF3α increased the level of fibronectin following stimulation by TGF‐β1 (Figure S6, Supporting Information). Moreover, stimulating primary proximal tubular cells with TGF‐β1 led to a significant increase in the expression of HNF3α (Figure S7b, c, Supporting Information).

### 2.5 HNF3α Directly Promotes Transcription of Nckap1l

**Figure 5:**
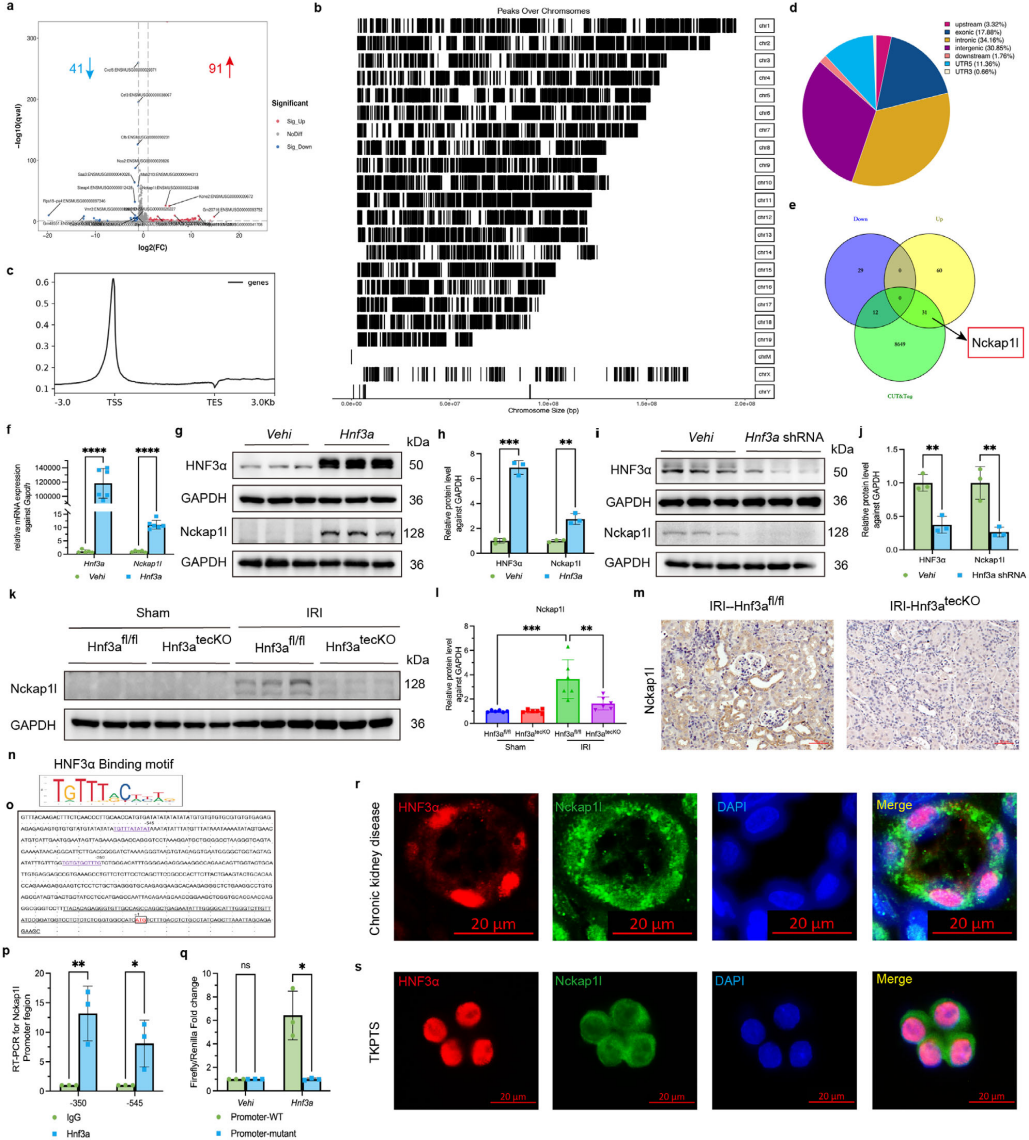
HNF3α Directly Promotes Nckap1l Transcription. a) Transcription sequencing analysis of the effect of *Hnf3α* overexpression in TKPTS cells on the number of differentially expressed genes (DEGs). b) CUT&Tag sequencing of the distributions of the *HNF3α* ChIP peaks on different chromosomes. c) Distribution of reads around the transcription start sites of different genes. d) Ratios of HNF3α‐binding peaks in different regions. e) Similarities of DEGs from RNA‐seq and the target gene set from CUT&Tag. (f) qPCR of *Hnf3a* and *Nckap1l* in the Vehi and *Hnf3α* groups (6 per group). g,h) Immunoblotting of HNF3α and Nckap1l in the Vehi and *Hnf3α* groups, and quantitative analysis (3 per group). i,j) Immunoblotting of HNF3α and Nckap1l in the Vehi and *Hnf3a* shRNA groups, and quantitative analysis (3 per group). k,l) Immunoblotting of Nckap1l in the different groups, and quantitative analysis (6 per group). A one‐way ANOVA followed by Tukey's multiple comparisons test was used for comparisons. m) Immunohistochemical imaging of Nckap1l in the IRI‐*Hnf3a* ^tecCKO^ group and the IRI‐*Hnf3a* ^fl/fl^ control group (3 per group). n) HNF3α binding motif. o) Potential binding sites of HNF3α on the *Nckap1l* promoter. p) Validation of potential binding sites by ChIP Q‐PCR (3 per group). A two‐way ANOVA followed by Šídák's multiple comparison test was used for comparisons. q) Effect of HNF3α on the *Nckap1l* promoter and mutant promoter based on the dual‐luciferase reporter assay (3 per group). A two‐way ANOVA followed by Šídák's multiple comparison test was used for comparisons. r) Immunofluorescence images of HNF3α and Nckap1l in CKD patients. s) Immunofluorescence images of HNF3α and Nckap1l in TKPTS cells.

To explore the mechanisms by which HNF3α promotes renal fibrosis, we transfected TKPTS cells with *Hnf3a* overexpression plasmids or control plasmids, and then performed transcriptome sequencing. This analysis led to identification of 41 genes with decreased expression, and 91 genes with increased expression (**Figure** **5**a) (Material S1, Supporting Information). Analysis of the genome‐wide target sites of HNF3α using CUT&Tag sequencing identified 35765 peaks, corresponding to 8692 RefSeq genes (Figure 5b–d) (Material S2, Supporting Information). Most of the HNF3α peaks were located close to transcriptional start sites (Figure 5c). Next, we analyzed the RNA‐seq with CUT&Tag data together to identify similarities among the differentially expressed genes in cells with HNF3α overexpression. These results showed there were 31 upregulated genes and 12 downregulated genes in the set of HNF3α target genes (Figure 5e). Among these genes, *Nckap1l* had significant upregulation (Figure 5e). This gene codes for the Nckap1l protein, a part of the WAVE protein complex that plays a crucial role in F‐actin cytoskeleton assembly by its interaction with the Rac signaling pathway, and affects cellular adhesion and migration.^[^ ^27^, ^28^ ^]^ Our q‐PCR analysis confirmed that *Hnf3a* overexpression in TKPTS cells led to a notable increase in the transcription of *Nckap1l* (Figure 5f), and this was confirmed by immunoblotting (Figure 5g,h). Conversely, decreased *Hnf3a* expression led to a decreased level of the Nckap1l protein (Figure 5i,j). Similarly, our analysis of the mouse model with knock out of *Hnf3a* in renal tubular epithelial cells demonstrated a significant decrease in the level of Nckap1l in the IRI‐*Hnf3a* ^tecCKO^ group relative to the IRI‐*Hnf3a* ^fl/fl^ control group (Figure 5k,l). We also performed immunohistochemical and immunofluorescence imaging of Nckap1l in the IRI‐*Hnf3a* ^tecCKO^ group and an IRI‐*Hnf3a* ^fl/fl^ control group (Figure 5m; Figure S8, Supporting Information).

We also used the bioinformatics software JASPAR (http://jaspar.genereg.net/) to identify potential HNF3α binding sites in the *Nckap1l* promoter. Thus, chromatin immunoprecipitation assays indicated there were potential binding sites of HNF3α upstream of the *Nckap1l* transcription start site, suggesting that HNF3α directly regulates the expression of this gene (Figure 5n–p). Furthermore, we constructed a *Nckap1l* promoter plasmid, and then utilized a dual‐luciferase reporter gene assay. The results show that HNF3α led to a significant increase in firefly/Renilla fluorescence, but there was no increase in firefly/Renilla fluorescence when a mutant *Nckap1l* promoter plasmid was added (Figure 5q). Immunofluorescence detection of HNF3α and Nckap1l in CKD patients and TKPTS cells showed colocalization (Figure 5r,s). These findings show that HNF3α has a pivotal role as a promoter of Nckap1l transcription within renal tubular epithelial cells, and suggest that Nckap1l contributes to the progression of renal fibrosis after IRI.

### 2.6 Elevated Nckap1l Expression in CKD Patients and Mice with Renal Fibrosis

**Figure 6:**
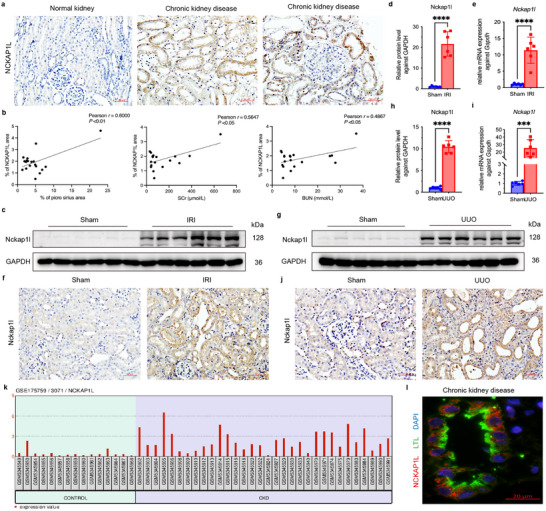
Elevated Nckap1l Expression in CKD Patients and Mice with Renal Fibrosis. a) Immunohistochemical detection of NCKAP1L in control kidney tissue and kidney biopsy specimens from CKD patients (*n* = 20, scale bars: 50 µm). b) Pearson correlation analysis of the relationship of NCKAP1L expression with collagen deposition, SCr, and BUN in CKD patients (*n* = 20). c–d) Immunoblotting of Nckap1l in kidney tissues of sham mice and IRI mice at 2 weeks after IRI, and quantitative analysis. e) qPCR of *Nckap1l* expression in the sham and IRI groups. f) Immunohistochemical detection of Nckap1l in kidney tissues of sham mice and IRI mice (scale bars: 50 µm, 6 per group). g–h) Immunoblotting of Nckap1l in kidney tissues of sham mice and UUO mice at 7 days after UUO, and quantitative analysis. i) qPCR of *Nckap1l* expression in sham and UUO mice. j) Immunohistochemical detection of Nckap1l in kidney tissues of sham and UUO mice (scale bars: 50 µm, 6 per group). k) Upregulation of *NCKAP1L* expression in the RNA‐seq dataset GSE175759 from the GEO database. l) Immunofluorescence image of Nckap1l and LTL in CKD patients.

Because HNF3α directly stimulates Nckap1l expression in renal tubular epithelial cells, we measured the levels of Nckap1l in CKD patients and in mouse models of renal fibrosis. The immunohistochemistry results showed increased expression of NCKAP1L in the kidneys (mostly within the renal tubules) of patients with CKD. This increased expression of NCKAP1L was also positively associated with the levels of collagen fiber deposition, Scr, and BUN, indicating a link to renal fibrosis (**Figure** **6**a,b). We then examined mice subjected to the unilateral IRI. The immunoblotting and q‐PCR results confirmed substantial upregulation of the Nckap1l protein and the *Nckap1l* mRNA (Figure 6c–e), and immunohistochemistry showed that Nckap1l was predominantly present in fibrotic renal tubules (Figure 6f). The results were similar for the UUO mouse model, in that these mice had significant elevations of this gene at the protein and mRNA levels, and similar immunohistochemistry results (Figure 6g–i). Further analysis of the RNA‐seq dataset GSE175759 from the GEO database, which consists of samples from 32 CKD patients and 15 controls,^[^ ^29^ ^]^ also showed significant upregulation of *NCKAP1L* in CKD patients (Figure 6k). Immunofluorescence staining of Nckap1l and LTL showed a notable upregulation of Nckap1l expression in CKD patients, predominantly in the tubular epithelial cells (Figure 6l). Thus, the upregulation of Nckap1l in human patients with CKD and in murine models of renal fibrosis points to the importance of this protein in the pathogenesis of CKD.

### 2.7 Nckap1l Overexpression Aggravates IRI‐Induced Renal Fibrosis in Mice

**Figure 7:**
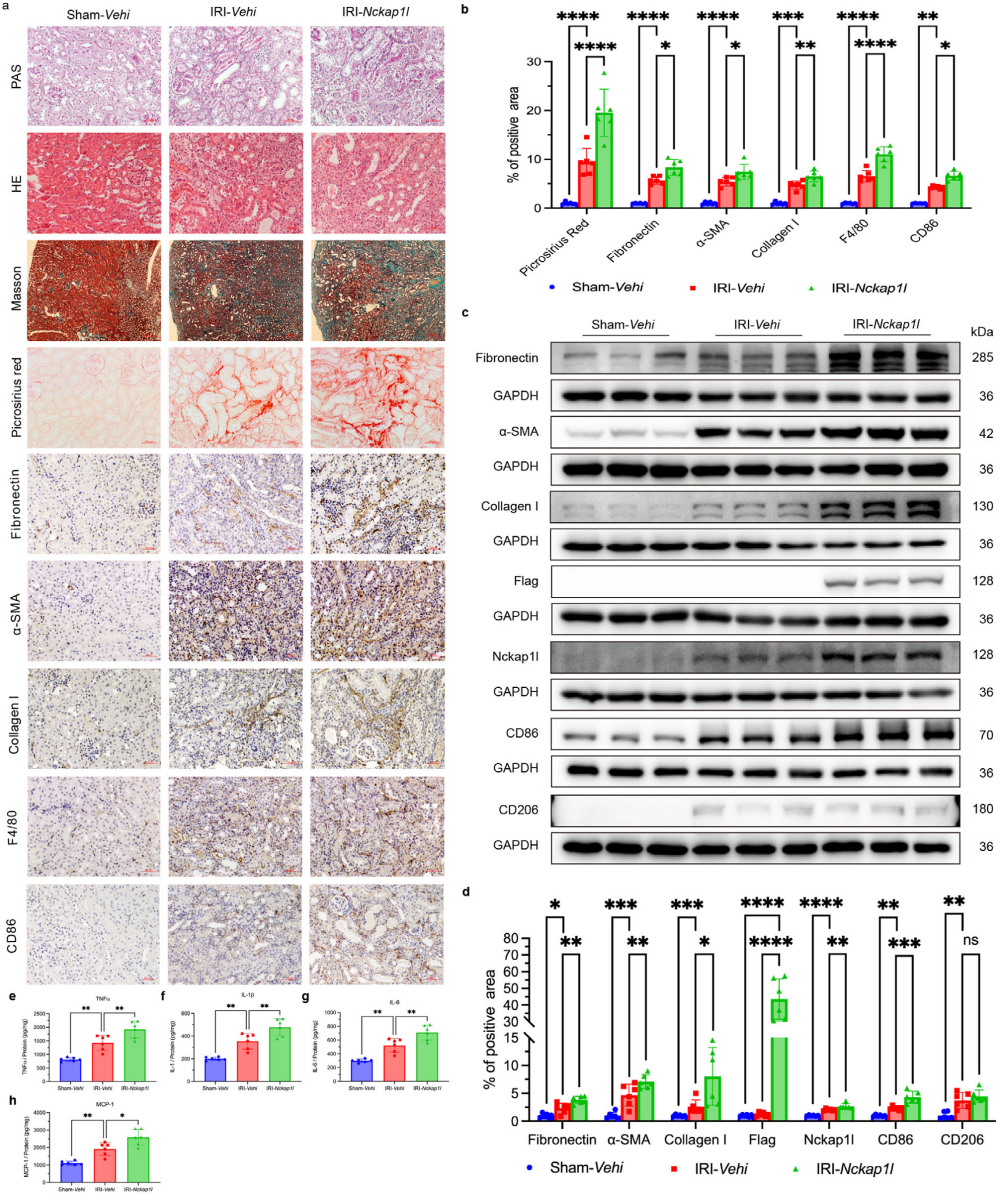
Nckap1l Overexpression Aggravates IRI‐Induced Renal Fibrosis in Mice. a) Histology at 2 weeks after renal IRI in the Sham‐Vehi, IRI‐Vehi, and IRI‐*Nckap1l* groups based on PAS staining, HE staining, Masson staining, Sirius Red staining, immunohistochemistry of fibronectin, α‐SMA, collagen I, F4/80 and CD86 (scale bars for Masson staining: 200 µm, scale bars for other stains: 50 µm). b) Quantitative analysis of the results from Sirius Red, fibronectin, α‐SMA, collagen I, F4/80 and CD86 staining. c,d) Immunoblotting of fibronectin, α‐SMA, collagen I, Flag, and Nckap1l, CD86, and CD 206 quantitative analysis. e–h). Renal tissue samples of mice were measured by ELISA of TNFα, IL‐1β, IL‐6, and MCP‐1 (6 per group). A one‐way ANOVA followed by Tukey's multiple comparisons test was used for comparisons.

Because Nckap1l levels are elevated in CKD patients and in animal models of renal fibrosis, and HNF3α regulates the transcription of Nckap1l in renal tubular epithelial cells, we examined the possible mechanism by which Nckap1l contributes to renal fibrosis. Due to the size of the *Nckap1l* gene (NM_153 505.4), which is 3405 bp, its length is suitable for insertion into lentiviral vectors but not into AAV vectors. For the same reason, it is also not suitable for overexpression plasmid. Thus, we engineered a *Nckap1l* lentiviral overexpression vector and administered it directly into the renal cortexes of mice, and then established the IRI model 7 days later. Immunoblotting revealed the presence of the exogenous Flag‐tag and a notable increase in the level of the Nckap1l protein within the kidney tissues (**Figure** **7**c,d), demonstrating successful establishment of mice with Nckap1l overexpression. Moreover, similar to our previous observations, histochemistry of kidney tissues at 2 weeks after IRI showed pronounced fibrosis based on PAS, HE and MASSON staining (Figure 7a). The Sirius Red staining showed significantly greater collagen fiber deposition in the Nckap1l‐overexpression group relative to the control (Figure 7a,b). Immunohistochemical analysis also showed that the levels of fibronectin, α‐SMA, collagen I, F4/80‐positive macrophages, and of CD86‐positive M1 macrophages were markedly increased in the Nckap1l‐overexpression group relative to the control group (Figure 7a,b). The immunoblotting results showed elevated levels of fibronectin, α‐SMA, collagen I, and CD86, but no difference in the level of CD206 in the Nckap1l‐overexpression group (Figure 7c,d). Similarly, ELISA measurements indicated increased levels of TNFα, IL‐1β, IL‐6, MCP‐1 in the Nckap1l‐overexpression group relative to the control (Figure 7e–h). These findings demonstrated that overexpression of Nckap1l in the kidneys significantly exacerbated renal fibrosis, and also led to increased infiltration of interstitial inflammatory cells and overall kidney inflammation following IRI.

### 2.8 Nckap1l Promotes the TGF‐β1 Induced Pro‐Fibrotic Response and Cell Migration in Renal Tubular Epithelial Cells

**Figure 8:**
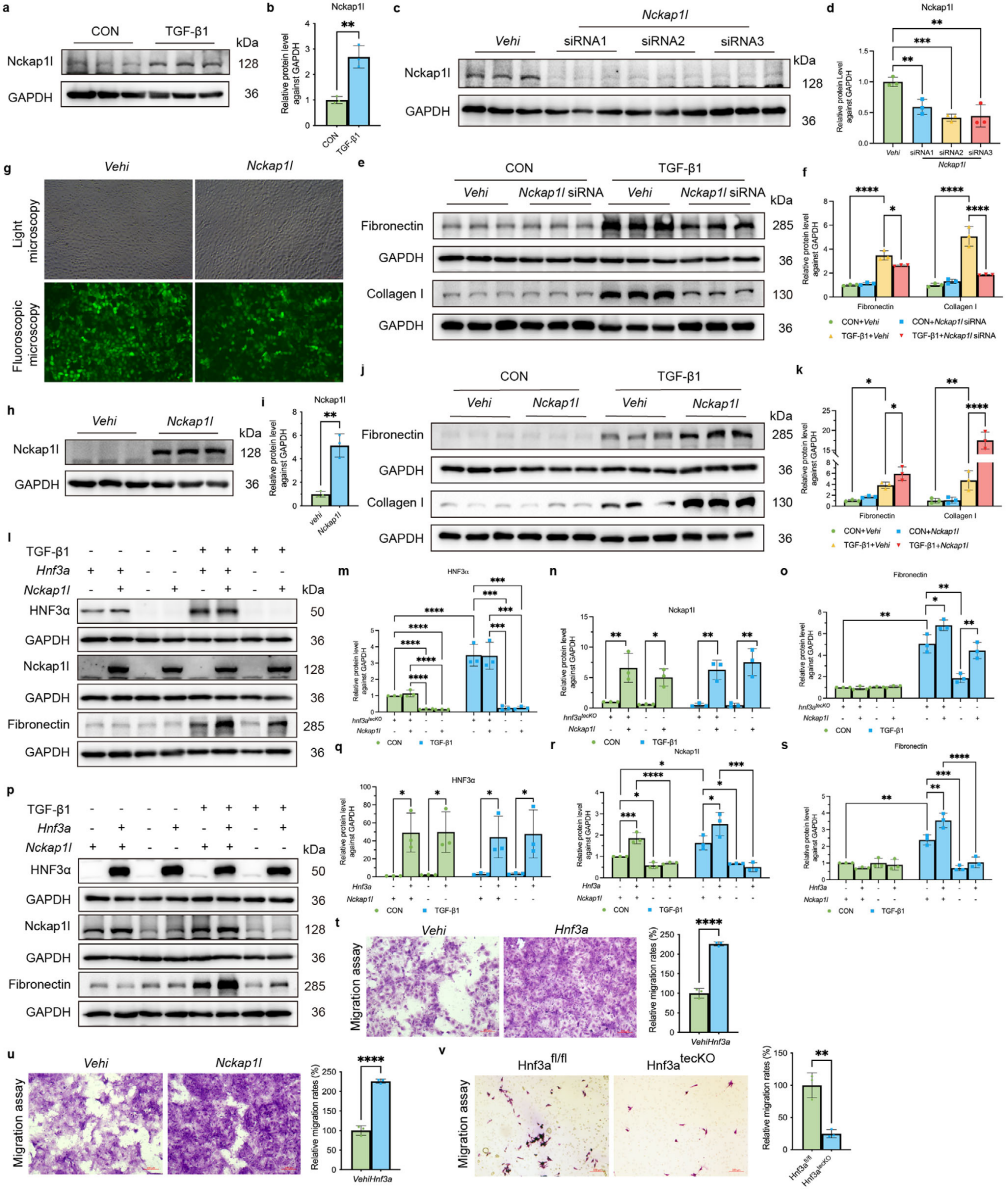
Nckap1l Promotes the TGF‐β1‐induced Pro‐fibrotic Response and Cell Migration in Renal Tubular Epithelial Cells. a,b) Immunoblotting of Nckap1l in TKPTS cells at 24 h after TGF‐β1 stimulation, and quantitative analysis (3 per group). c,d) Immunoblotting of Nckap1l in different groups of TKPTS cells at 24 h after transfection, and quantitative analysis (3 per group). One‐way ANOVA followed by Tukey's multiple comparisons test was used for comparisons. e,f) Immunoblotting of fibronectin and collagen I in different groups of TKPTS cells, and quantitative analysis (3 per group). One‐way ANOVA followed by Tukey's multiple comparisons test was used for comparisons. g) Bright field and fluorescence microscopy of TKPTS cells at 72 h after transfection with the *Nckap1l* lentiviral overexpression vector or Vehi (scale bars: 200 µm). h,i) Immunoblotting of Nckap1l in TKPTS cells that were transfected with Vehi or an *Nckap1l* overexpression vector, and quantitative analysis (3 per group). j,k) Immunoblotting of fibronectin and collagen I in different groups of TKPTS cells, and quantitative analysis. l–o) Immunoblotting of HNF3α, Nckap1l, and fibronectin in primary proximal epithelial cells from mice with (or without) conditional knockout of *Hnf3α*, with (or without) transfection of the *Nckap1l* overexpression vector, and with (or without) TGF‐β1 stimulation, and quantitative analysis (3 per group). A one‐way ANOVA followed by Tukey's multiple comparisons test was used for comparisons. p–s) Immunoblotting of HNF3α, Nckap1l, and fibronectin in TKPTS cells with (or without) transfection by an *Hnf3α* overexpression vector, with (or without) transfection of the *Nckap1l* siRNA, and with (or without) TGF‐β1 stimulation, and quantitative analysis (3 per group). A one‐way ANOVA followed by Tukey's multiple comparisons test was used for comparisons. t) Cell migration in the Vehi and *Hnf3a* groups, and quantitative analysis (scale bars: 100 µm, 3 per group). A pair‐wise *t*‐test was used for comparisons. u) Cell migration in the Vehi and *Nckap1l* groups, and quantitative analysis (scale bars: 100 µm, 3 per group). A pair‐wise *t*‐test was used for comparisons. v) Cell migration in primary proximal tubular epithelial cells from mice with (or without) conditional knockout of *Hnf3α*, and quantitative analysis (scale bars: 100 µm, 3 per group). A pair‐wise *t*‐test was used for comparisons.

Stimulating TKPTS with TGF‐β1 also led to a significant increase in the expression of Nckap1l (**Figure** **8**a,b). Thus, we transfected these cells with different *Nckap1l* siRNAs followed by TGF‐β1 stimulation, and measurement of fibronectin and collagen I. Immunoblotting demonstrated that 3 *Nckap1l* siRNAs were effective in decreasing the level of Nckap1l (Figure 8c,d), so we used them for subsequent experiments. Notably, TGF‐β1‐stimulation of cells with *Nckap1l* siRNAs significantly decreased the levels of fibronectin and collagen I relative to treatment with the vehicle (Figure 8e,f). We then utilized the *Nckap1l* lentiviral vector for overexpression studies to examine the effect of Nckap1l on the TGF‐β1‐induced pro‐fibrotic reaction and cell migration (Figure 8g–i). Immunoblotting showed a significant elevation of fibronectin and collagen I in the TGF‐β1+*Nckap1l* group relative to the TGF‐β1+Vehi group (Figure 8j,k). We also transfected primary proximal tubular epithelial cells (described above) with the same *Nckap1l* overexpression vector (Figure S9, Supporting Information). The immunoblotting results show a decreased level of fibronectin in TGF‐β1+*Hnf3a* ^tecCKO^ group compared to the controls, but an increased level of fibronectin in the TGF‐β1+*Nckap1l* group compared to controls. Intriguingly, in the absence of *Hnf3α*, overexpression of *Nckap1l* accompanied by stimulation with TGF‐β1 led to restoration of the increased expression of the fibronectin protein (Figure 8l–o). These findings suggest that *Nckap1l* overexpression augments the pro‐fibrotic response induced by TGF‐β1 in renal tubular epithelial cells. In contrast, when HNF3α was overexpressed and *Nckap1l* siRNAs and TGF‐β1 were added, the immunoblotting results showed an increased level of fibronectin in TGF‐β1+*Hnf3a* group compared to the controls, but a decreased level of fibronectin in the TGF‐β1+*Nckap1l* siRNA group compared to controls. Intriguingly, in the overexpression of HNF3α, with knockdown of Nckap1l accompanied and stimulation with TGF‐β1 led decreased the expression of the fibronectin protein (Figure 8p–s). These findings suggest that Nckap1l had an important function downstream of HNF3α.

Furthermore, the cell migration assay and cell scratch assay also showed enhanced cell migration when there was overexpression of *Hnf3a* or *Nckap1l* (Figure 8t,u; Figure S10, Supporting Information), and declined cell migration in *Hnf3a* ^tecCKO^ (Figure 8v), suggesting that HNF3α and Nckap1l actively promote the migration of renal tubular epithelial cells. We then performed experiments with staining by phalloidin, which binds specifically to F‐actin. The results showed that F‐actin polymerized and relocated to the periphery of cells in the IRI mouse model and cultured cells stimulated by TGF‐β1 (Figure S11a, b, Supporting Information). Overexpression of either *Nckap1l* or *Hnf3a* also increased the polymerization of F‐actin in TKPTS cells (Figure S12a, b, Supporting Information). Because Nckap1l promotes cell migration and this effect is linked to the increased polymerization and relocation of F‐actin, these findings point to the critical function of HNF3α in promoting the transcription of *Nckap1l*, and suggest that activation of this pathway promotes renal fibrosis.

## 3 Discussion

AKI is a formidable global public health challenge that is often caused by IRI following surgery, sepsis, and exposure to nephrotoxic substances,^[^ ^30^, ^31^ ^]^ and AKI can lead to the onset and exacerbation of CKD.^[^ ^1^, ^2^ ^]^ Examination of kidney biopsy specimens from transplant recipients showed significant alterations in transcription and cellular morphology that mirrored those in severe IRI, suggesting that even moderate renal impairment can lead to significant and long‐lasting detrimental effects.^[^ ^3^, ^32^ ^]^ Several experimental animal models that simulate renal IRI are critical tools for examining the underlying mechanisms of IRI‐induced AKI. These animal models and human renal transplants have similar alterations in immune signaling pathways, with clear increases in inflammation and fibrosis.^[^ ^33^ ^]^ Consequently, the IRI mouse model is an indispensable tool for examining the complex etiology of CKD. Despite the many insights from studying this model, there are no effective pharmacological measures that can halt the progression from AKI to CKD. This underscores the need for more studies that employ different approaches to examine the pathophysiological processes that underlie the progression from AKI to CKD.

HNF3α, a pivotal transcription factor that is expressed in the liver and other organs, regulates the normal processes of embryonic development, cell differentiation, cell migration, cell proliferation, and apoptosis.^[^ ^7^, ^8^, ^9^, ^10^, ^11^, ^12^, ^13^ ^]^ More specifically, this protein plays a foundational role in defining and sustaining the function of cells in diverse endoderm‐derived tissues, including the lungs, liver, kidneys, pancreas, and prostate.^[^ ^10^ ^]^ Oncology studies reported the absence or mutation of HNF3α in prostate cancer; its presence in estrogen receptor (ER)‐negative breast cancer is associated with a favorable prognosis; and its presence in ER‐positive breast cancer is associated with a poor prognosis.^[^ ^10^ ^]^ Thus, this transcription factor has diverse effects that depend on the organ and molecular environment.

Previous research found that HNF3α prevented the apoptosis of podocytes and helped to mitigate the progression of diabetic nephropathy,^[^ ^21^, ^22^ ^]^ and that it attenuate ferroptosis in HK‐2 cells under high‐glucose conditions.^[^ ^23^ ^]^ Furthermore, the expression of this protein is greater in sepsis‐induced AKI,^[^ ^19^, ^20^ ^]^ and it can also exacerbate the severity of radiation‐induced AKI.^[^ ^24^ ^]^ However, the effect and mechanism of HNF3α on the progression from AKI to CKD remain underexplored.

Our analyses of biopsy specimens and re‐evaluation of microarray data from the GEO database (GSE66494) identified a marked increase in the expression of HNF3α in the renal tubular epithelial cells of CKD patients. In addition, our examination of two mouse models of renal (IRI and UUO) showed there was consistent upregulation of HNF3α. We also created mice that had conditional knock‐out of *Hnf3α* and mice that had increased expression of *Hnf3a* following introduction of an overexpression plasmid. In agreement with our observations of CKD patients, these experimental manipulations of Hnf3α expression demonstrated that the level of this protein affected the progression of kidney fibrosis and inflammation after IRI. These insights indicate that HNF3α has a critical regulatory function after renal IRI, and suggest it has promise as a target for therapeutic interventions that aim to treat the renal fibrosis that occurs during CKD.

Our examination of renal tubular epithelial cells showed that knockdown of *Hnf3a* and inhibition of HNF3α protein stability led to decreased TGF‐β1‐induced expression of fibronectin (a component of the extracellular matrix), and that overexpression of *Hnf3a* had the opposite effect. These findings also highlights the pivotal role of HNF3α in mediating the pro‐fibrotic response triggered by TGF‐β1. Our integration of findings from transcriptome and CUT&Tag sequencing suggested that HNF3α directly regulates the transcription of *Nckap1l*, and we validated this interpretation using immunoblotting, quantitative PCR, luciferase reporter assays, and chromatin immunoprecipitation. These findings indicate that the effect of HNF3α on renal fibrosis and CKD may be attributable to its regulation of *Nckap1l* and its downstream signaling pathways.

Nckap1l regulates the actin cytoskeleton in hematopoietic cells and is a key protein of the WAVE complex. The WAVE complex relays signals to the activated Rac, promotes the polymerization of F‐actin, and has roles in vital processes such as cell adhesion and migration.^[^ ^27^, ^28^, ^33^, ^34^ ^]^ Recent findings have underscored the vital connection of Nckap1l with immune cell function^[^ ^28^, ^33^, ^35^, ^36^, ^37^, ^38^ ^]^ and the development of intrahepatic bile duct networks.^[^ ^39^ ^]^ Our immunohistochemistry analyses revealed a marked increase in NCKAP1L expression in kidney biopsy samples of individuals with CKD, mainly in renal tubular epithelial cells. Additionally, our RNA‐seq analysis of the GSE175759 dataset from the GEO database showed significantly greater expression of *NCKAP1L* in patients with CKD. Our experiments that examined mouse models of IRI and UUO found a significant elevation of Nckap1l expression in mice that had fibrosis relative to control mice. Further experiments that utilized lentiviral vectors to induce localized overexpression of *Nckap1l* in mouse kidneys demonstrated that this overexpression intensified the fibrosis and inflammation after induction of IRI. Altogether, these findings suggest that Nckap1l is a key factor that affects renal fibrosis after IRI, and that the effect of HNF3α is likely mediated through Nckap1l.

We also examined the effect of inducing overexpression of *Nckap1l* in cultured renal tubular epithelial cells. Similar to the effect of *Hnf3α* overexpression, this intervention also increased the TGF‐β1‐induced expression of fibronectin. Interestingly, *Nckap1l* overexpression in the absence of *Hnf3a* reinstated the increased level of fibronectin. These findings confirm that overexpression of Nckap1l enhances the TGF‐β1‐induced pro‐fibrotic response in renal tubular epithelial cells. Consequently, it appears that the critical regulatory effect of HNF3α on renal fibrosis after IRI is mediated by its promotion of Nckap1l expression, although the precise mechanism warrants further exploration. Because HNF3α and Nckap1l both facilitate cell migration—a crucial feature of the progression of renal fibrosis that involves transformation of renal tubular epithelial cells and the active engagement of fibroblasts, renal interstitial cells, and immune cells^[^ ^40^, ^41^ ^]^—we explored their impact on cell migration in vitro. The results showed that overexpression of *Hnf3α* or *Nckap1l* greatly increased the migratory capacity of renal tubular epithelial cells.

What could account for this increased migration of renal tubular epithelial cells? It is known that Nckap1l affects the dynamics of F‐actin, an integral part of the WAVE complex which plays a pivotal role in actin filament polymerization and depolymerization, thus impacting cell morphology, migration, and extracellular interactions.^[^ ^27^, ^28^, ^33^, ^34^ ^]^ We therefore hypothesize that HNF3α and Nckap1l promote cell migration by altering the intracellular polymerization and location of F‐actin. Consistent with this hypothesis, our examination of changes in the distribution of F‐actin in renal tubular epithelial cells after IRI in vivo and after TGF‐β1 treatment in vitro showed reorganization and aggregation of F‐actin at the periphery of cells. We also observed these F‐actin aggregations in cells that were treated with TGF‐β1 and, to a lesser extent, in cells that overexpressed *Hnf3α* or *Nckap1l*. Thus, HNF3α appears to play a pivotal regulatory role in renal fibrosis after IRI, potentially by controlling the transcription of *Nckap1l*, increasing cell migration, and promoting the aggregation of F‐actin. Thus, the targeting HNF3α and Nckap1l appears to have potential as novel therapeutic avenue for treatment of renal fibrosis.

Studies of breast cancer found that HNF3α inhibited the nuclear translocation of Smad3 (a key transcription factor downstream of the TGF‐β signaling pathway) by suppressing its binding with the nuclear import receptor importin‐7. Moreover, knockdown of HNF3α upregulated the expression of pro‐apoptotic genes mediated by Smad3. These results suggest that HNF3α expression is negatively associated with survival in patients with breast cancer.^[^ ^42^ ^]^ In renal tubular epithelial cells, we found that TGF‐β1 can induce an increase in HNF3α expression, and HNF3α mediated the TGF‐β1‐induced pro‐fibrotic response. However, the exact mechanism of this effect and whether it occurs through the typical TGF‐β/Smad signaling pathway remains unclear, warranting further investigation.

This study had certain limitations, notably an extensive reliance on animal models of renal fibrosis after IRI. Future research in this area should expand our understanding of the role of HNF3α in renal fibrosis by examination of other animal models, such as the 5/6 nephrectomy model. In addition, examination of other transgenic animals with altered levels of *Hnf3a* and *Nckap1l* will be an important avenue for further research. Furthermore, examination of the molecular mechanism of HNF3α could be enhanced by identification of its other downstream targets, by a more comprehensive examination of the role of Nckap1l in fibrosis, and exploration of the effects of synergistic therapeutic approaches.

After kidney injury, the damaged tubular epithelial cells undergo a series of changes. On the one hand, they undergo epithelial‐mesenchymal transition (EMT), and significantly upregulating a variety of pro‐fibrotic cytokines. On the other hand, they adopt a secretory phenotype and subsequently release a series of pro‐inflammatory mediators. Additionally, they becomes arrested at the G1/S and G2/M checkpoints. The increased number of G2/M‐arrested tubular epithelial cells produce TGF‐β and CTGF, thereby promoting renal fibrosis. Thus, injured tubular epithelial cells exacerbate kidney inflammation and fibrosis through these mechanisms. Whether HNF3α promotes renal fibrosis through the aforementioned mechanisms requires further investigation in the future studies.

**Figure 9:**
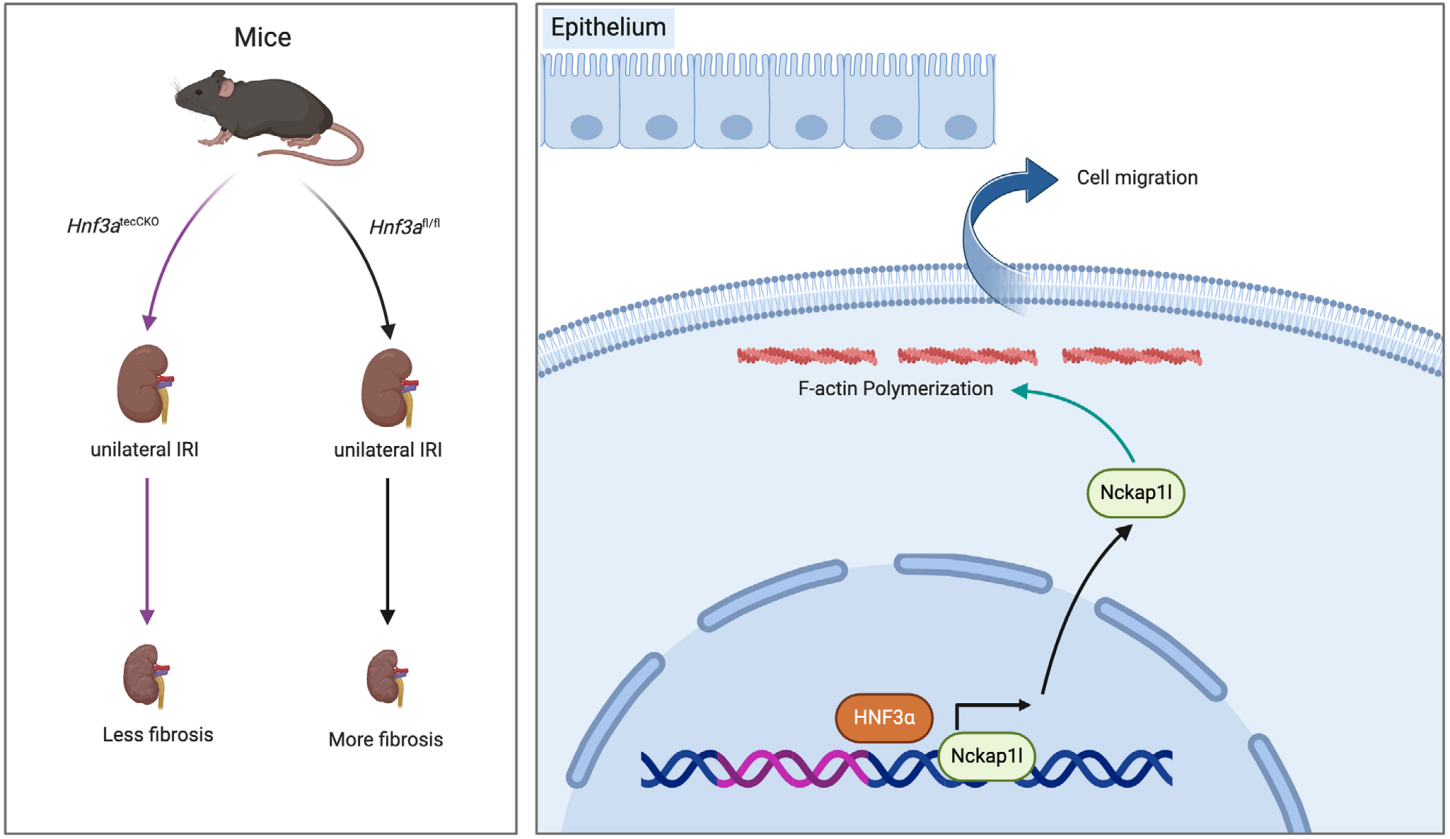
Proposed mechanism by which HNF3α promotes the progression of renal fibrosis due to its direct regulation of Nckap1l transcription, thereby disrupting cell migration. (Created in BioRender. https://BioRender.com/k34y766).

To summarize, our findings suggest that HNF3α could promote the progression of renal fibrosis by directly regulating the transcription of *Nckap1l*, thereby altering cell migration (**Figure** **9**).

## 4 Experimental Section

### Patient Enrollment

This study used renal biopsy samples from 20 patients diagnosed with CKD and control samples from normal renal tissues that were adjacent to tumors in 5 patients with renal carcinoma who received nephrectomies. All patients were from the Department of Pediatric Nephrology and Rheumatology, Shengjing Hospital of China Medical University (Table S1, Supporting Information). Paraffin‐embedded kidney biopsy sections were analyzed. The China Medical University Ethics Committee approved this research (2024PS1079K), which complied with the ethical standards delineated in the 1964 Helsinki Declaration and its later amendments or comparable ethical norms. Informed consent was secured from all participating patients and their parents. Biopsy samples were subjected to serial sectioning, followed by immunohistochemical and Sirius red staining, and correlation analysis.

### Construction of Hnf3a^tecCKO^ Mice

*Hnf3a*‐flox mice (strain No. T017857), which have a C57BL/6J genetic background, were from GemPharmatech (Nanjing, China). CRISPR/Cas9 technology was used to target exon 2 of Hnf3a‐201 (ENSMUST00000044380.7) for knockout. To achieve conditional knockout of *Hnf3a* in renal tubular epithelial cells, Hnf3a‐flox mice were crossbred with Kap‐Cre mice from the Jackson Laboratory. The primers used for genotyping are listed in Table S2 (Supporting Information). Eight‐week‐old male mice were selected to create a renal unilateral IRI model, and four groups were established, with 6 mice per group: Sham+*Hnf3a* ^fl/fl^, Sham+*Hnf3a* ^tecCKO^, IRI+*Hnf3a* ^fl/fl^, and IRI+*Hnf*3a^tecCKO^. The procedure for establishing the unilateral IRI model is described below.

### Animal Models

C57BL/6J male mice (age: 8–10 weeks) were from Vital River Laboratory Animal Technology (Nanjing, China) and acclimated in a specific pathogen‐free (SPF) facility for 1 week prior to experiments. The renal unilateral IRI model was established as detailed previously.^[^ ^43^ ^]^ Briefly, after administering isoflurane anesthesia, the abdomen was exposed and the left renal pedicle was clamped with a non‐invasive microartery clip (No. 18050‐28; Fine Science Tools, Heidelberg, Germany) at 37 °C for 30 min. Sham mice were subjected to abdominal exposure without pedicle clamping. The *Hnf3a* overexpression plasmid (pcDNA3.1‐*Hnf3a*‐Flag) and control plasmid (pcDNA3.1‐Flag) were from the Public Protein/Plasmid Library (Nanjing, China). These plasmids were dissolved in 2 mL of normal saline (70 µg per mouse), and then injected into the tail vein under high pressure within 5 to 7 s,^[^ ^44^, ^45^ ^]^ leading to establishment of unilateral IRI after 24 h. These experiments divided mice into four groups, with 6 mice per group: Sham+Vehi, Sham+*Hnf3a*, IRI+Vehi, and IRI+*Hnf3a*. To achieve overexpression of Nckap1l, the lentiviral vector pcSLenti‐EF1‐EGFP‐CMV‐*Nckap1l*‐MCS‐3xFLAG‐WPRE and a corresponding control were obtained from OBiO Technology (Shanghai, China). This vector targets *Nckap1l* (NCBI reference sequence: NM_153 505.4, gene ID: 105 855). These mice were divided into three groups, with 6 mice per group: Sham+Vehi, IRI+Vehi, and IRI+*Nckap1l*. These mice received injections into the intrarenal cortex of the vector or control solution (10 × 10^6^ Tu per mouse, total volume: 20 µL, 4 injection sites each at the upper and lower poles), and were subjected to unilateral IRI procedures 1 week later. All animals were euthanized two weeks after model construction for analysis of kidney tissues.

The UUO model was constructed using a similar procedure. In particular, 8‐week‐old male mice received anesthesia, and then surgical left ureteral obstruction or a sham operation.^[^ ^43^ ^]^ These mice were also euthanized after 7 days for analysis of kidney tissues. All animal protocols followed ethical guidelines and were approved by the Institutional Animal Care and Use Committee of Nanjing Medical University (IACUC‐2204050).

### Cell Culture and Treatments

Mouse kidney proximal tubule cells (TKPTS) were from ATCC (CRL‐3361, Manassas, VA) and were cultured in DMEM: F12 (Gibco, Carlsbad, CA) that was supplemented with 7% FBS (Gibco) and 6 µg mL^−1^ insulin (HY‐P0035/CS‐7905, MCE). These cells were maintained at 37 °C in a 5% CO_2_ atmosphere, and were passaged twice per day at a 1:7 ratio. Human renal tubular epithelial cells (HK‐2) (FuHeng Biology Cell Bank, Shanghai, China, cat: FH0228) were cultured in DMEM: F12 with 10% FBS, while rat kidney fibroblasts (NRK‐49F) (ATCC, cat: CRL‐1570) were cultured in DMEM with 10% FBS. Both cell types were passaged twice per day at a 1:2 ratio. These cells were then transfected with the pcDNA3.1‐*Hnf3a*‐Flag plasmid or the *Hnf3a* shRNA plasmid or the *Nckap1l* siRNAs from the Public Protein/Plasmid Library and Haixing Bioscience. The specific shRNA sequences are listed in Tables S3 and S4 (Supporting Information). In brief, cells were plated in 6‐well plates, and when they reached 60 to 70% confluence, they were transfected using jetPRIME (114‐15, polyplus, Strasbourg, France) for 4 to 6 h. Then, the cells were switched to serum‐free medium, 10 ng mL^−1^ TGF‐β1 (100‐21, PEPROTECH, USA) was added, and the cells were harvested 24 h later. For experiments using the pcSLenti‐EF1‐EGFP‐CMV‐*Nckap1l*‐MCS‐3xFLAG‐WPRE vector, cells were placed in 24‐well plates, and when they reached ≈40% confluence they were transfected with 5 µL of polyprene plus and 5 µL of the vector (or control) in 500 µL of culture medium. The next day, this medium was replaced with fresh culture medium, and the level of GFP (transfection efficiency) was determined by fluorescence microscopy (TH4‐200, Olympus, Japan) 48 to 72 h later. Cells with transfection efficiency above 80% were further cultured, and then exposed to serum‐free medium supplemented with 10 ng mL^−1^ TGF‐β1 for 24 h. The HNF3α inhibitor (±)‐JQ1 (SML0974, Sigma‐Aldrich, US) was added to the serum‐free medium 4 h before TGF‐β1 stimulation.

### Isolation of Primary Proximal Epithelial Cells

*Hnf3a* ^fl/fl^ and *Hnf3a* ^tecCKO^ mice that were 4 to 6‐weeks‐old were euthanized, disinfected in alcohol for 5 min, and the abdomens were then opened to extract the kidneys. The outer renal capsule was removed, and the renal cortex was passed through an 80‐mesh sieve and then cut into 1 to 2 mm^3^ pieces. These pieces were pulverized into a paste using the core of a glass syringe while rinsing with cold PBS until the paste was white. The resulting slurry was passed through a 100‐mesh sieve, and the retained material was centrifuged at 5000 rpm for 10 min. The supernatant was discarded, and the pellet was treated with type I collagenase, incubated at 37 °C for 30 min, centrifuged again, and the resulting pellet was collected again. This pellet was then resuspended in DMEM: F12 medium that had 10% FBS and 1% penicillin‐streptomycin (Gibco), and the cells were cultured with 5% CO_2_ at 37 °C for 7 days to promote cell growth. The procedures used for transfection with the *Nckap1l* overexpression vector were the same as previously described.

### Renal Pathology Examination

Kidney samples were fixed in 4% formaldehyde for 24 h, dehydrated, embedded in paraffin, and then sectioned into 3‐µm slices. Periodic acid‐Schiff (PAS) staining was used to visualize morphology. Slices underwent deparaffinization, rehydration, and oxidation with 1% periodic acid for 10 min, followed by staining with the Schiff reagent (Sigma‐Aldrich, St. Louis, MO) at 42 °C for 40 min. After washing and counterstaining with hematoxylin, slices were dehydrated, cleared, and mounted. Masson's trichrome staining was used to discriminate connective tissues (fibrosis) and cells. Thus, 5‐µm slices underwent deparaffinization and rehydration, treatment with Bouin's solution at 56 °C for 1 h, counterstaining with hematoxylin for 15 min, staining with Masson's red for 30 s, and bright green for 1 min. This was followed by washing, dehydrating, and mounting. Sirius Red staining was used to visualize collagen (fibrosis). Thus, 5‐µm slices were subjected to deparaffinization and rehydration, staining with Sirius Red solution (Maokang Biotechnology, Shanghai, China) at room temperature for 1 h, and then rinsing, dehydration, and mounting. All of these images were recorded with a Zeiss AX10 microscope (Carl Zeiss, Jena, Germany), and the stained areas were quantified using ImageJ software (National Institutes of Health, Bethesda, MD).

### Immunohistochemical and Immunofluorescence Analysis

Immunohistochemistry (IHC) was used to analyze human and mouse kidney sections. Initially, sections underwent deparaffinization and rehydration, and then antigen retrieval was performed in a sodium citrate solution (P0083; Beyotime, Shanghai, China) with microwave treatment for 15 min. To neutralize endogenous peroxidase activity, 3% hydrogen peroxide (Zhong Shan‐Golden Bridge, Beijing, China) was added at room temperature for 20 min. Sections were then blocked using an immunostaining blocking solution (P0102; Beyotime) for 1 h. Primary antibodies were applied and incubated overnight at 4 °C using specific dilution ratios (Table S5, Supporting Information). The immunofluorescence (IF) procedure was the same as above, except for the 3% hydrogen peroxide treatment step. For IHC, this process was followed by incubation at room temperature for 20 min with an enhancing solution and addition of an appropriate secondary antibody (pv‐9000; Zhong Shan‐Golden Bridge). Then, sections were stained with DAB (ZLI‐9017; Zhong Shan‐Golden Bridge). A brown color was considered positive. For IF, this process was followed by incubation at room temperature for 1 h with an appropriate secondary antibody. For the quantitative analysis, 10 fields of view at 200× were randomly selected, and staining was quantified as described above.

### Western Blot Analysis

Tissues or cells were added to a lysis buffer (P0013K; Beyotime) that was supplemented with a Complete Protease Inhibitor Cocktail (11 697 498 001, Roche, Basel, Switzerland). The lysates were then homogenized and cooled on ice for 30 min, followed by centrifugation (5000 rpm, 4 °C, 30 min). The protein concentration in the supernatant was quantified using the Pierce BCA Protein Assay (ThermoFisher Scientific, Waltham, MA). For electrophoresis, 15 to 20 µg of protein was added to each lane, proteins were separated by SDS‐PAGE (Epizyme, Shanghai, China), and the proteins were transferred to PVDF membranes (162‐0177; Bio‐Rad, Hercules, CA). These membranes were blocked using 5% milk and incubated with primary antibodies overnight at 4 °C, followed by incubation with an appropriate secondary antibody at room temperature for 1 h. The dilution ratios for antibodies are listed in Table S5 (Supporting Information). Protein detection was carried out using an ECL chemiluminescence kit (185 001; Tanon, Shanghai, China), and images were acquired using the Tanon Chemiluminescence Image Analysis System. ImageJ software was used for quantitative analysis of the protein bands.

### Quantitative PCR

RNA extraction was performed utilizing RNAiso Plus (TaKaRa, Shiga, Japan), and then 1 µg of the total RNA was reverse‐transcribed into cDNA using the HiScript II 1st Strand cDNA Synthesis Kit (R211‐01; Vazyme, Nanjing, China). PCR amplification was performed using the AceQ qPCR SYBR Green Master Mix (Q131‐02; Vazyme) on a LightCycler 96 Real‐Time PCR System (Roche). All primer sequences were from the PrimerBank website (https://pga.mgh.harvard.edu/primerbank/index.html) and shown in Table S2 (Supporting Information). Quantitation was performed using the 2^−ΔΔCt^ method, Glyceraldehyde‐3‐phosphate dehydrogenase (*Gapdh*) was utilized as an internal control.

### ELISA

TNFα, IL‐1β, IL‐6, and MCP‐1 were quantified utilizing specific ELISA kits (JL10484, JL20241, JL20268, JL20304, Jianglai, Shanghai, China) according to the manufacturer's instructions. Renal tissue samples were first homogenized in cold PBS (1:9 ratio) and centrifuged, and the resultant supernatant was diluted. The ELISA plates were equilibrated at 37 °C for 10 min, and then 100 µL of each diluted sample or standard was added into the wells, followed by incubation at 37 °C for 1 h. Then 100 µL of the biotinylated antibody solution was added at 37 °C, and the solution was incubated for an additional 1 h. After washing the plates, 100 µL of the enzyme conjugate solution was added to each well, followed by incubation at 37 °C for 30 min. The solution was then washed, 90 µL of the TMB substrate was added to each well, followed by incubation at 37 °C in the dark for 15 min. The reaction was halted by adding 50 µL of the stop solution, and the optical density was then measured at 450 nm (OD_450_). OD_450_ values were adjusted relative to the uncoated (control) well, and sample concentration was calculated by fitting a four‐parameter logistic curve of corrected OD_450_ values on a double‐logarithmic scale using Origin version 8.0 software (OriginLab, China). Protein concentrations (normalized results) were analyzed with ImageJ software.

### CCK‐8 Assay

An aliquot of the cell suspension was added into a 96‐well plate, incubated for 24 h, and then different concentrations of (±)‐JQ1 were added. After 48 h, the medium was refreshed, and 5 µL of CCK‐8 solution (C0042, Beyotime) was added into each well. The plate was then incubated in darkness in a culture incubator for 1 to 4 h. OD_450_ was measured every hour with a microplate reader to determine cell viability.

### ChIP Q‐PCR

The Chromatin Immunoprecipitation (ChIP) assay was carried out in accordance with the protocol of the SimpleChIP Enzymatic Chromatin IP Kit (9003, Cell Signaling Technology, USA). TKPTS cells that were transfected with pcDNA3.1‐*Hnf3a*‐Flag for 24 h were fixed with formaldehyde, neutralized with glycine, and then lysed using enzymatic methods. DNA was fragmented by sonication, immunoprecipitated overnight at 4 °C using an HNF3α monoclonal antibody, with details described in Table S5 (Supporting Information). Then, magnetic beads were added to the samples, followed by incubation for 2 h at 4 °C. The immune complexes were then washed with low‐ and high‐salt buffers, the crosslinks were reversed, and DNA was isolated using spin columns. The qRT‐PCR analyses utilized primers that targeted the promoter region of *Nckap1l*, with details provided in Table S2 (Supporting Information).

### Transwell Migration Assay

TKPTS cells were processed to prepare single‐cell suspensions, concentrated to 5 × 10^4^ cells in 100 µL of serum‐free medium, added into a Transwell chamber (Labselect, China), and then subjected to a 24‐h transfection with pcDNA3.1‐*Hnf3a*‐Flag. Similarly, cells transfected with the *Nckap1l* overexpression vector or primary proximal epithelial cells were processed, and 1 × 10^5^ cells were suspended in 100 µL of serum‐free medium, and then transferred into the transwell chamber. The lower chamber was filled with DMEM: F12 medium that was enriched with 20% FBS. These cells were incubated for 24 h, and the chambers were then subjected to the staining and counting protocol. Excess cells on the upper surface were carefully removed using a cotton swab, and the remaining cells were fixed with 4% formaldehyde for 15 min, and then stained with 0.1% crystal violet for 10 min. Microscopic imaging (400×) of five randomly selected fields was used for cell counting, and the results of the different groups were compared.

### Cell Scratch Assay

One group of cells was cultured in 6‐well plates and transfected with pcDNA3.1‐*Hnf3a*‐Flag for 24 h; another group of cells was transfected with the *Nckap1l* overexpression vector and directly cultured in 6‐well plates. When the confluence was more than 80%, the tip of a 20 µL pipette was used to make a linear scratch across the surface of each well. Then, the used medium was discarded, three PBS washes were performed, and the wells were then replenished with serum‐free medium. Microscopic observations and imaging were conducted at 0, 6, 12, and 24 h after the pipette scratch. The scratch area (an indication of cell migration) was quantified using ImageJ software, and the results of different groups were compared.

### Immunocytochemical Staining

Cells were prepared by washing in PBS and fixation in 4% formaldehyde for 15 min, and then permeabilization with 0.5% Triton X‐100 at room temperature for 20 min. The cells were then treated with a normal goat serum working solution for 1 h at room temperature, followed by overnight incubation with the primary antibody at 4 °C. Then, cells were brought back to room temperature for 30 min and incubated for 1 h with the secondary antibody in darkness using specific dilution ratios (Table S5, Supporting Information). Finally, the slides were mounted with Antifade mounting medium containing DAPI (H‐1200, Vector Laboratories, Burlingham, California, USA) and were visualized using fluorescence microscopy as described above.

### F‐Actin Staining

Frozen tissue sections (thickness: 5 µm) and cells transfected with the pcDNA3.1‐*Hnf3a*‐Flag for 24 h or cells transfected with the *Nckap1l* overexpression vector, were washed twice in PBS, fixed in 4% formaldehyde for 15 min, and then washed in PBS with 0.5% Triton X‐100 for 20 min. These samples were incubated in darkness for 1 h with Alexa Fluor 594‐conjugated phalloidin (1:100, C2205S, Beyotime) to stain for F‐actin. The samples were then washed again in PBS with 0.5% Triton X‐100, mounted, and examined using fluorescence microscopy, as described above. Specific antibody dilution ratios (Table S5, Supporting Information) were used for double‐staining with LTL.

### Luciferase Reporter Assay

Luciferase reporter plasmids, pGL3‐*Nckap1l*‐basic and pGL3‐*Nckap1l‐mut*‐basic, were engineered by embedding a 2000 bp segment upstream of the transcription start sites or with the mutate potential binding sites of the *Nckap1l* gene, and were synthesized by Jinkairui Biotechnology Co., Ltd. (Wuhan, China). jetPRIME was used for transfection. Thus, TKPTS cells in 24‐well plates were simultaneously transfected with 300 ng of pGL3‐*Nckap1l*‐basic or pGL3‐*Nckap1l‐mut*‐basic and either 300 ng of pcDNA3.1‐*Hnf3a*‐Flag or pcDNA3.1‐Flag, accompanied by 20 ng of the control plasmid, pRL‐TK (Public Protein/Plasmid Library). Twenty‐four hours after transfection, fluorescence from luciferase and Renilla were measured using the Dual‐Luciferase Reporter Assay System (E1910, Promega). Renilla fluorescence was used as the normalization control for statistical evaluation.

### Transcriptome Sequencing

TKPTS cells were cultured in 6‐well plates and transfected with pcDNA3.1‐*Hnf3a*‐Flag or pcDNA3.1‐Flag. Twenty‐four hours later, the cells were harvested for transcriptome sequencing. The processes of RNA extraction, library preparation, sequencing, and subsequent bioinformatics analysis were performed by Lianchuan Biotech Co., Ltd. (Hangzhou, China).

### CUT&Tag Sequencing Analysis

A total of 100000 TKPTS cells were subjected to CUT&Tag sequencing analysis using the NovoNGS CUT&Tag4.0 High‐Sensitivity Kit for Illumina (N259‐YH01, Novoprotein, Suzhou, China), according to the specified protocol. In brief, cells were collected at room temperature, centrifuged, and the supernatant was removed. After washing, the cells were resuspended in 90 µL of wash buffer and incubated with 10 µL ConA magnetic beads at room temperature for 10 min. An HNF‐3α monoclonal antibody was then added followed by overnight incubation at 4 °C, with details described in Table S4 (Supporting Information). The secondary antibody (N269, Novoprotein, Suzhou, China) was added, followed by incubation at room temperature for 1 h. After washing, ChiTag transposomes were added, the sample was incubated at room temperature for 1 h, followed by fragmentation. The Tagmentation Buffer was added, and the mixture was incubated at 37 °C for 1 h. Then, the stop buffer, proteinase K, and incubation in a bath at 55 °C for 10 min were used to terminate the fragmentation reaction. DNA was then extracted using Tagment DNA Extract Beads, followed by PCR amplification and product purification. The CUT&Tag libraries were sequenced by Genesky Biotechnologies Inc. (Shanghai, China) using the Illumina Novaseq platform.

### Statistical Analysis

Statistical analyses and graphical presentations were performed using GraphPad Prism version 9.0 (GraphPad Software, CA, USA), and results are reported as means ± standard deviations (SDs) in all bar charts. In all cases, the significance of differences between two groups was evaluated using a two‐tailed unpaired Student's *t*‐test. For comparing more than two groups, a one‐way or two‐way ANOVA with Tukey's or Šídák's multiple comparison tests was used. Pearson correlation analysis was used to determine correlations. A *P*‐value below 0.05 was the threshold for statistical significance, and *P*‐values are indicated by *(*p* < 0.05), ** (*p* < 0.01), *** (*p* < 0.001), or **** (*p* < 0.0001).

### Ethics Approval Statement

The China Medical University Ethics Committee approved this research (2024PS1079K), which complied with the ethical standards delineated in the 1964 Helsinki Declaration and its later amendments or comparable ethical norms. All animal protocols followed ethical guidelines and were approved by the Institutional Animal Care and Use Committee of Nanjing Medical University (IACUC‐2204050).

### Patient Consent Statement

Informed consent was secured from all participating patients and their parents.

## Conflict of Interest

The authors declare no conflict of interest.

## Author Contributions

L.H.: data curation; funding acquisition; writing – original draft. Y.G.: formal analysis; methodology. S.X.: methodology; investigation. M.B.: data curation; methodology. W.C.: methodology; investigation. Y.Z.: methodology; investigation; funding acquisition; project administration. Z.J.: conceptualization; supervision; funding acquisition; writing – review & editing. A.Z.: conceptualization; methodology; funding acquisition; writing – review & editing.

## Supporting information



Supporting Information

Supporting Information

Supporting Information

Supporting Information

## Data Availability

The dataset GSE66494 and GSE175759 are adopted from the Gene Expression Omnibus (GEO) public database at https://www.ncbi.nlm.nih.gov/gds. The transcriptome sequencing and CUT&Tag sequencing data supporting this study are openly available in GEO dataset, the accession number is GSE274962 and GSE274963.
