# Supplementary material for: HNF3α Targets Nckap1l and Promotes Renal Fibrosis Following Ischemia‐Reperfusion Injury
Source: Adv Sci (Weinh). 2025 Mar 17;12(19):2410764. doi: 10.1002/advs.202410764 (PMC12097113; doi:10.1002/advs.202410764)
Supplement: Supplementary file 1 — Supporting Information [file ADVS-12-2410764-s002.docx]

**HNF3α targets Nckap1l and promotes renal fibrosis following ischemia-reperfusion injury**

*Ling Hou^*^, Yan Guo, Shuang Xu, Mi Bai, Weidong Cao, Yue Zhang^*^, Zhanjun Jia^*^, Aihua Zhang^*^*


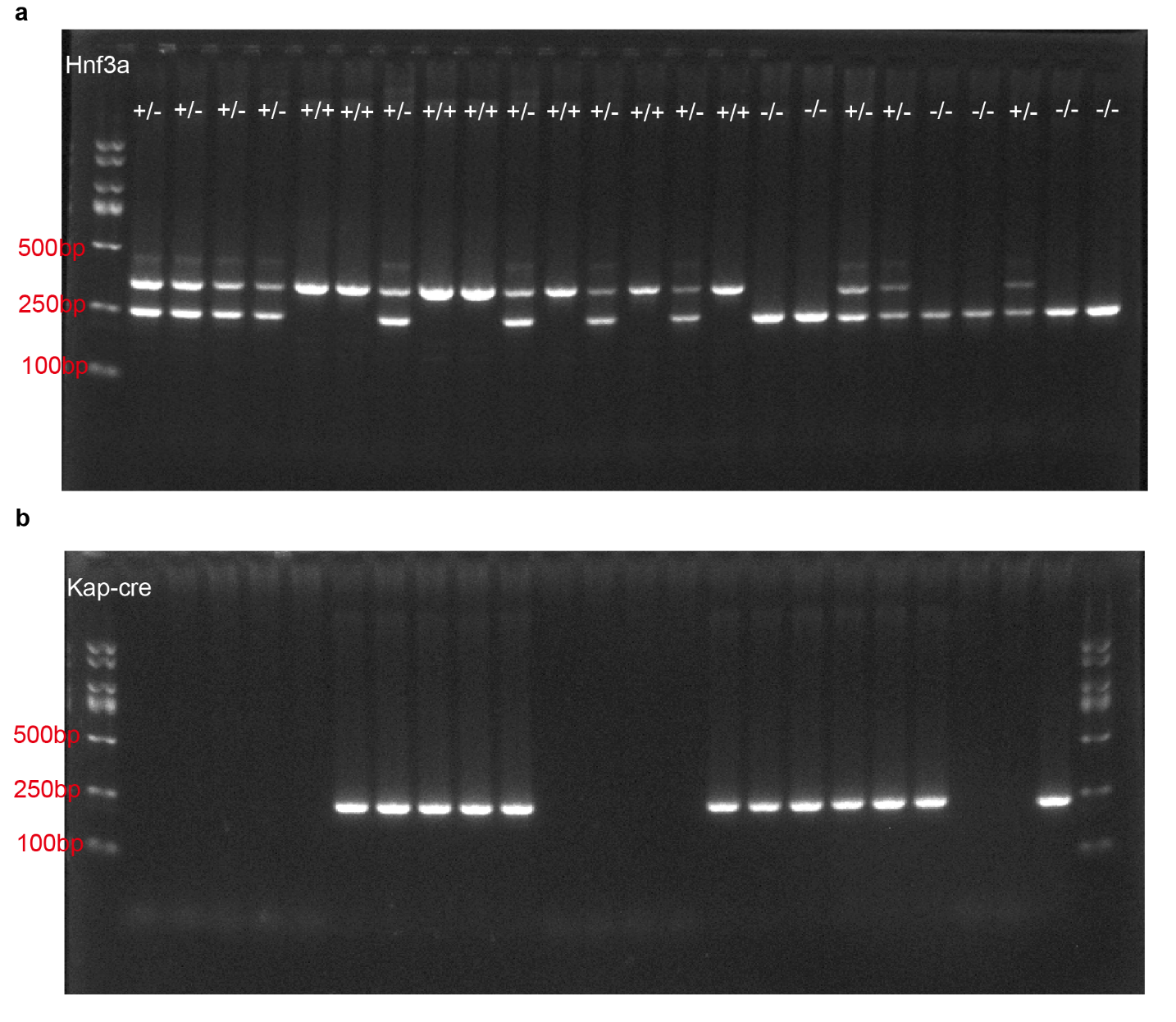


**Supplementary Figure 1. Genotyping of *Hnf3a*-flox mice and Kap-Cre mice.**


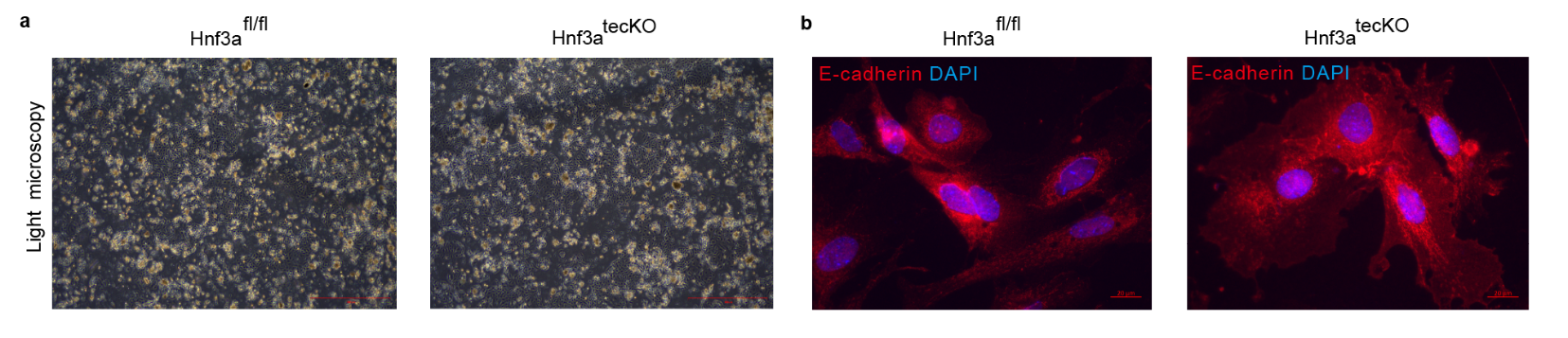


**Supplementary Figure 2. Primary culture of renal tubular epithelial cells from *Hnf3a*^fl/fl^ and *Hnf3a*^tecCKO^ mice.** (a) Representative images of primary renal tubular epithelial cells on day-5 (light microscopy, scale bars: 200 μm). (b) Representative immunofluorescence images of E-cadherin on day-5 (scale bars: 20 μm).


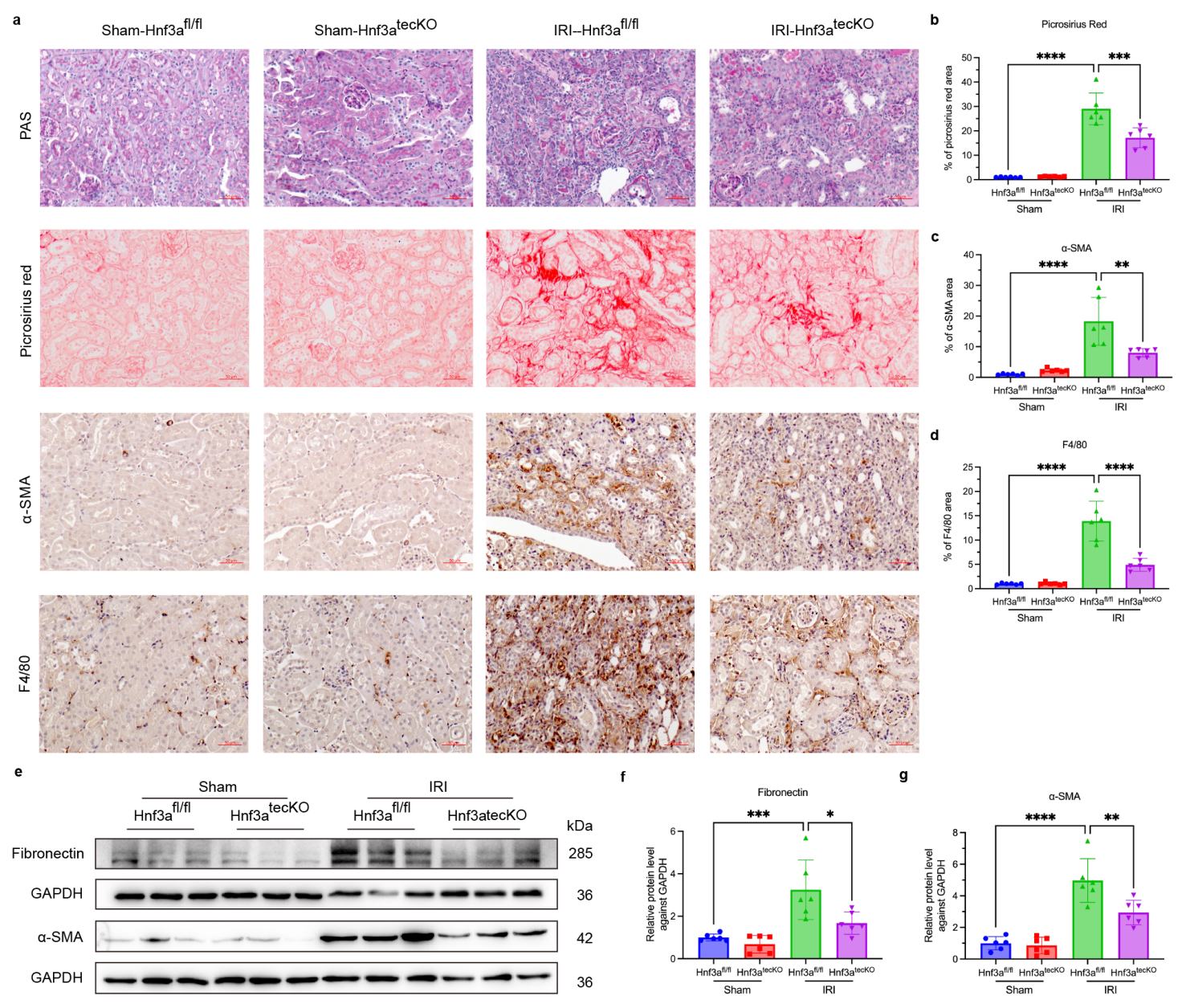


**Supplementary Figure 3. HNF3α Knockout Mitigates Renal Fibrosis in Mice at 3 weeks post-IRI.** (a) Histology images at 3 weeks after renal IRI in the Sham-*Hnf3a*^fl/fl^, Sham-*Hnf3a*^tecCKO^, IRI-*Hnf3a*^fl/fl^ and IRI-*Hnf3a*^tecCKO^ groups based on PAS staining, Sirius Red staining, immunohistochemistry of α-SMA and F4/80 (scale bars: 50 μm) (n=6). (b-d) Quantitative analysis of results from Sirius Red, α-SMA, and F4/80 staining. A one-way ANOVA followed by Tukey’s multiple comparisons test was used for comparisons. (e-g) Immunoblotting of fibronectin and α-SMA in the different groups, and quantitative analysis(n=6). A one-way ANOVA followed by Tukey’s multiple comparisons test was used for comparisons.


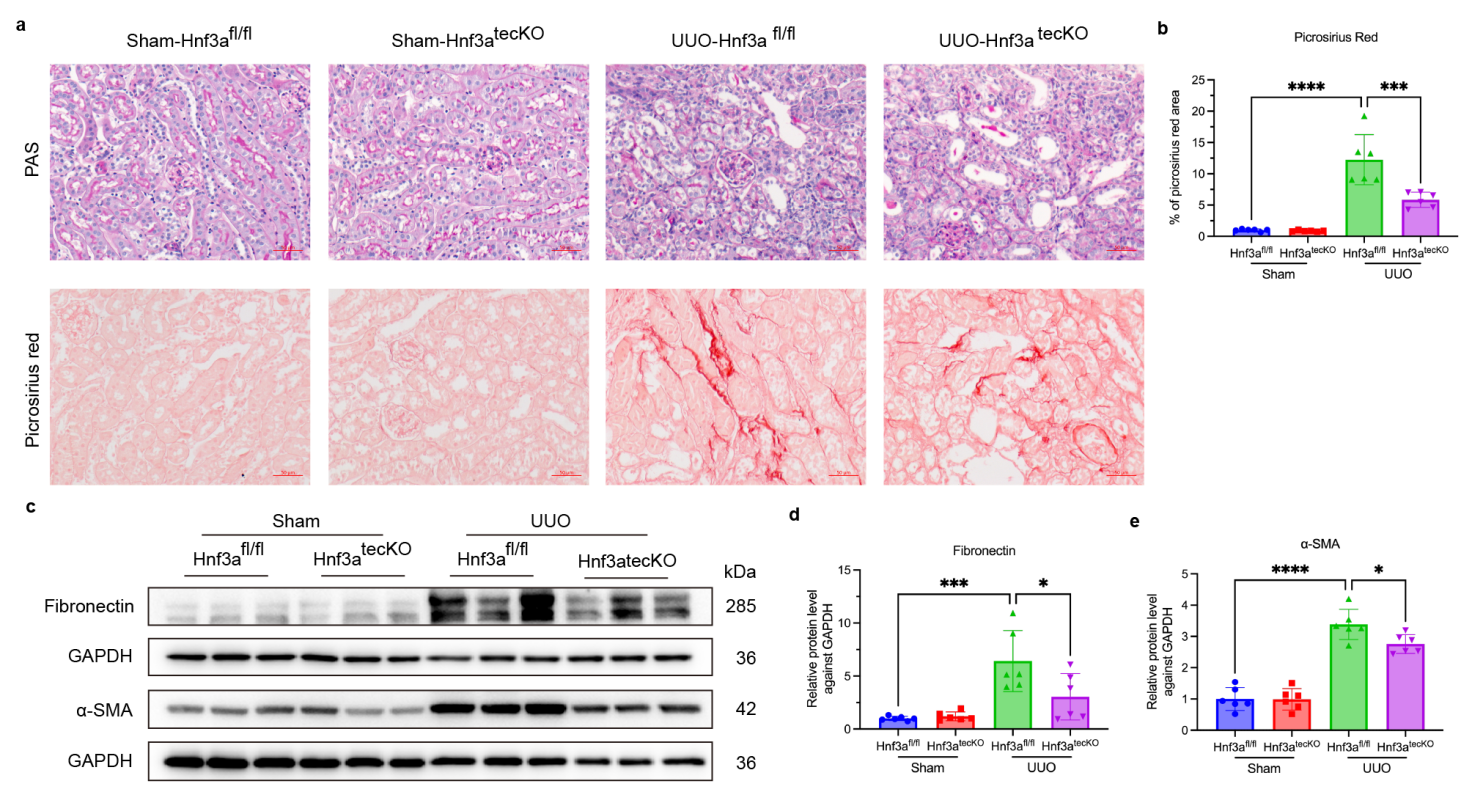


**Supplementary Figure 4. HNF3α Knockout Mitigates Renal Fibrosis in Mice with UUO.** (a) Histology images at 7 days after renal UUO in the Sham-*Hnf3a*^fl/fl^, Sham-*Hnf3a*^tecCKO^, IRI-*Hnf3a*^fl/fl^ and IRI-*Hnf3a*^tecCKO^ groups based on PAS staining and Sirius Red staining (scale bars: 50 μm) (n=6). (b) Quantitative analysis of results from Sirius Red staining. A one-way ANOVA followed by Tukey’s multiple comparisons test was used for comparisons. (c-e) Immunoblotting of fibronectin and α-SMA in the different groups, and quantitative analysis(n=6). A one-way ANOVA followed by Tukey’s multiple comparisons test was used for comparisons.


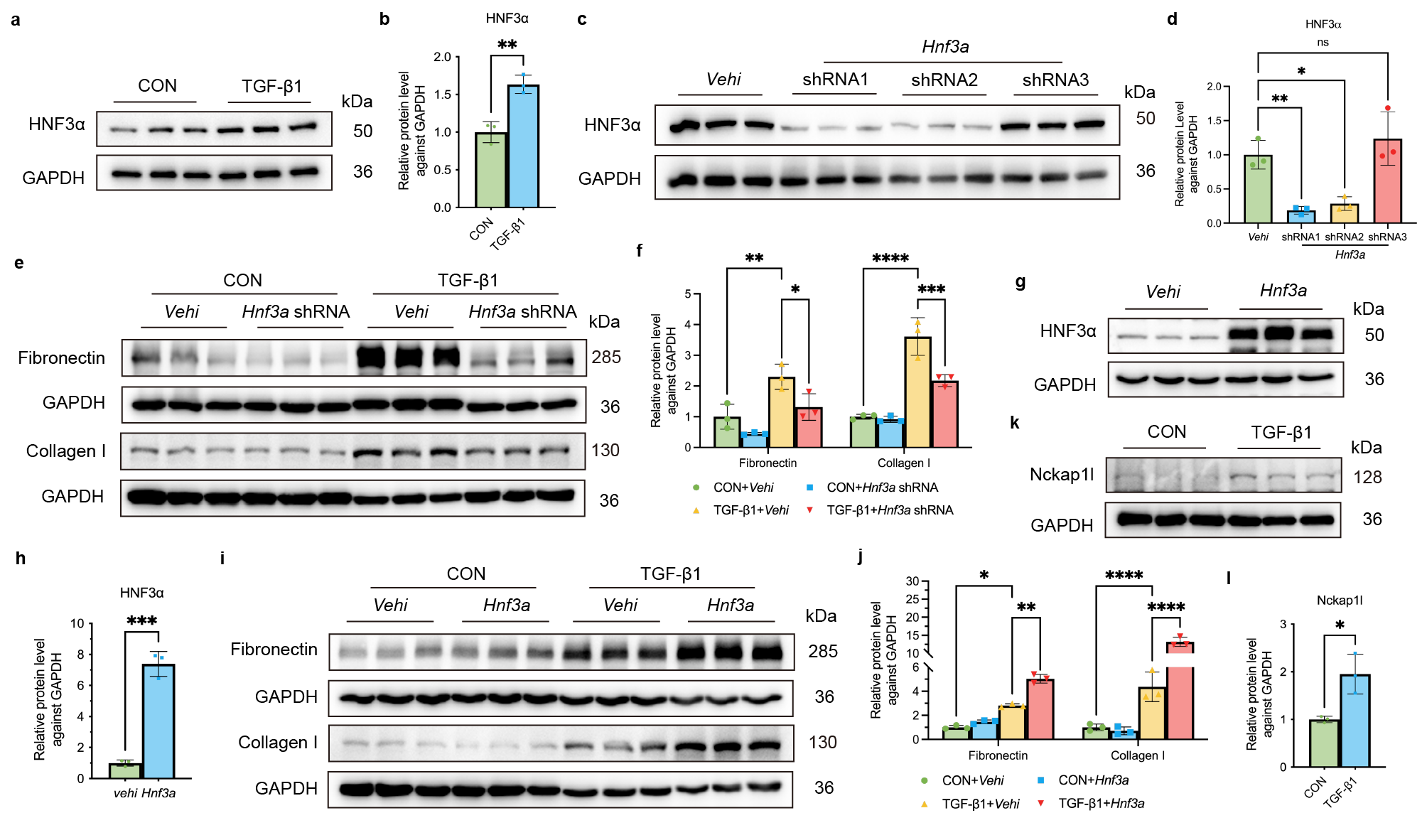


**Supplementary Figure 5. HNF3α mediates the TGF-β1-induced pro-fibrotic response in HK-2 cells.** (a, b) Immunoblotting of HNF3α in HK-2 cells at 24 h after TGF-β1 stimulation, and quantitative analysis (3 per group). (c, d) Immunoblotting of HNF3α in different groups of HK-2 cells at 24 h after transfection, and quantitative analysis (3 per group). One-way ANOVA followed by Tukey’s multiple comparisons test was used for comparisons. (e, f) Immunoblotting of fibronectin and Collagen I in different groups of HK-2 cells, and quantitative analysis (3 per group). One-way ANOVA followed by Tukey’s multiple comparisons test was used for comparisons. (g, h) Immunoblotting of HNF3α in HK-2 cells that were transfected with an *Hnf3a* overexpression plasmid or control plasmid, and quantitative analysis (3 per group). (i, j) Immunoblotting of fibronectin and Collagen I in different groups of HK-2 cells, and quantitative analysis (3 per group). One-way ANOVA followed by Tukey’s multiple comparisons test was used for comparisons. (k, l) Immunoblotting of Nckap1l in HK-2 cells at 24 h after TGF-β1 stimulation, and quantitative analysis (3 per group).


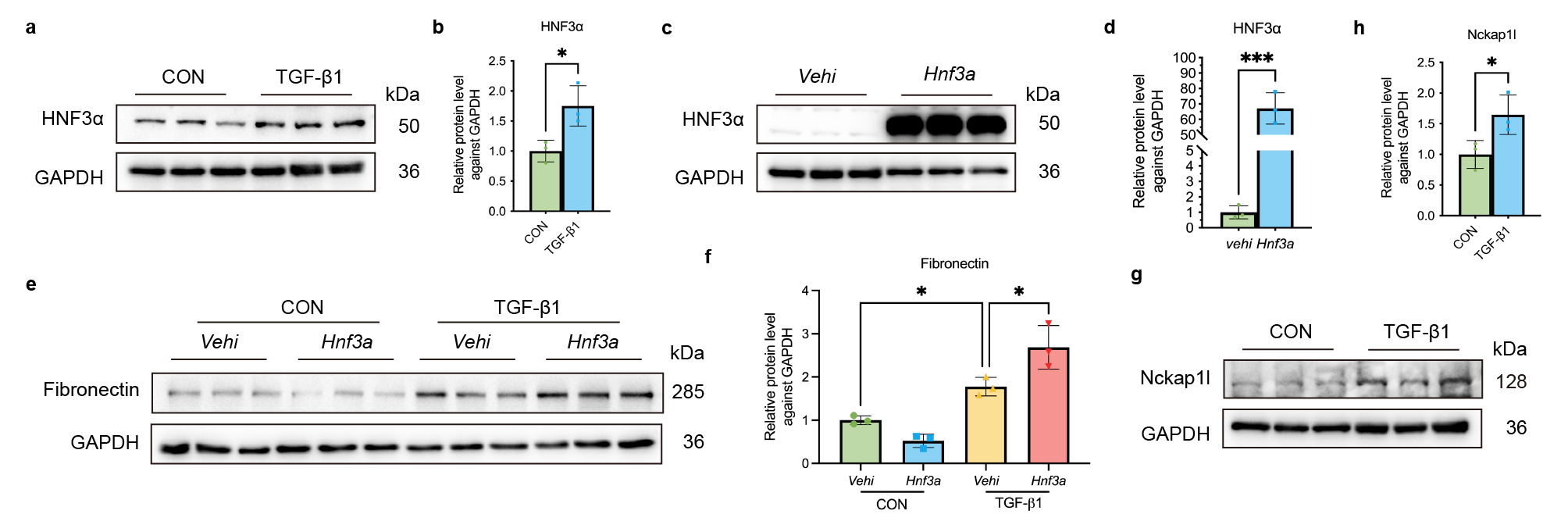


**Supplementary Figure 6. HNF3α promote the TGF-β1-induced pro-fibrotic response in NRK49F cells.** (a, b) Immunoblotting of HNF3α in NRK49F cells at 24 h after TGF-β1 stimulation, and quantitative analysis (3 per group).(c, d) Immunoblotting of HNF3α in NRK49F cells that were transfected with an *Hnf3a* overexpression plasmid or control plasmid, and quantitative analysis (3 per group). (e, f) Immunoblotting of fibronectin in different groups of NRK49F cells, and quantitative analysis (3 per group). One-way ANOVA followed by Tukey’s multiple comparisons test was used for comparisons. (k, l) Immunoblotting of Nckap1l in NRK49F cells at 24 h after TGF-β1 stimulation, and quantitative analysis (3 per group).


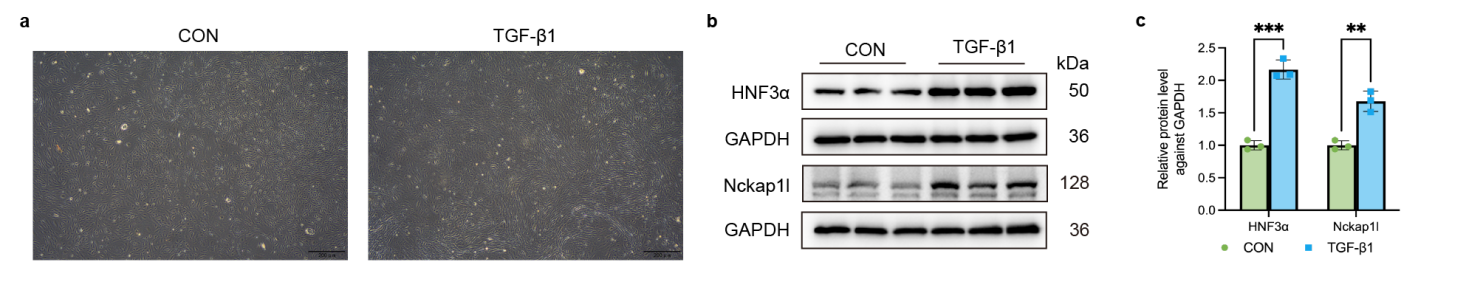


**Supplementary Figure 7. The expression of HNF3α and Nckap1l in primary proximal tubular cells of Hnf3α^fl/fl^ mice after TGF-β1stimulation.** (a) Images of primary proximal tubular cells of Hnf3α^fl/fl^ mice after TGF-β1stimulation. (b, c) Immunoblotting of HNF3α and Nckap1l in primary proximal tubular cells of Hnf3α^fl/fl^ mice at 24 h after TGF-β1 stimulation, and quantitative analysis (3 per group).Two-tailed unpaired t-test was used to determine the p-values.


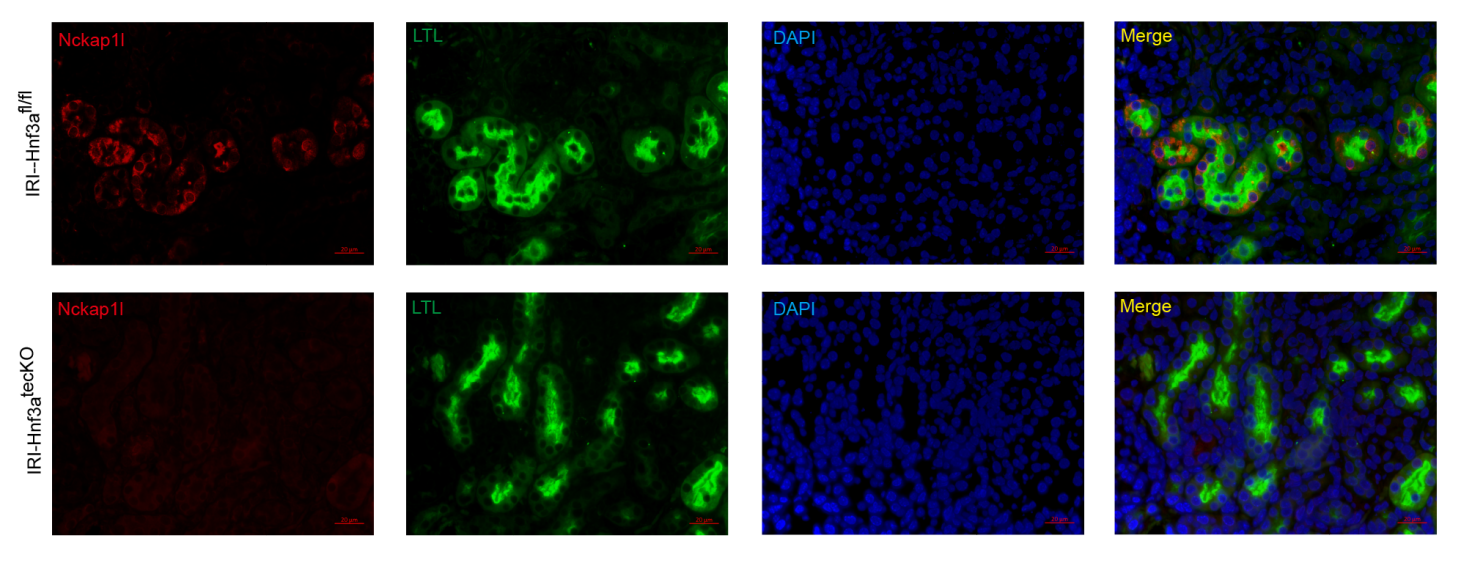


**Supplementary Figure 8. Immunofluorescence images of Nckap1l** **and LTL in the IRI+*Hnf3a*^tecCKO^ group and the IRI+*Hnf3a*^fl/fl^ control group.**


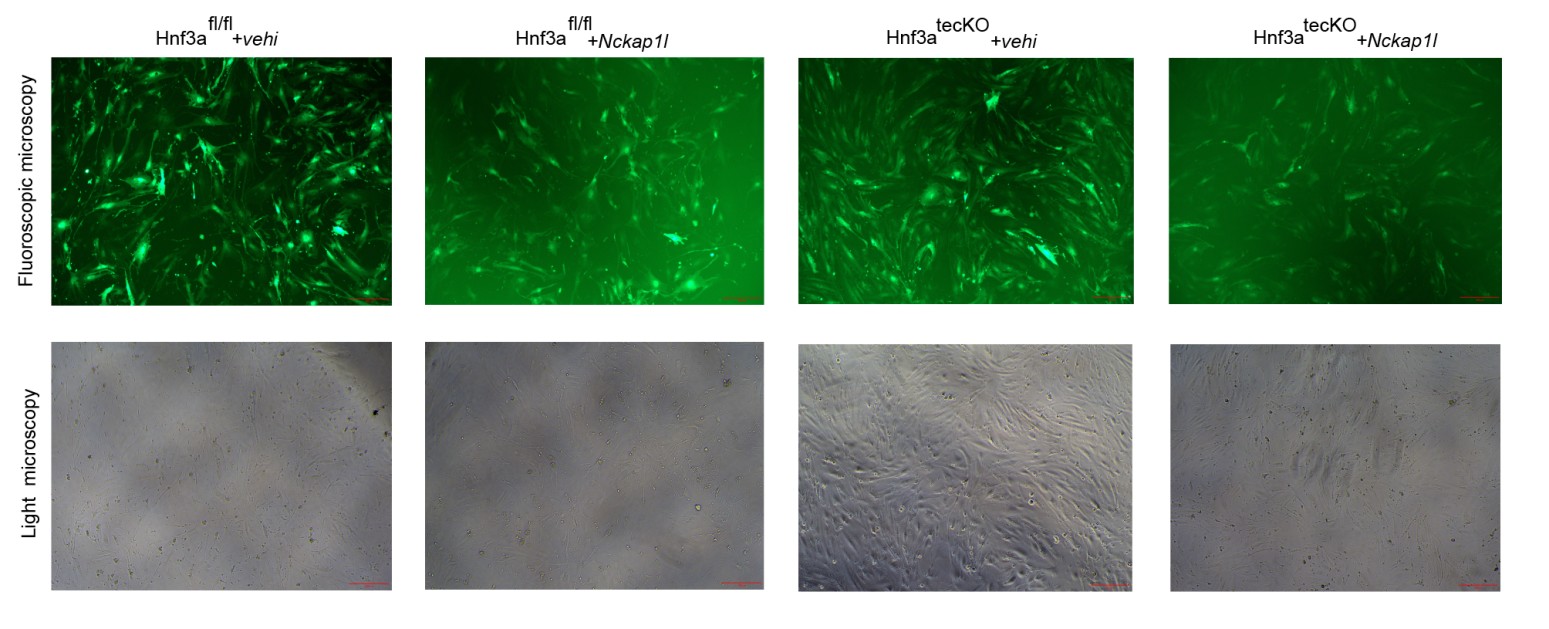


**Supplementary Figure 9. Primary renal tubular epithelial cells transfected with *Nckap1l* lentiviral overexpression vector.** Representative fluorescence microscopy and bright-field microscopy of primary renal tubular epithelial cells at 72 h after transfection with the *Nckap1l* lentiviral overexpression vector or control vector (scale bars: 200 μm).


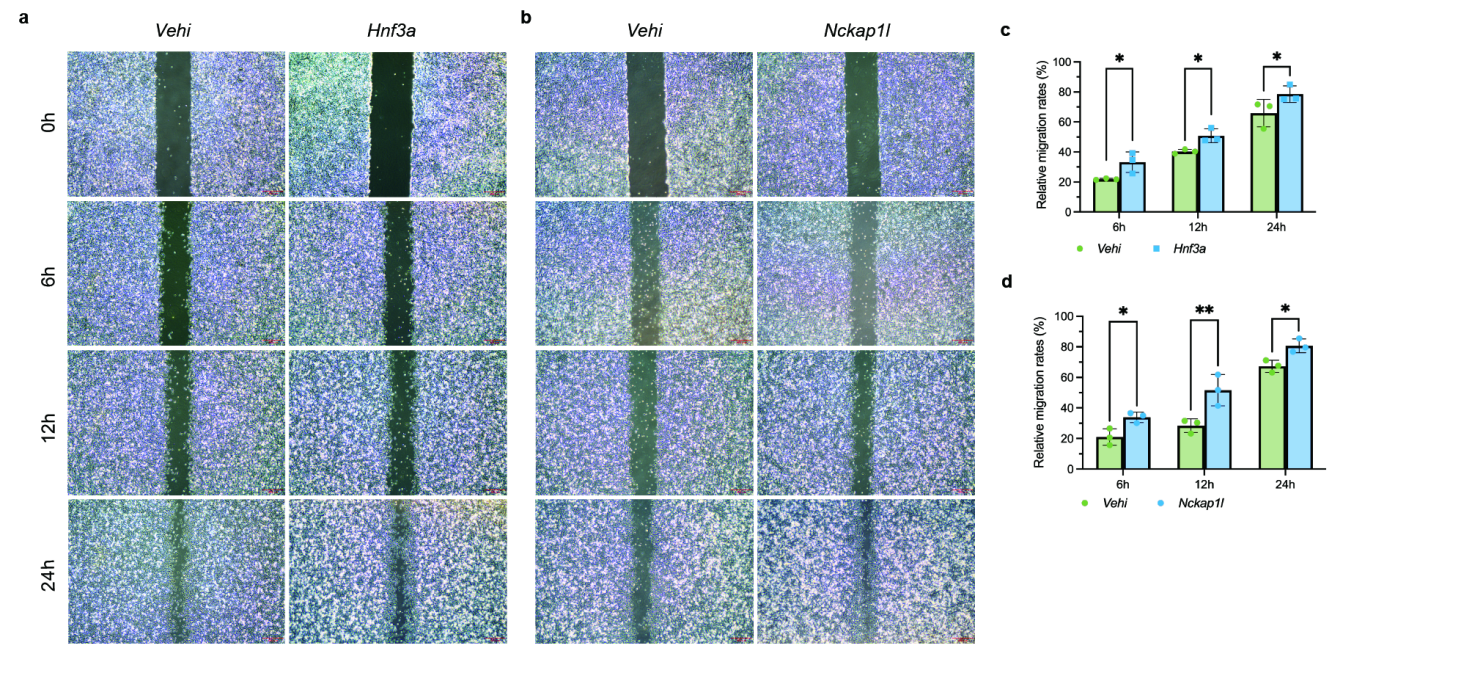


**Supplementary Figure10. Cell scratch images.** (a–d) Cell scratch assay in the Vehi and *Hnf3a* groups, and in the in Vehi and *Nckap1l* groups, and quantitative analysis (scale bars: 400 μm, 3 per group). A pair-wise *t*-test was used for comparisons.


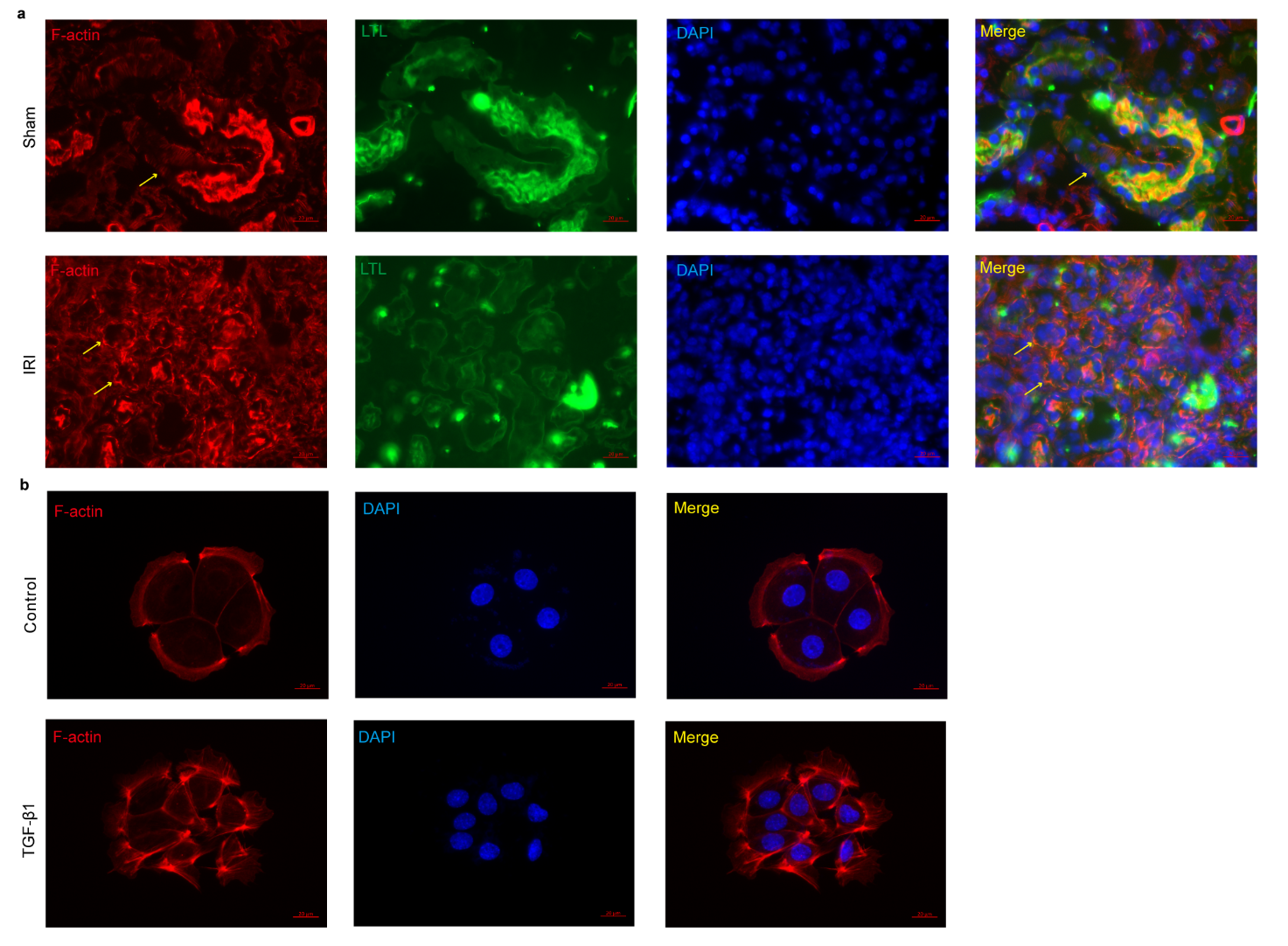


**Supplementary Figure 11. Polymerization of F-actin at the periphery of renal tubular epithelial cells in the mouse IRI model after TGF-β1 stimulation.** (a) Representative images of F-actin and LTL (dual staining) in the mouse IRI model and sham groups (scale bars: 20 μm). (b) Representative images of F-actin in TKPTS cells in the TGF-β1 and control groups (scale bars: 20 μm).


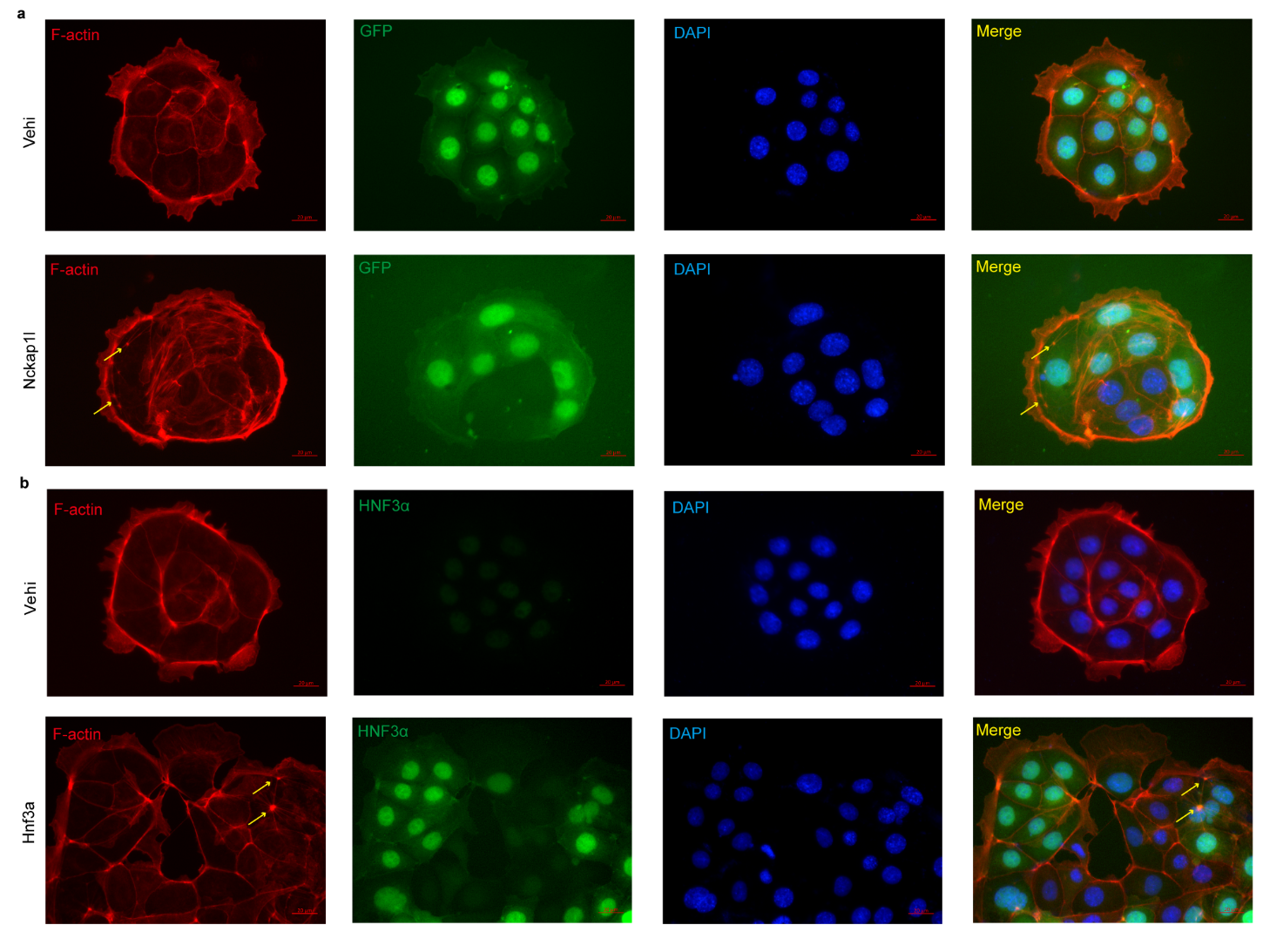


**Supplementary Figure 12. Effect of overexpression of *Nckap1l* or *Hnf3a* on polymerization of F-actin at the periphery of TKPTS cells.** (a) Representative images of F-actin and GFP autofluorescence in the Vehi and *Nckap1l* groups (scale bars: 20 μm). (b) Representative images of F-actin and HNF3α (double staining) in renal tubular epithelial cells in the Vehi and *Hnf3a* groups (scale bars: 20 μm).

**Supplementary Table 1.** Clinical characteristics of the 20 CKD patients.^*^

| **Characteristic** | **N (%) or mean±SD** |
| --- | --- |
| Male, n (%) | 11 (55%) |
| Age (years) | 11.2±1.6 |
| Pathologic diagnosis |  |
| IgA nephropathy | 7 (35%) |
| Lupus nephritis | 4 (20%) |
| Henoch-Schönlein purpura nephropathy | 3 (15%) |
| Crescentic glomerulonephritis | 2 (10%) |
| Focal segmental glomerulosclerosis | 1 (5%) |
| Minimal change disease | 1 (5%) |
| Interstitial nephritis | 1 (5%) |
| Thrombotic microangiopathy | 1 (5%) |
| Blood urea nitrogen (mmol/L) | 12.06±9.87 |
| Creatinine (μmol/L) | 161.8±186.6 |

**Supplementary Table 2.** Primers used for quantitative PCR and genotyping.

| Primer | Sequence (5'-3') |
| --- | --- |
| *mHnf3a-F* | TGGACTTCAAGGCATACGAGC |
| *mHnf3a-R* | GCACGGGTCTGGAATACACA |
| *mNckap1l-F* | TGTCCGAAATAGCACGCAACA |
| *mNckap1l-R* | ATCCCGAAATTCCATGACATCC |
| *mGapdh-F* | AGGTCGGTGTGAACGGATTTG |
| *mGapdh-R* | TGTAGACCATGTAGTTGAGGTCA |
| *mNckap1l-P-F1* | GACTTTCTCAACCCTTGCAACC |
| *mNckap1l-P-R1* | GGACCCTGGTCTCTTTCTAACT |
| *mNckap1l-P-F2* | CAGGCATTCTTGACCGGGAT |
| *mNckap1l-P-R2* | CGGCTCCTCACAATGCAGTA |
| *T017857-F1* | ATTTGTCACGTCCTGCACGA |
| *T017857-R1* | TAGTCCTCTCTCCAAGATCGCAC |
| *T017857-F2* | CGAAAGTCTAACTTCGGGTGCAG |
| *T017857-R2* | CACAGAGAGCGTAGACGCAGAAC |
| *Kap-F* | AGATGCCAGGACATCAGGAACCTG |
| *Kap-R* | ATCAGCCACACCAGACACAGAGATC |

**Supplementary Table 3.** shRNA guide sequences.

| Gene | shRNA # | Sequence (5’-3’) |
| --- | --- | --- |
| *mHnf3a* | 1 | GCTGTCCTTCAACGATTGTTT |
|  | 2 | GCTGGACTTCAAGGCATACGA |
|  | 3 | GCCTTACTCCTACATCTCGCT |
|  | Negative control | CCTAAGGTTAAGTCGCCCTCG |
| *hHNF3A* | 1 | GCGTACTACCAAGGTGTGTAT |
|  | 2 | CTGTCCTTCAATGACTGCT |
|  | 3 | CTCCTCCGTCCCGGTCAGCAA |
|  | Negative control | GTTCTCCGAACGTGTCACGTT |

**Supplementary Table 4.** siRNA sequences.

| Gene | siRNA # | Sequence (5’-3’) |
| --- | --- | --- |
| *mNckap1l* | 1-sense | GGAGUUGGAGACUGUGUUATT |
|  | 1-Anti-sense | UAACACAGUCUCCAACUCCTT |
|  | 2-sense | GUCUCAAGUACAUCAACAATT |
|  | 2-Anti-sense | UUGUUGAUGUACUUGAGACTT |
|  | 3-sense | GGUGCAGAUGCUUCCAGAATT |
|  | 3-Anti-sense | UUCUGGAAGCAUCUGCACCTT |
|  | NC-sense | UUCUCCGAACGUGUCACGUTT |
|  | NC-Anti-sense | ACGUGACACGUUCGGAGAATT |

**Supplementary Table 5.** Antibodies used for different applications.

| **Antibody** | **Company** | **Cat. No** | **Species** | **Dilution (application)** |
| --- | --- | --- | --- | --- |
| HNF-3α (A-3) | Santa Cruz | sc-514695 | Mouse | 1:2500 (WB), 1:50(ICC) |
| HNF-3α (Q-6) | Santa Cruz | sc-101058 | Mouse | 1:100 (IHC) |
| HNF-3α | Abcam | Ab170933 | Rabbit | 1 µg/5 µg chromatin (ChIP), 1:100(IF) |
| HNF-3α | Abcam | Ab55178 | Mouse | 1:1000(WB), 1:100 (ICC) |
| FLAG | Sigma | F1804 | Mouse | 1:1000 (WB) |
| Fibronectin | Abcam | ab2413 | Rabbit | 1:1000 (WB), 1:50 (IHC) |
| Collagen ǀ | Bioss | bs-10423R | Rabbit | 1:1000 (WB) |
| Collagen ǀ | Cell Signaling Technology | #72026 | Rabbit | 1:200 (IHC) |
| α-SMA | Abcam | ab7817 | Mouse | 1:1000 (WB) |
| α-SMA | Abcam | ab5694 | Rabbit | 1:200 (IHC) |
| F4/80 | Cell Signaling Technology | #70076 | Rabbit | 1:250 (IHC) |
| CD86(D-6) | Santa Cruz | sc-28347 | Mouse | 1:1000 (WB), 1:100(IHC) |
| CD206 | Proteintech | 18704-1-AP | Rabbit | 1:500 (WB) |
| HEM1 | Novus | NBP2-13643 | Rabbit | 1:1500(WB), 1:200 (IHC), 1:200(IF) |
| GAPDH | Proteintech | HRP-60004 | Mouse | 1:5000 (WB) |
| E-cadherin | Abclonal | A3044 | Rabbit | 1:100 (ICC) |
| LTL | Vector Laboratories | FL-1321-2 | Fluorescein | 1:100 (IF) |
| VECTASHIELD (Antifade mounting medium with DAPI) | Vector Laboratories | H-1200 |  | Concentration of DAPI: 1.5 μg/mL |
| Alexa Fluor 594-conjugated phalloidin | Beyotime | C2205S |  | 1:100 (IF) |
| Anti-rabbit IgG, HRP-linked Antibody | ZSGB-BIO | ZB-2301 |  | 1:5000 (WB) |
| Anti-mouse IgG, HRP-linked Antibody | Cell Signaling Technology | #7076 |  | 1:3000 (WB) |
| Donkey anti-Rabbit IgG (H+L), Alexa Fluor™ 594 | Invitrogen/ThermoFisher Scientific | A21207 | Rabbit | 1:500 (IF) |
| Donkey anti-Mouse IgG (H+L), Alexa Fluor™ 488 | Invitrogen/ThermoFisher Scientific | A21202 | Mouse | 1:500 (IF) |
| Multi-rAb CoraLite® Plus 488-Goat Anti-Rabbit Recombinant Secondary Antibody (H+L) | Proteintech | RGAR002 | Rabbit | 1:200 (IF) |
| Multi-rAb CoraLite® Plus 594-Goat Anti-Mouse Recombinant Secondary Antibody (H+L) | Proteintech | RGAM004 | Mouse | 1:200 (IF) |
