# Supplementary material for: HNF3α Targets Nckap1l and Promotes Renal Fibrosis Following Ischemia‐Reperfusion Injury
Source: Adv Sci (Weinh). 2025 Mar 17;12(19):2410764. doi: 10.1002/advs.202410764 (PMC12097113; doi:10.1002/advs.202410764)
Supplement: Supplementary file 2 — Supporting Information [file ADVS-12-2410764-s004.pdf]

ADVANCED SCIENCE
Open Access

# Supporting Information

for *Adv. Sci.*, DOI 10.1002/advs.202410764

HNF3$\alpha$ Targets Nckap1l and Promotes Renal Fibrosis Following Ischemia-Reperfusion Injury

*Ling Hou*, Yan Guo, Shuang Xu, Mi Bai, Weidong Cao, Yue Zhang*, Zhanjun Jia* and Aihua Zhang**

SUPPLEMENTARY INFORMATION

Figure 1d

The right MARKER from top to bottom are sequentially 180, 130, 100, 70, 55, 40, 35, 25, 15 (ThermoFisher Scientific #26616).

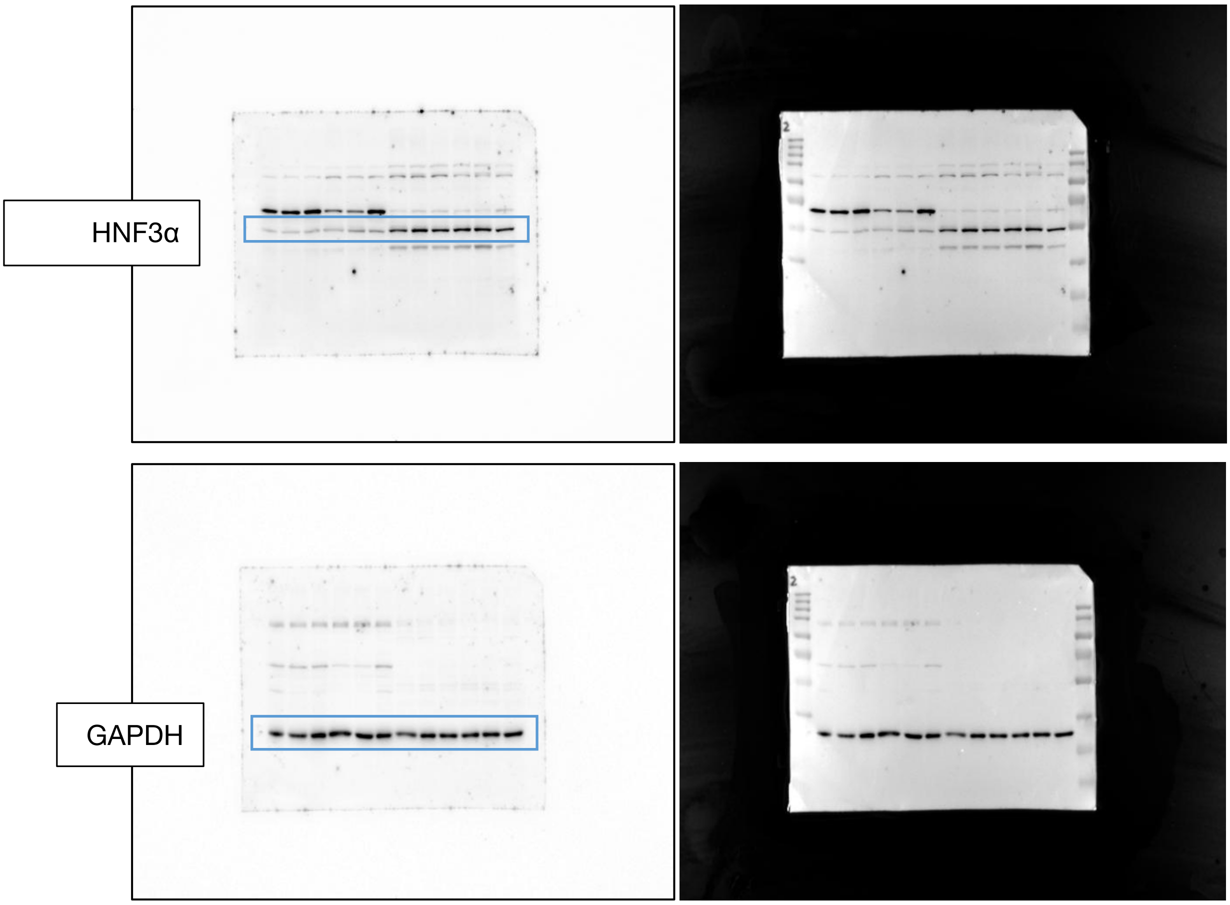

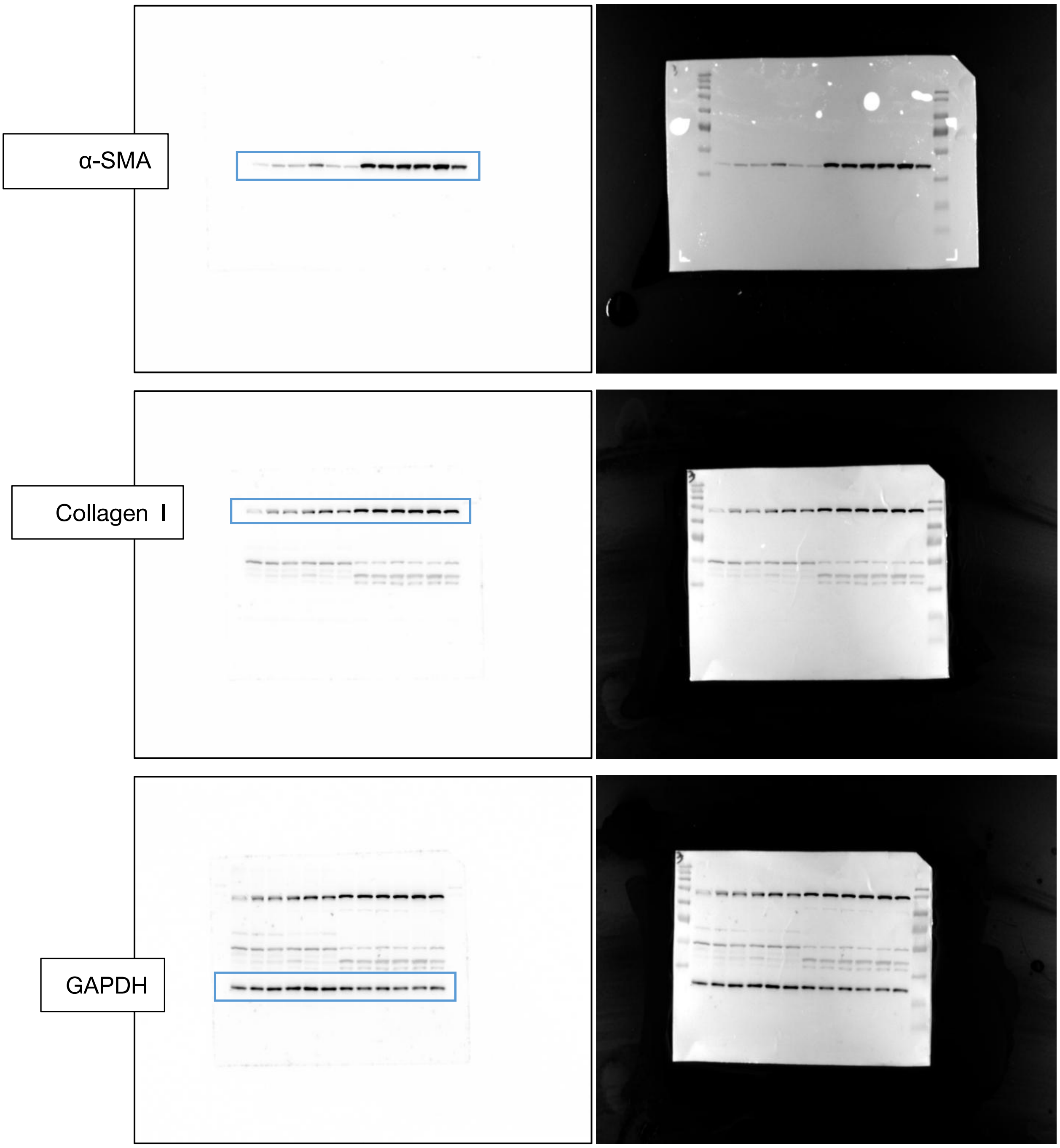
α-SMA
Collagen I
GAPDH

Figure 1h

The MARKER from top to bottom are sequentially 180, 130, 100, 70, 55, 40, 35, 25, 15 (ThermoFisher Scientific #26616).

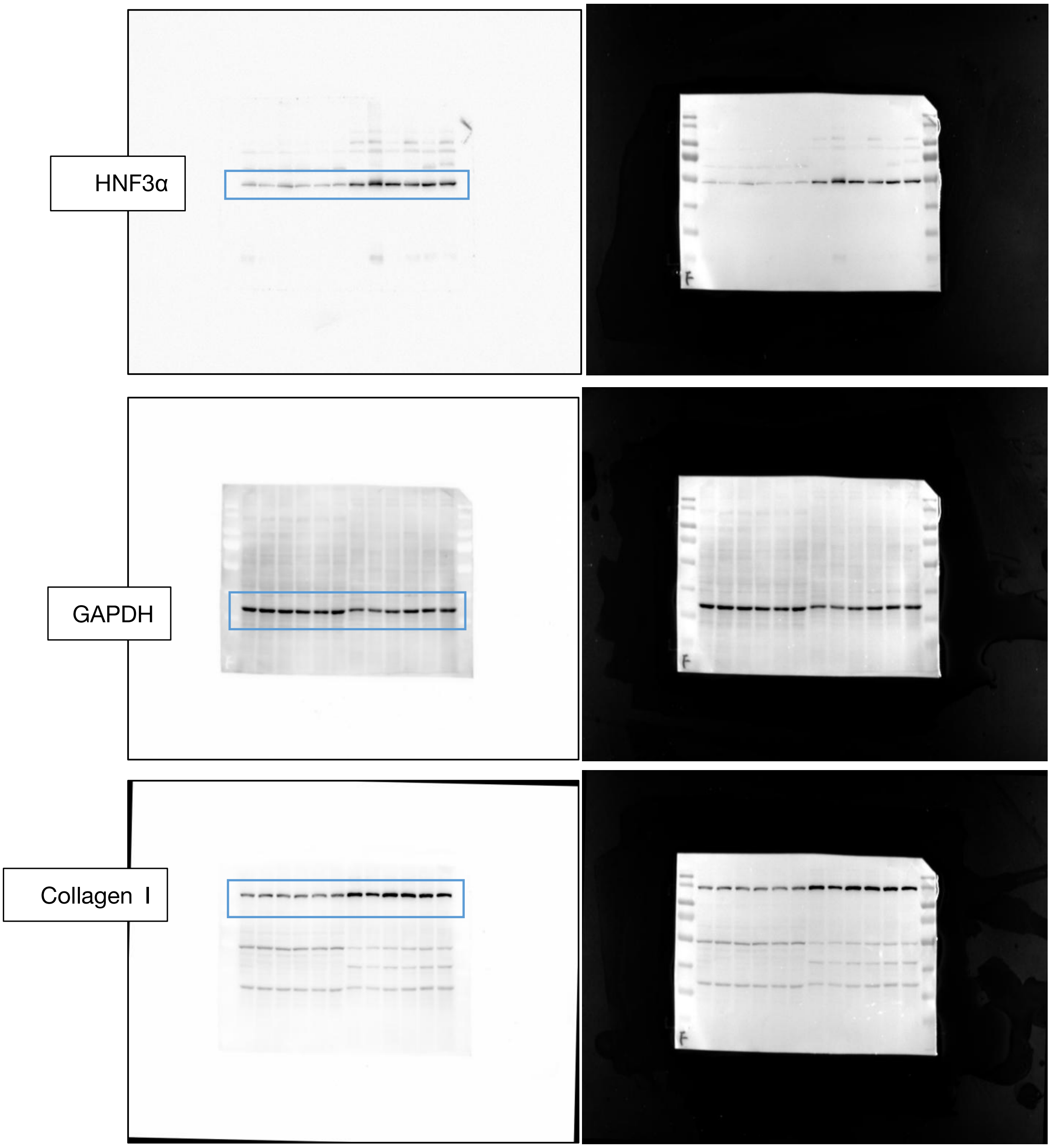

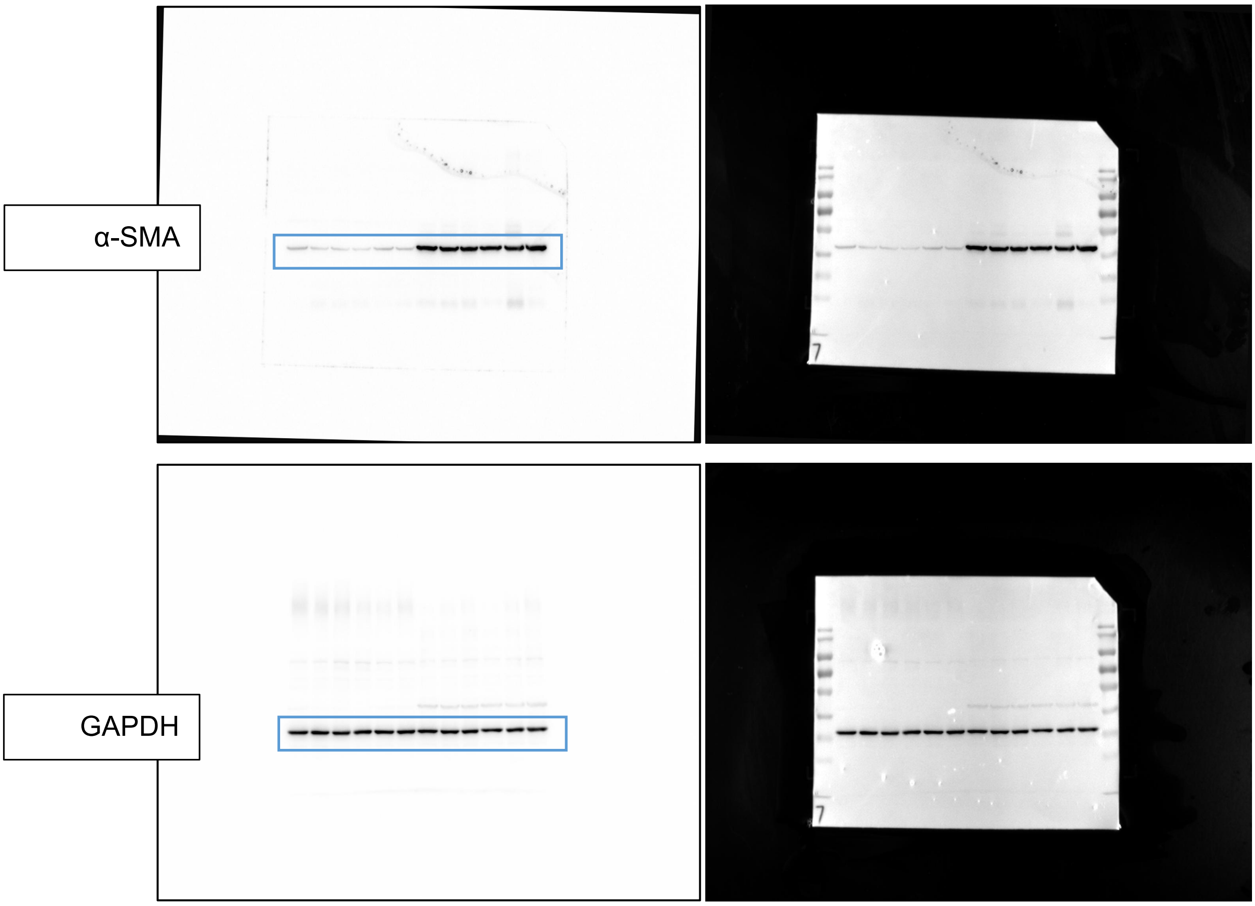
α-SMA
GAPDH

Figure 2b

The MARKER from top to bottom are sequentially 180, 130, 100, 70, 55, 40, 35, 25, 15 (ThermoFisher Scientific #26616).

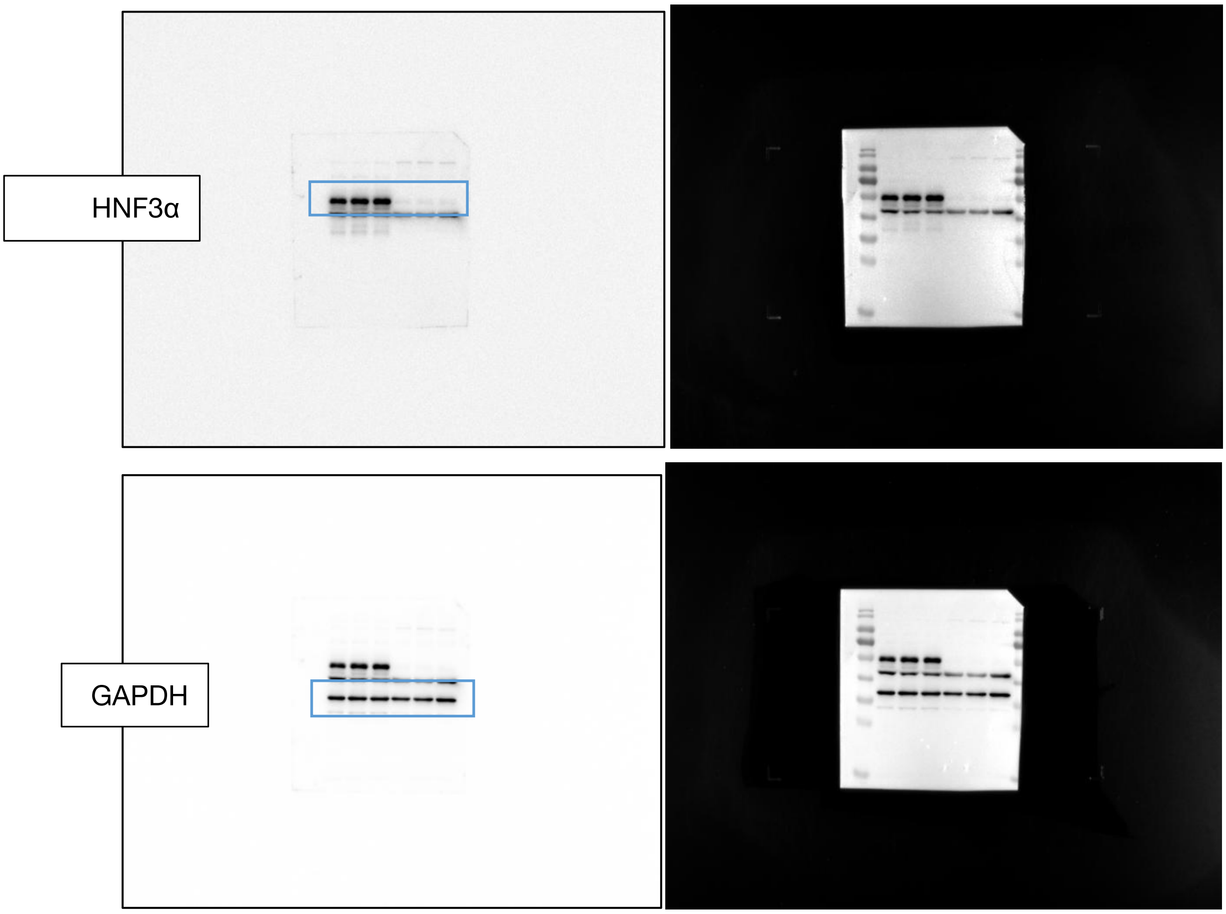

Figure 2g

The MARKER from top to bottom are sequentially 180, 130, 100, 70, 55, 40, 35, 25, 15 (ThermoFisher Scientific #26616).

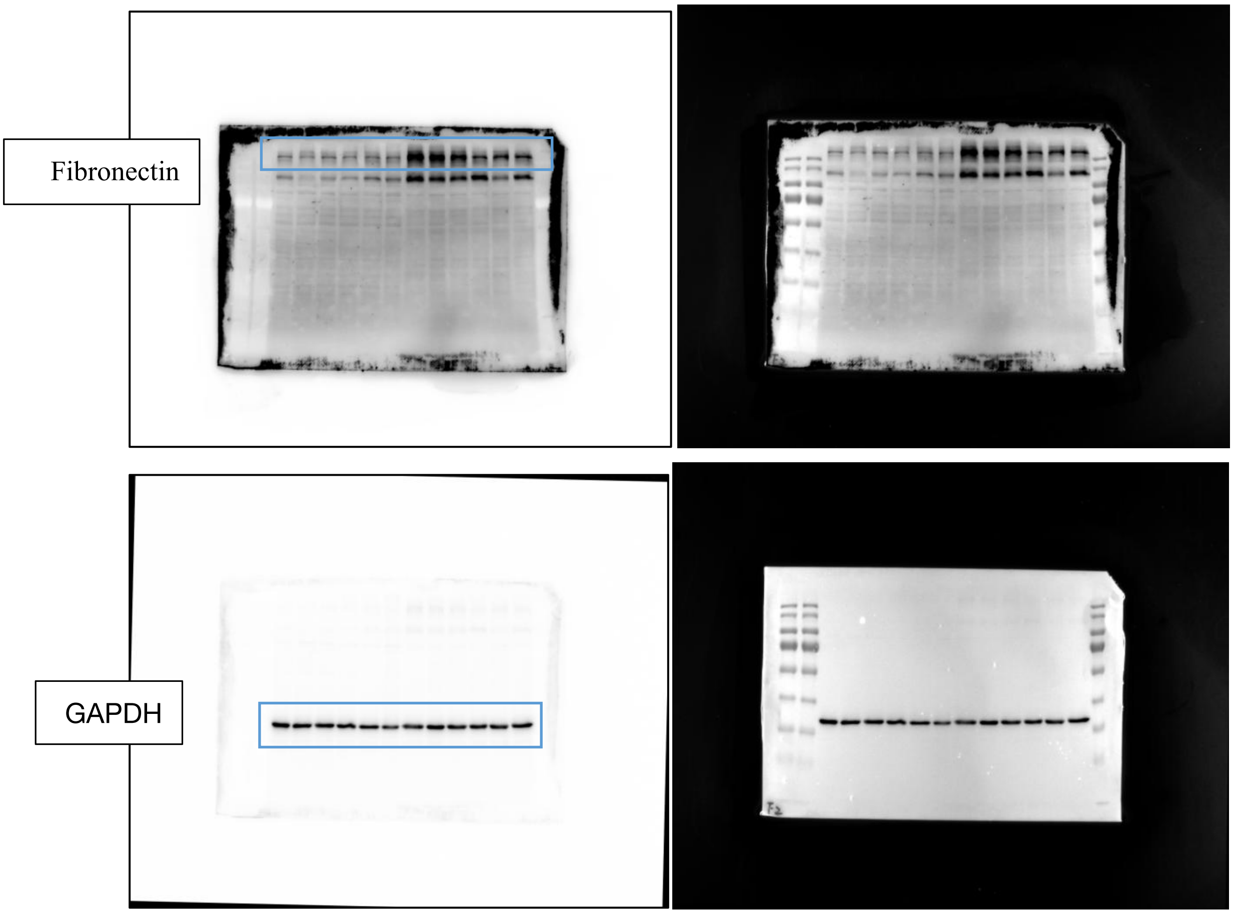

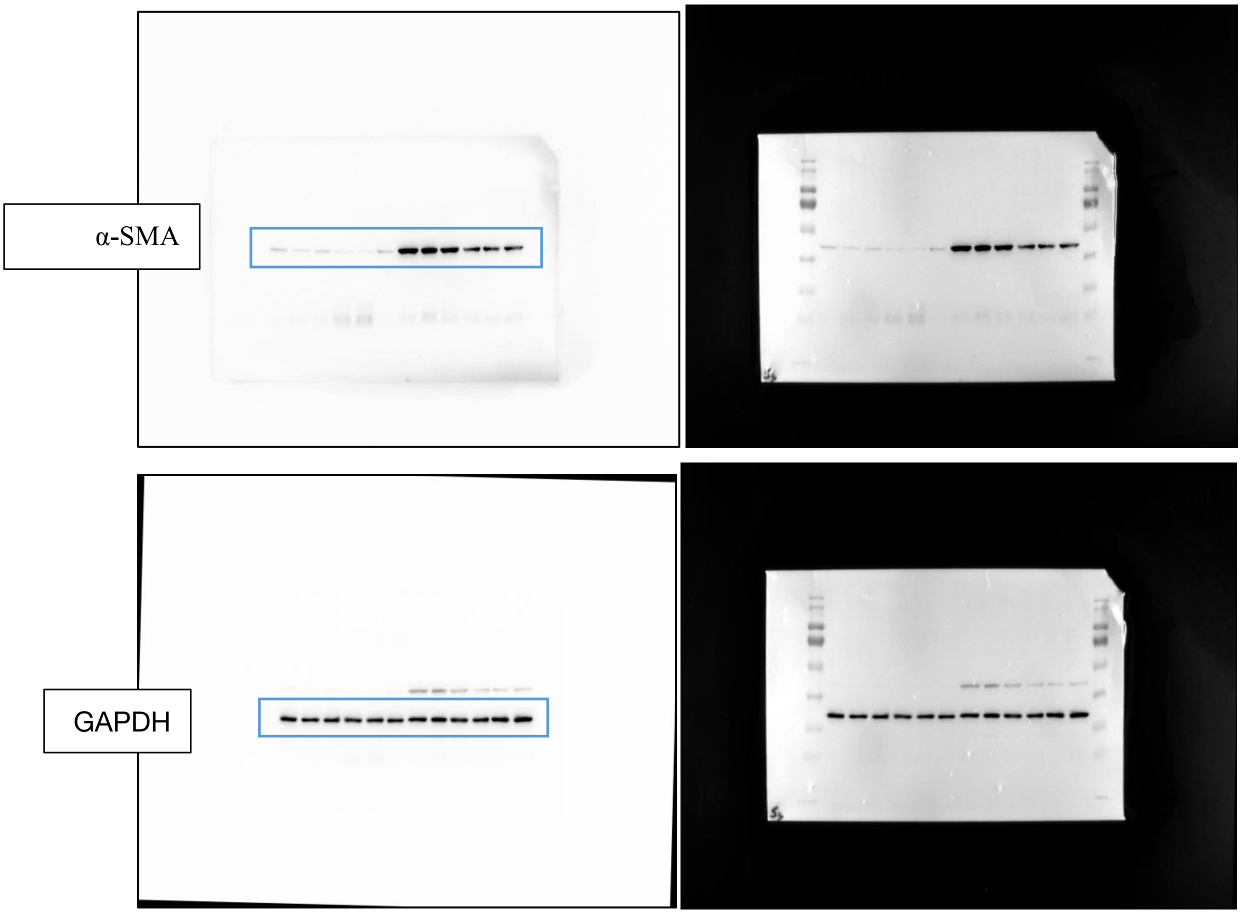
α-SMA
GAPDH

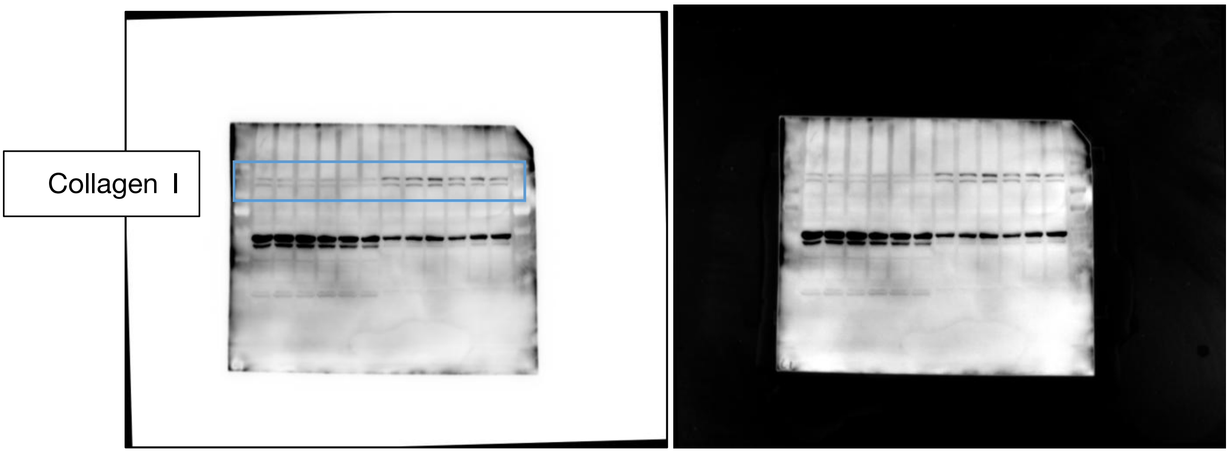
Collagen I

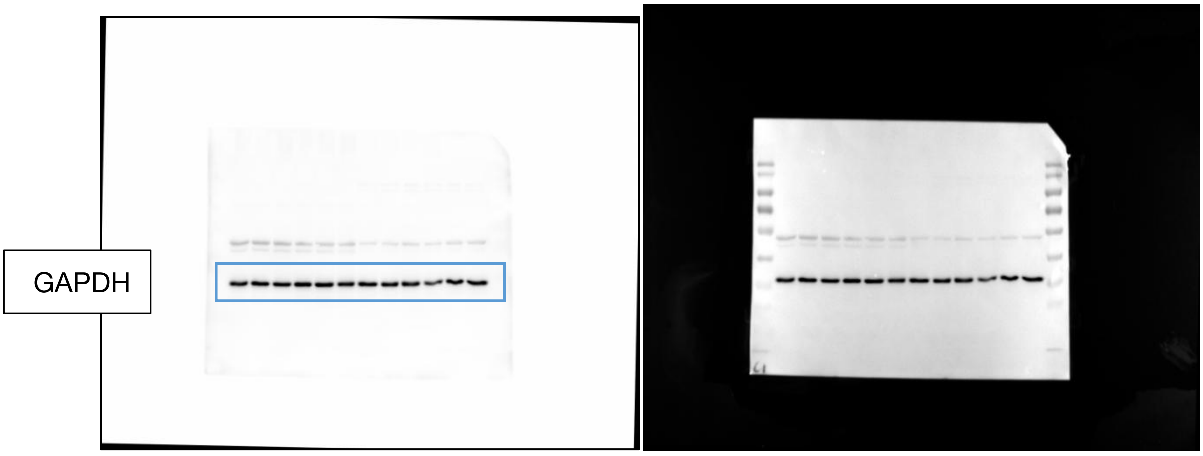
GAPDH

CD86

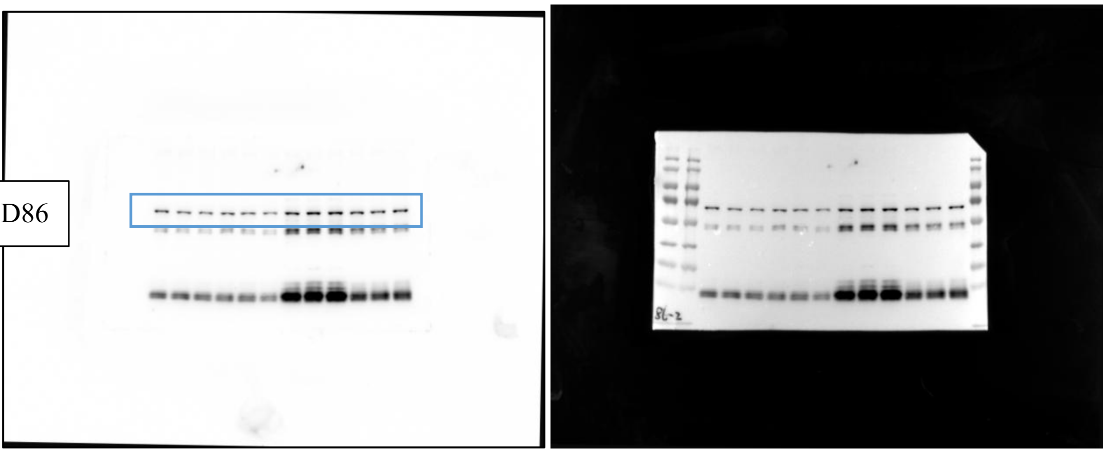

GAPDH

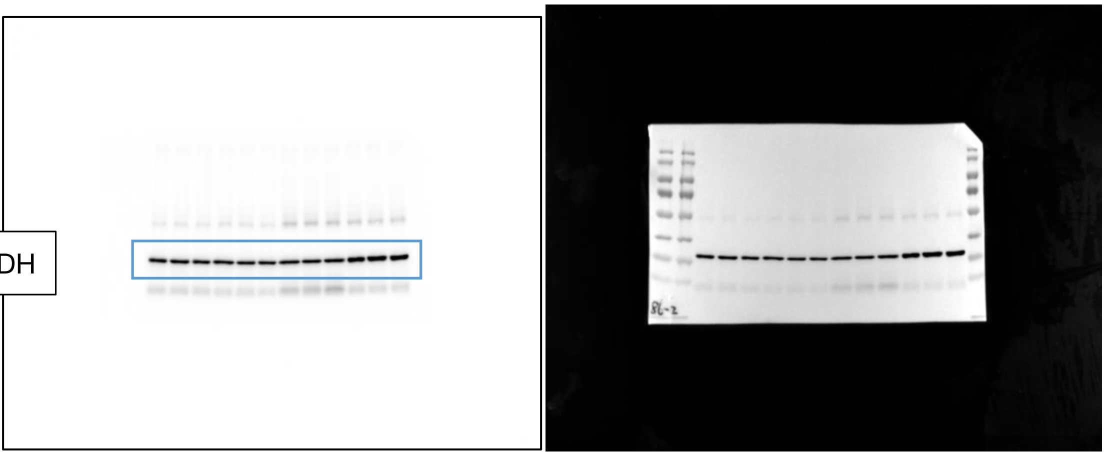

CD206

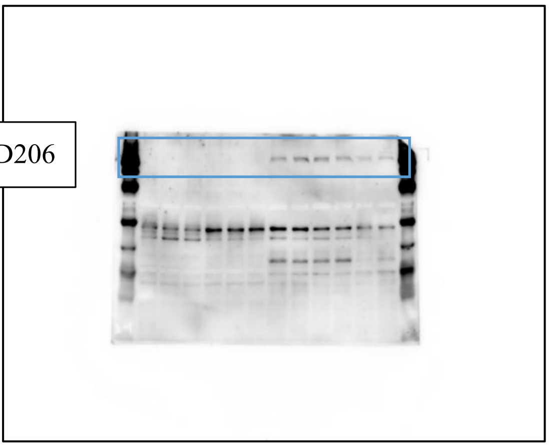

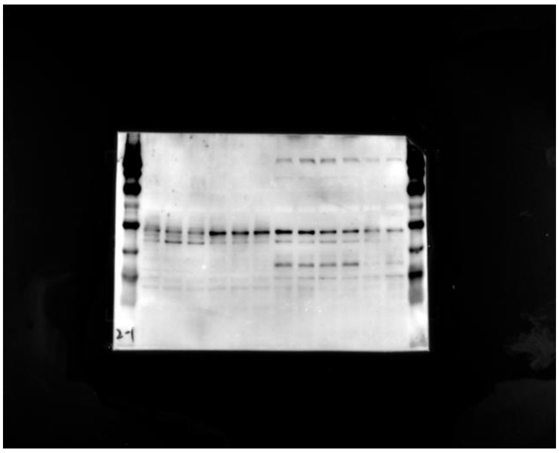


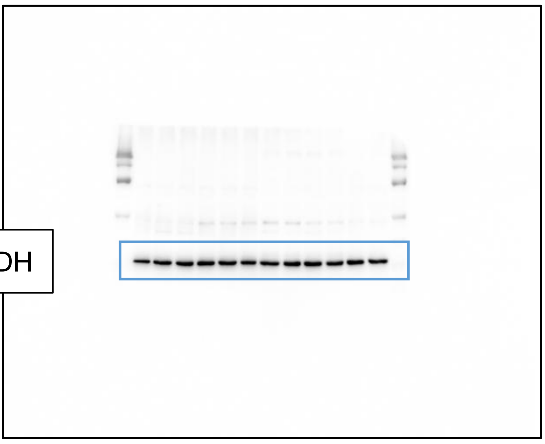

GAPDH

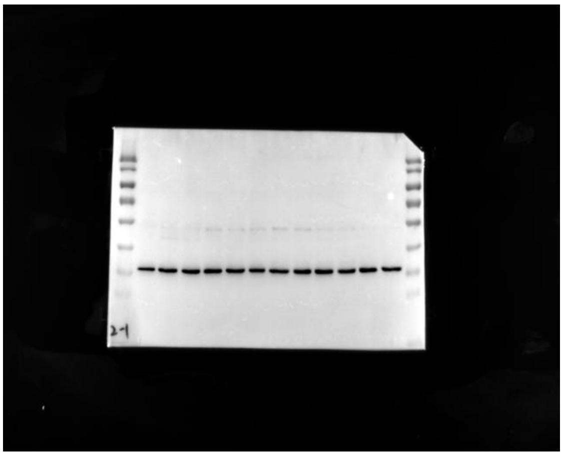

Figure 3b

The MARKER from top to bottom are sequentially 180, 130, 100, 70, 55, 40, 35, 25, 15 (ThermoFisher Scientific #26616).

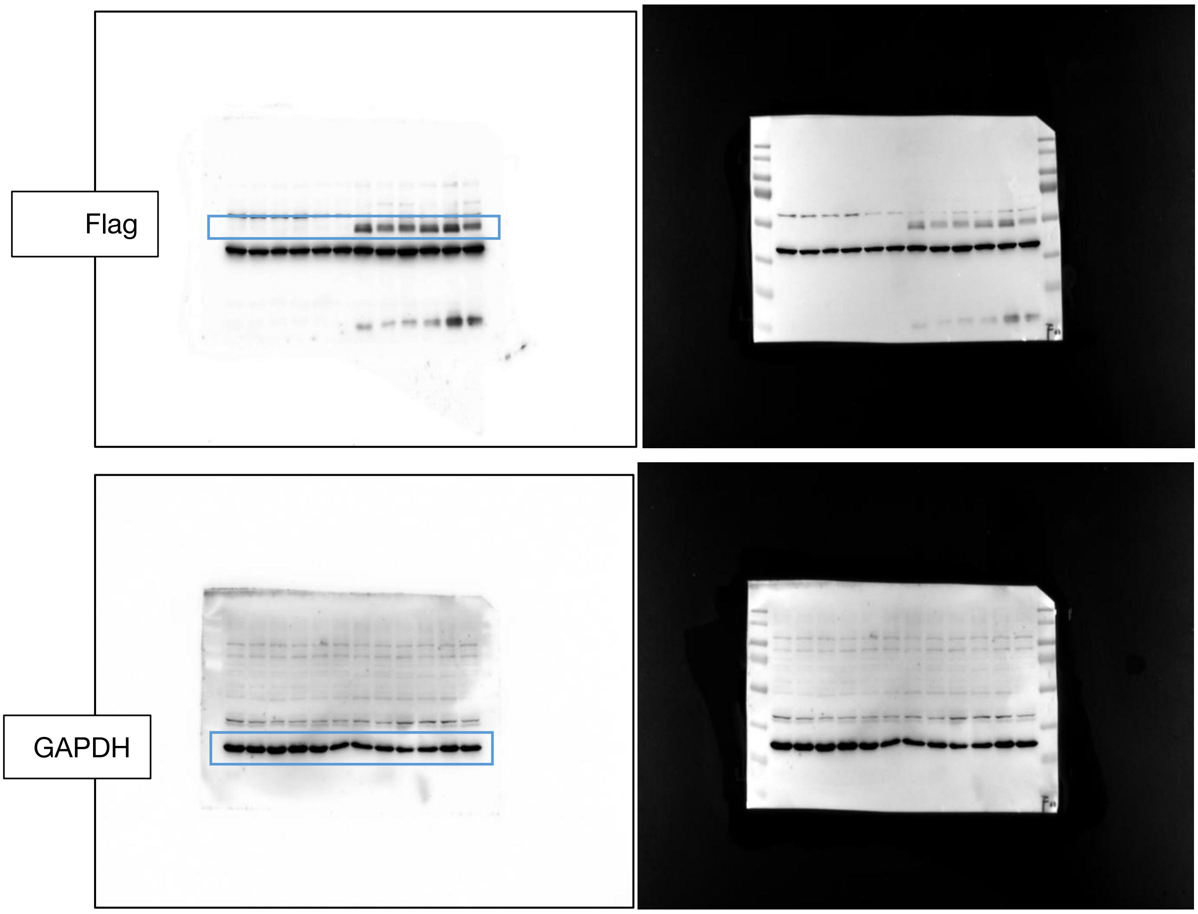

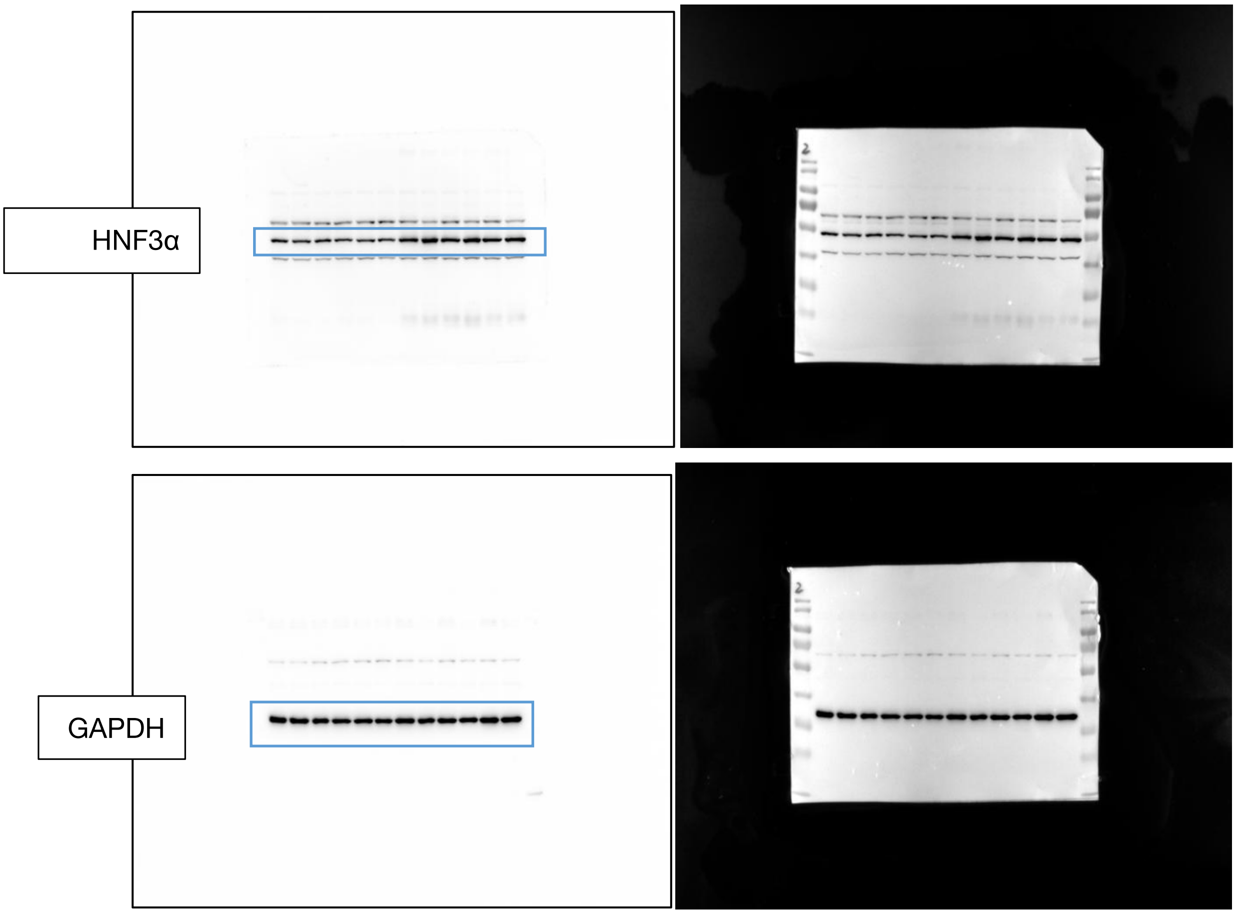
HNF3α
GAPDH

Figure 3g

The MARKER from top to bottom are sequentially 180, 130, 100, 70, 55, 40, 35, 25, 15 (ThermoFisher Scientific #26616).

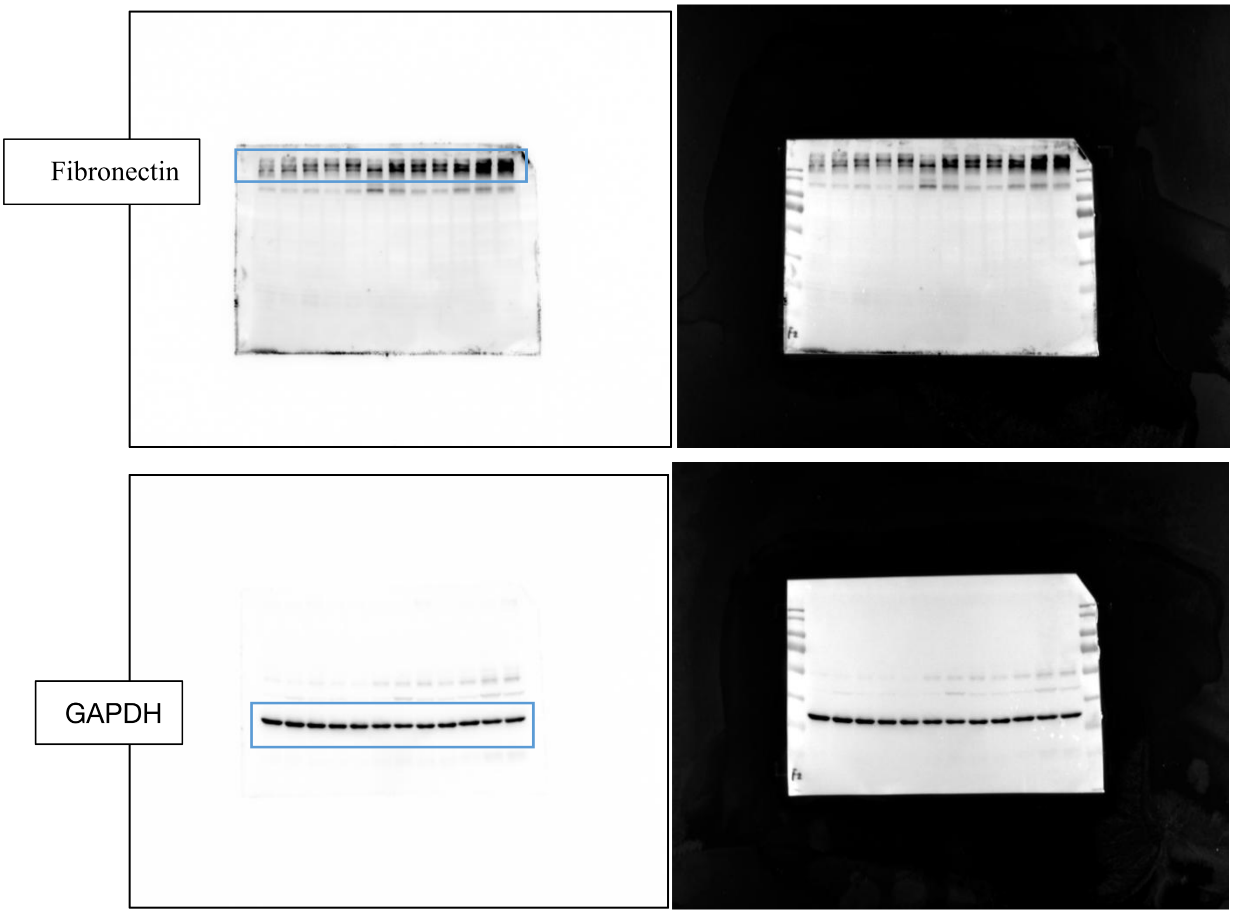

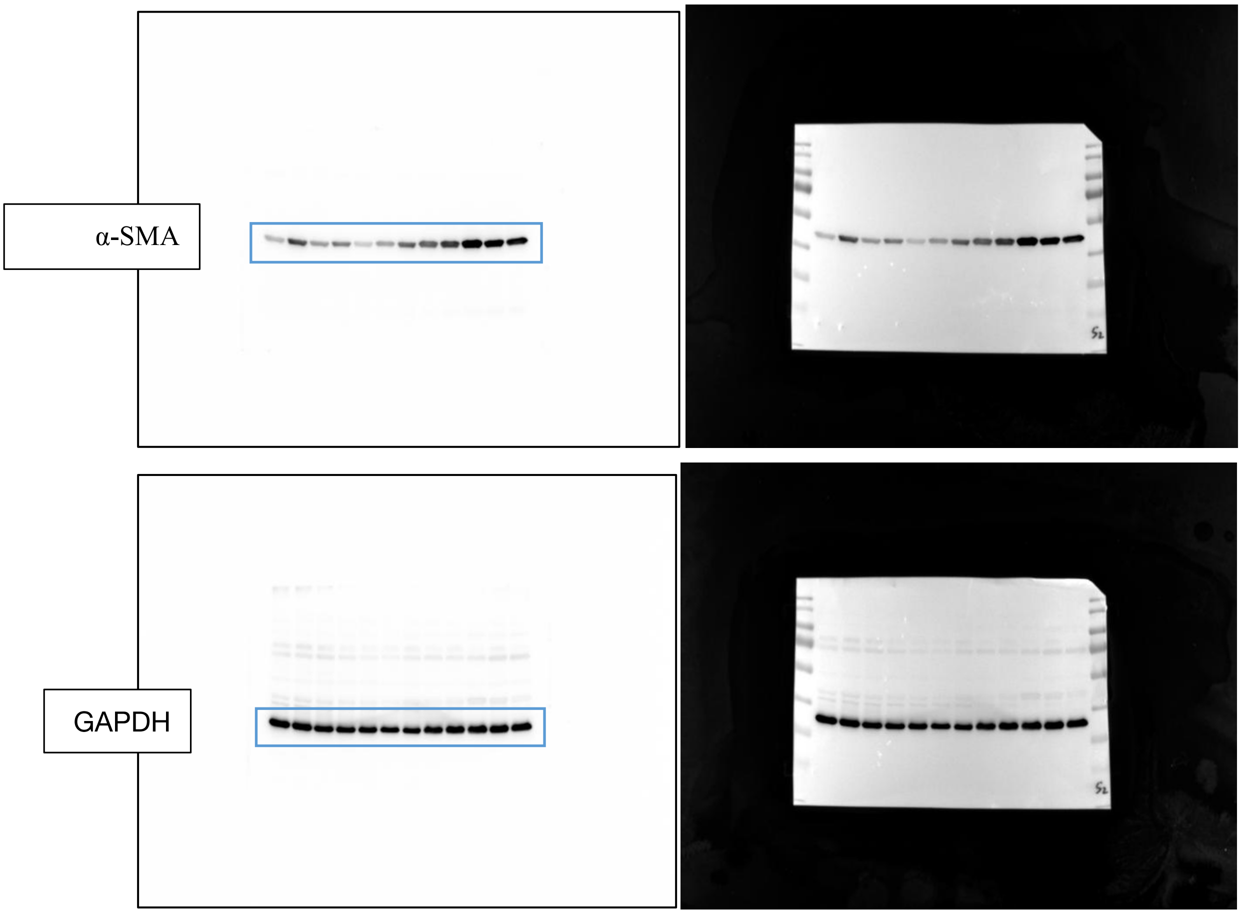
α-SMA
GAPDH

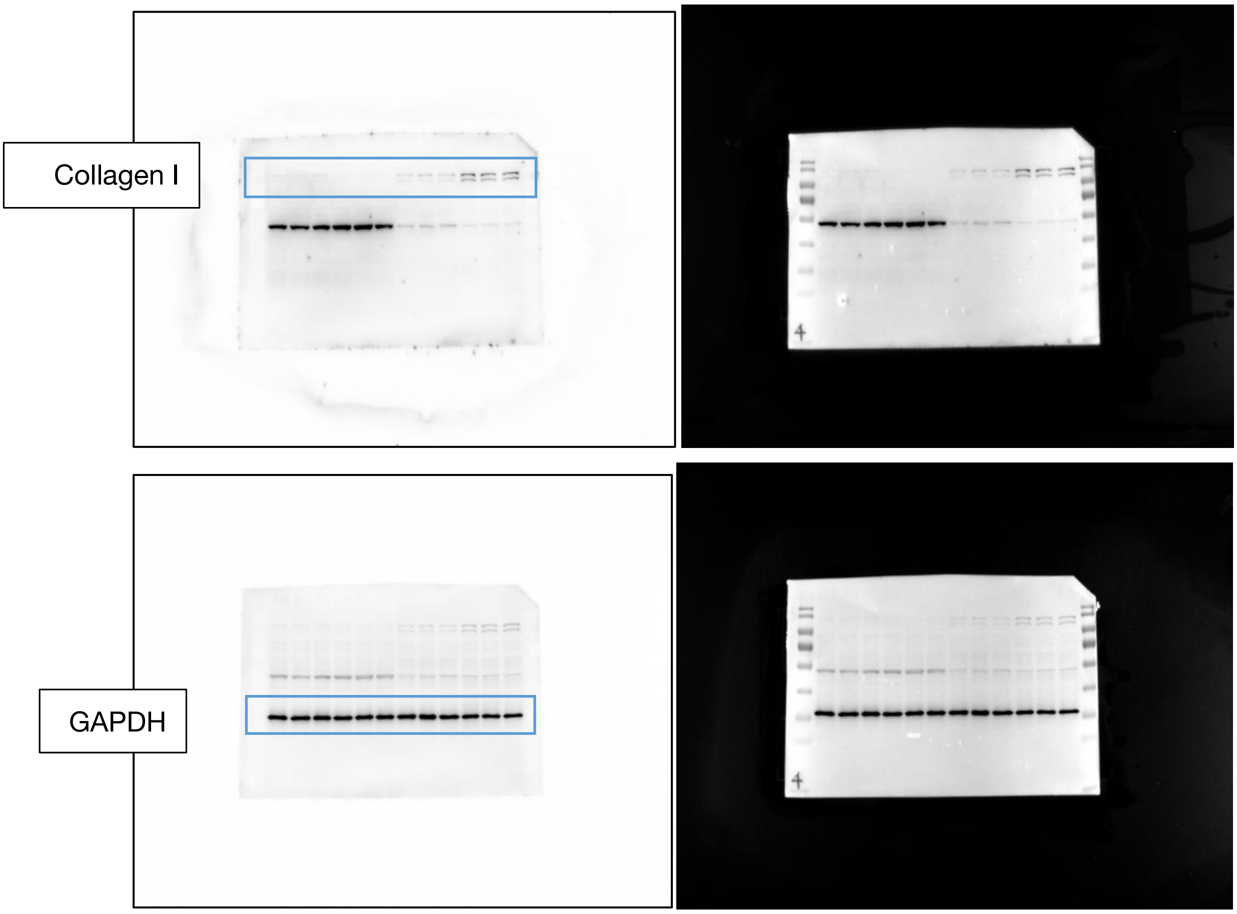
Collagen I
GAPDH

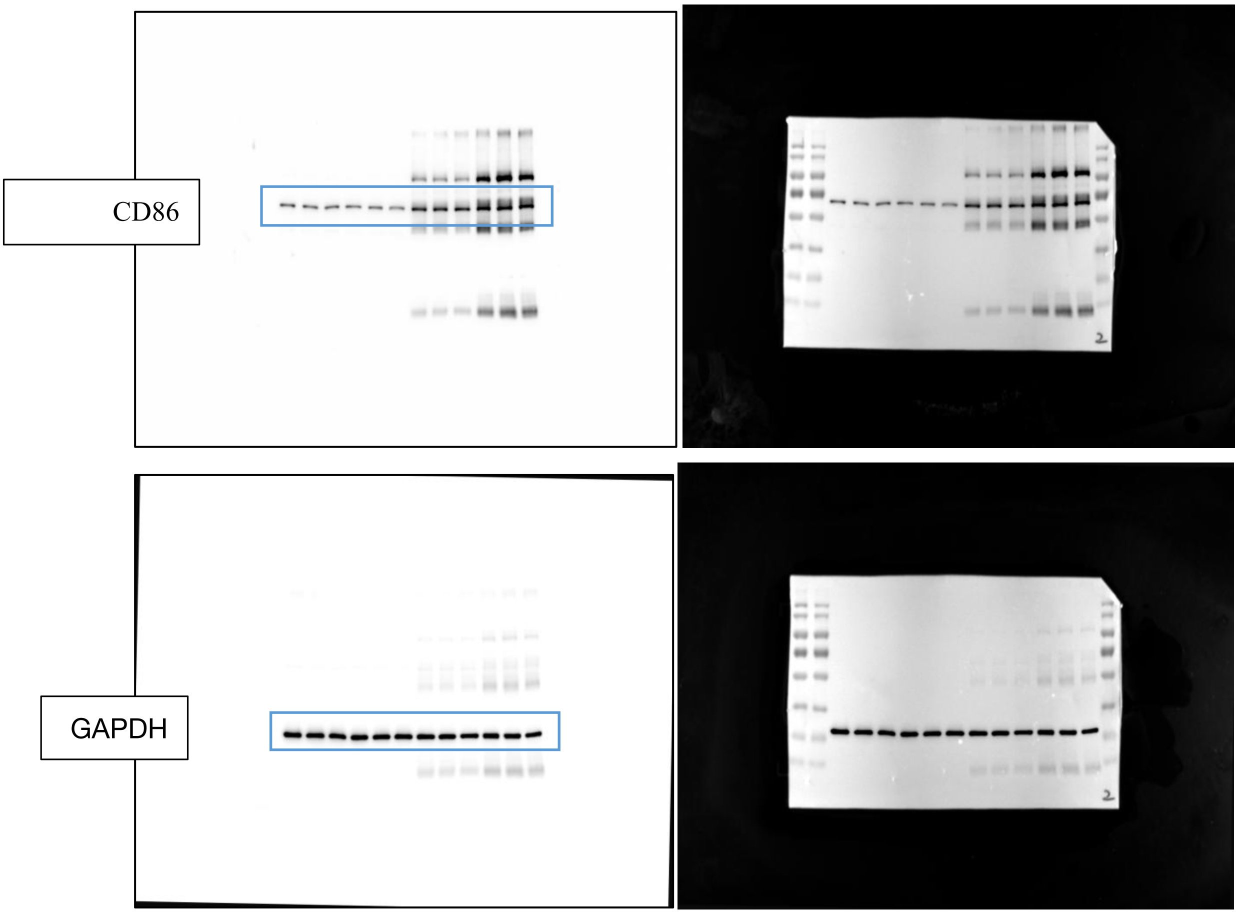
CD86
GAPDH

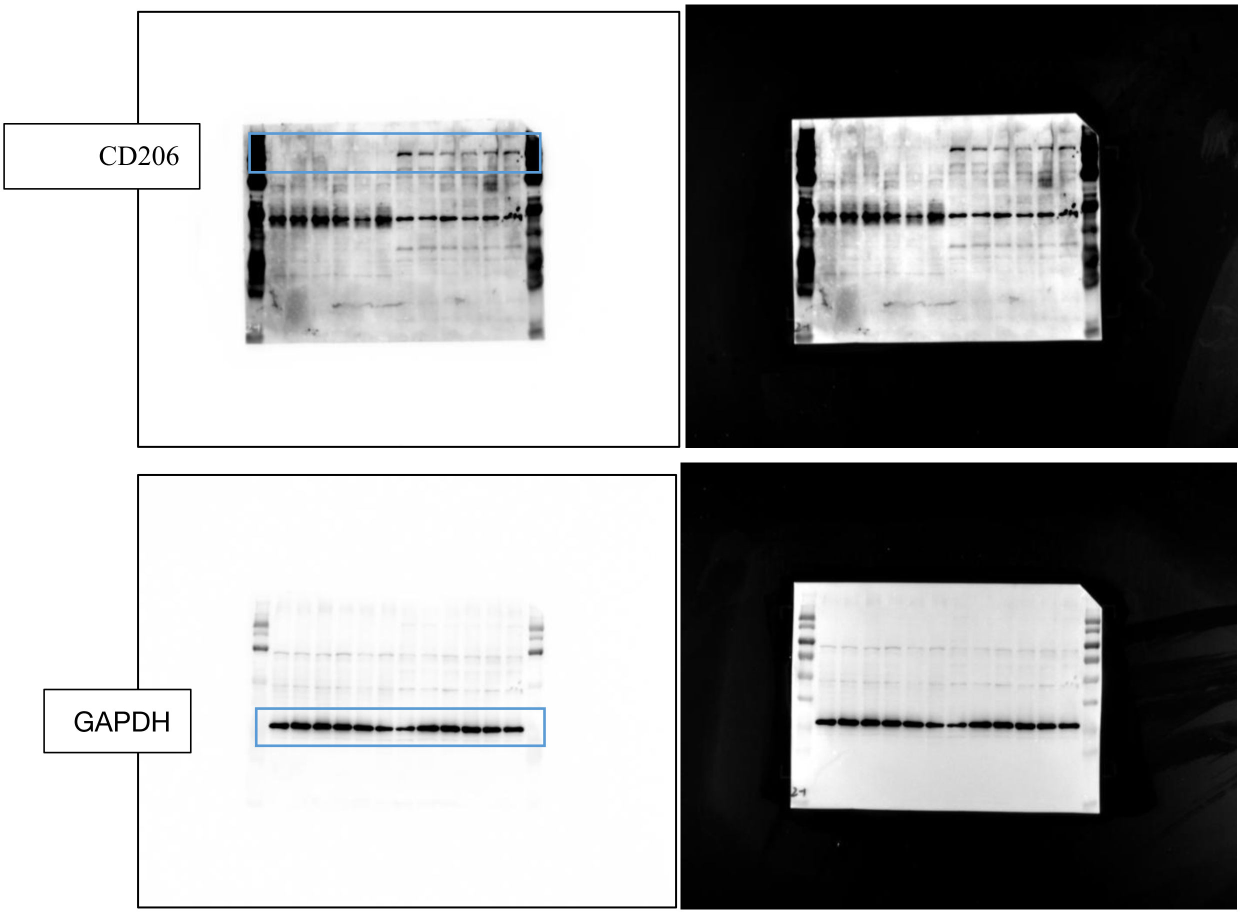
CD206
GAPDH

Figure 4a

The MARKER from top to bottom are sequentially 180, 130, 100, 70, 55, 40, 35, 25, 15 (ThermoFisher Scientific #26616).

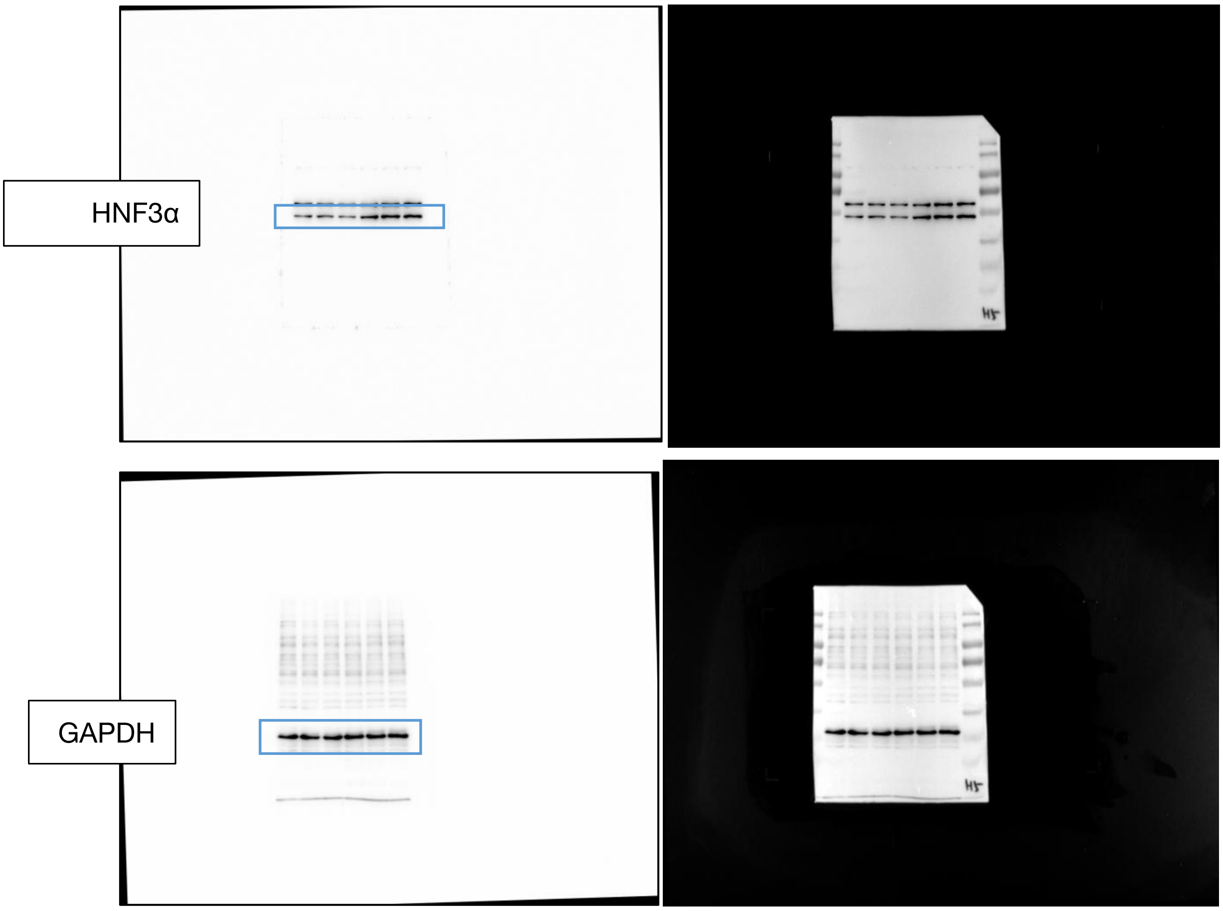

Figure 4c

The MARKER from top to bottom are sequentially 180, 130, 100, 70, 55, 40, 35, 25, 15 (ThermoFisher Scientific #26616).

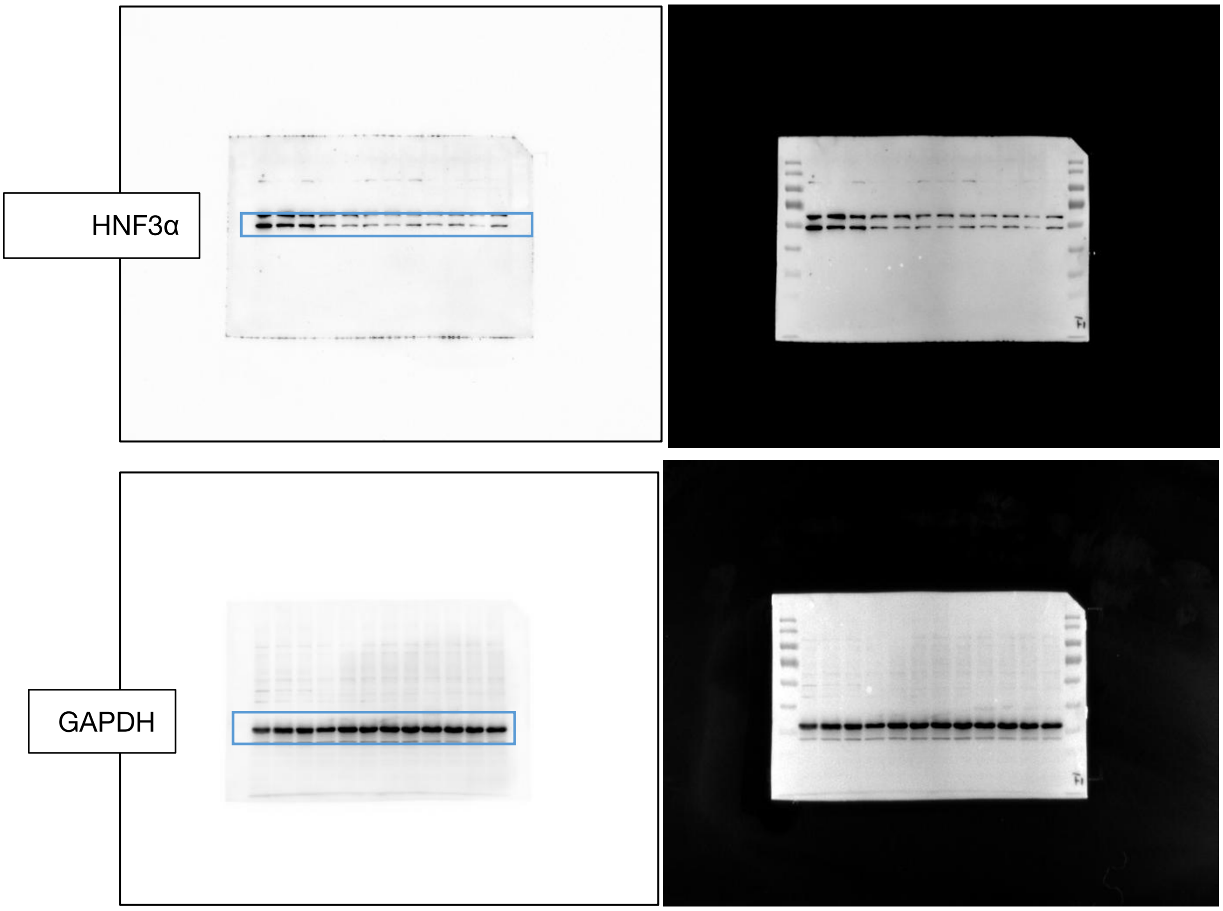

Figure 4e

The MARKER from top to bottom are sequentially 180, 130, 100, 70, 55, 40, 35, 25, 15 (ThermoFisher Scientific #26616).

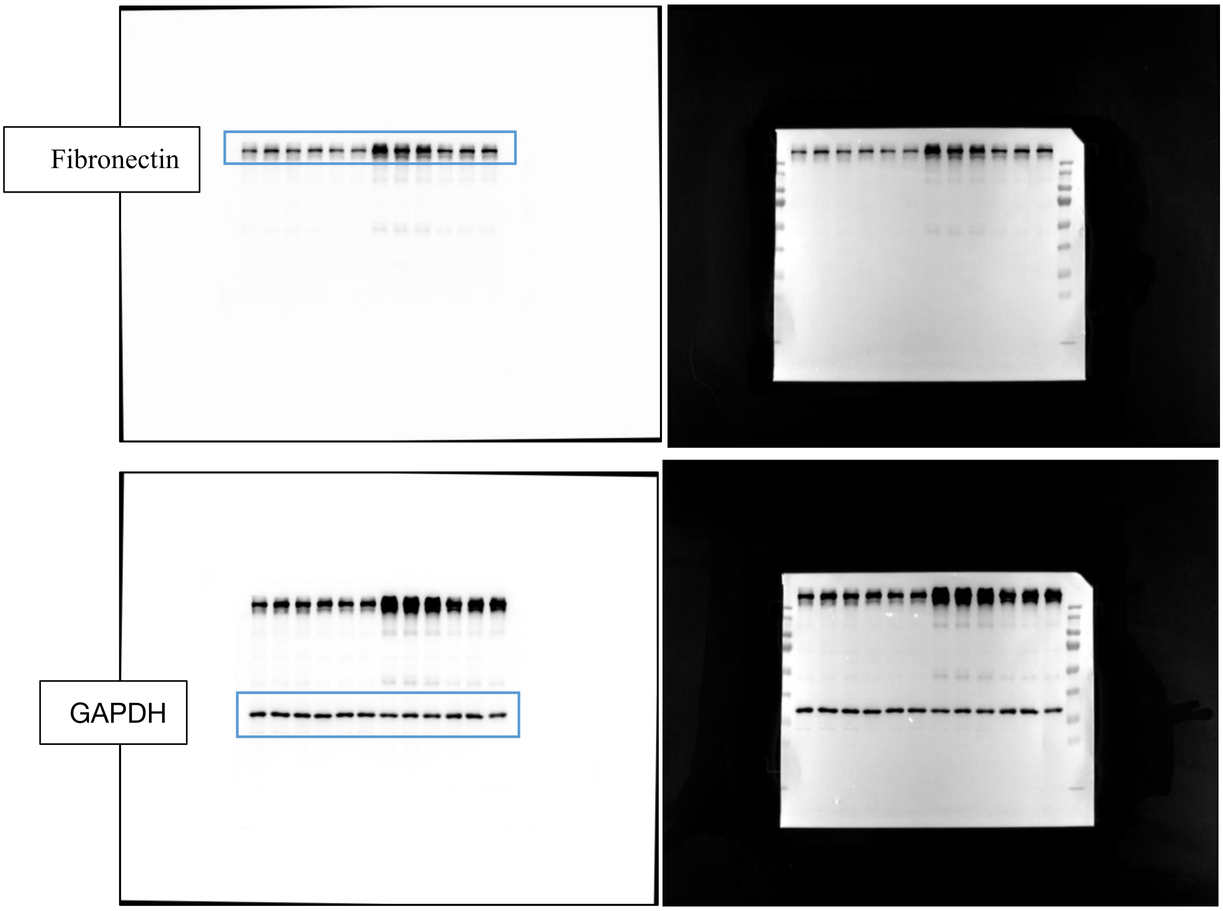

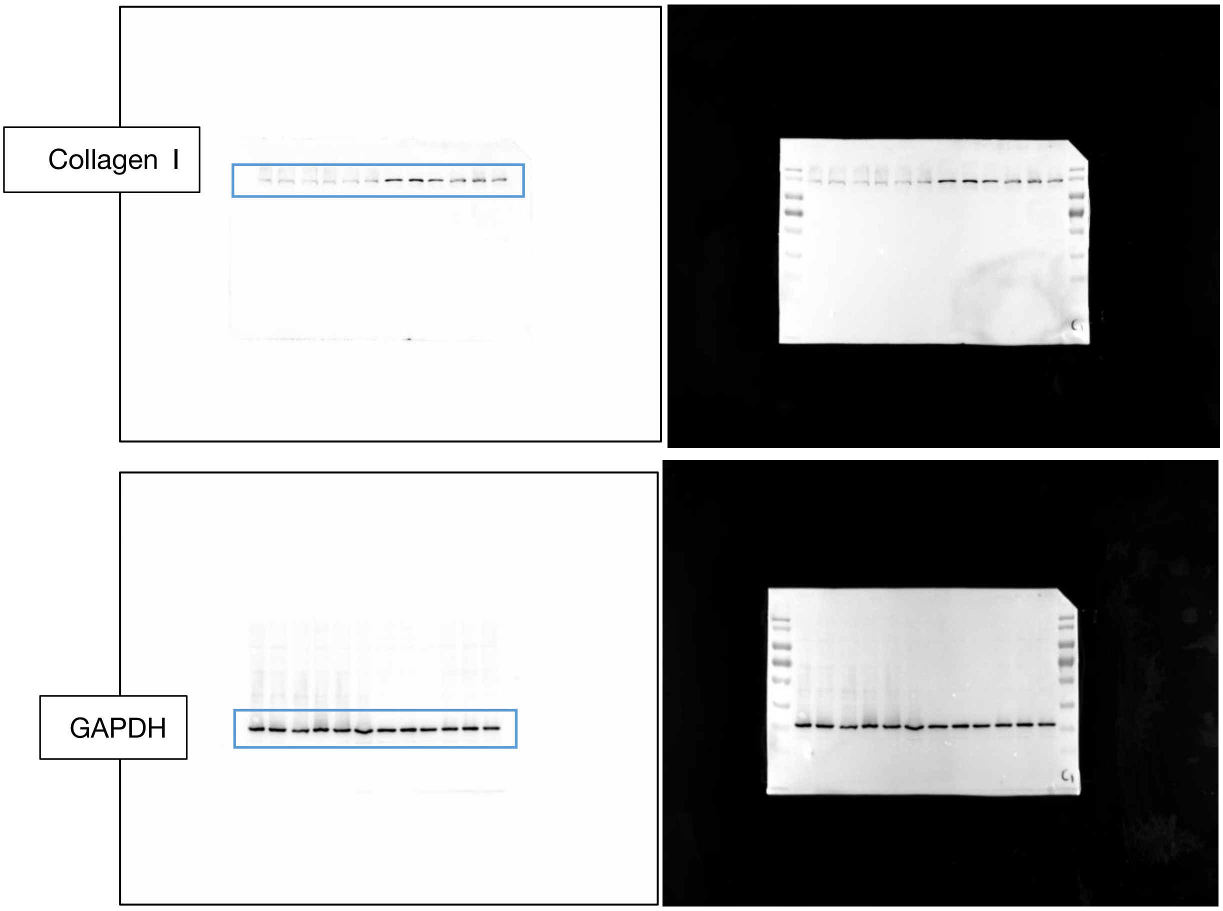
Collagen I
GAPDH

Figure 4h

The MARKER from top to bottom are sequentially 180, 130, 100, 70, 55, 40, 35, 25, 15 (ThermoFisher Scientific #26616).

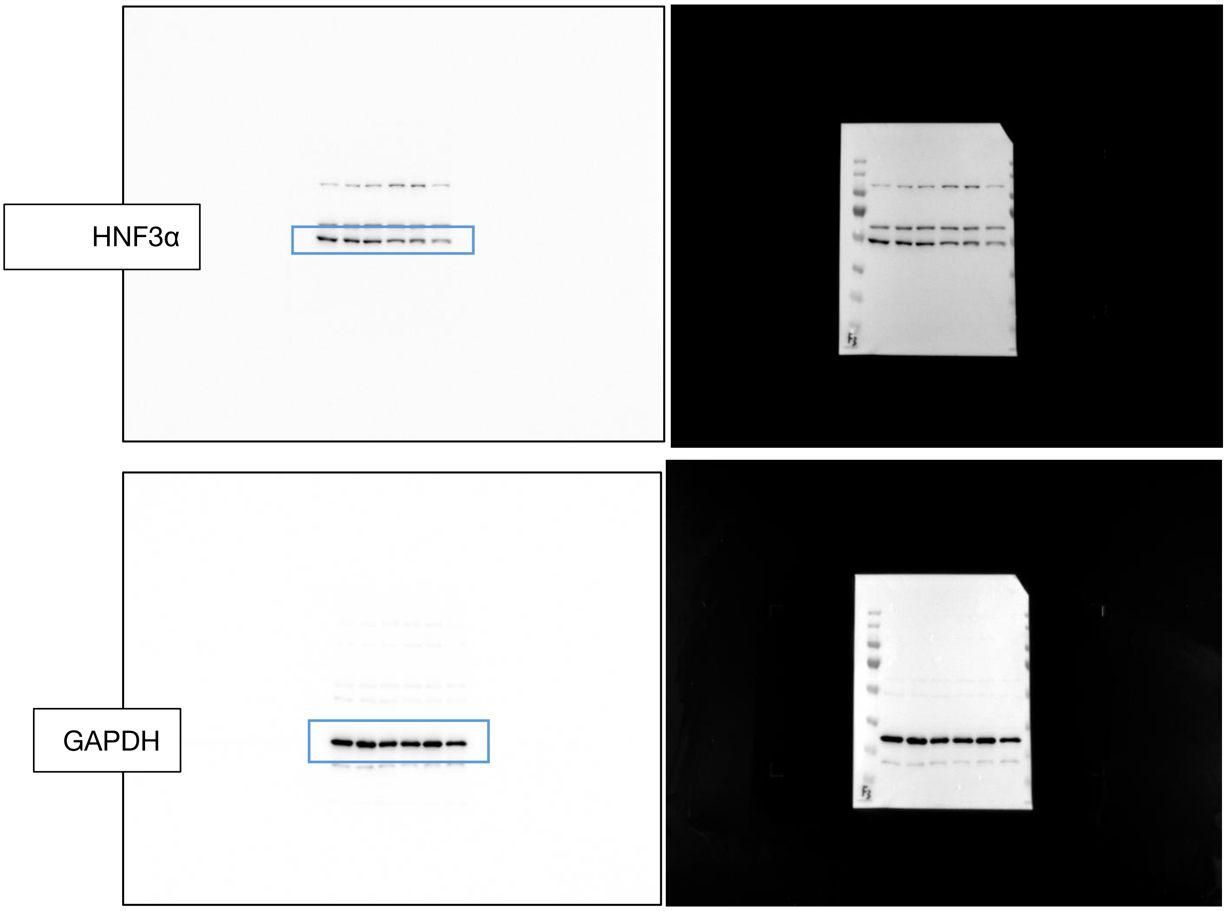

Figure 4j

The MARKER from top to bottom are sequentially 180, 130, 100, 70, 55, 40, 35, 25, 15 (ThermoFisher Scientific #26616).

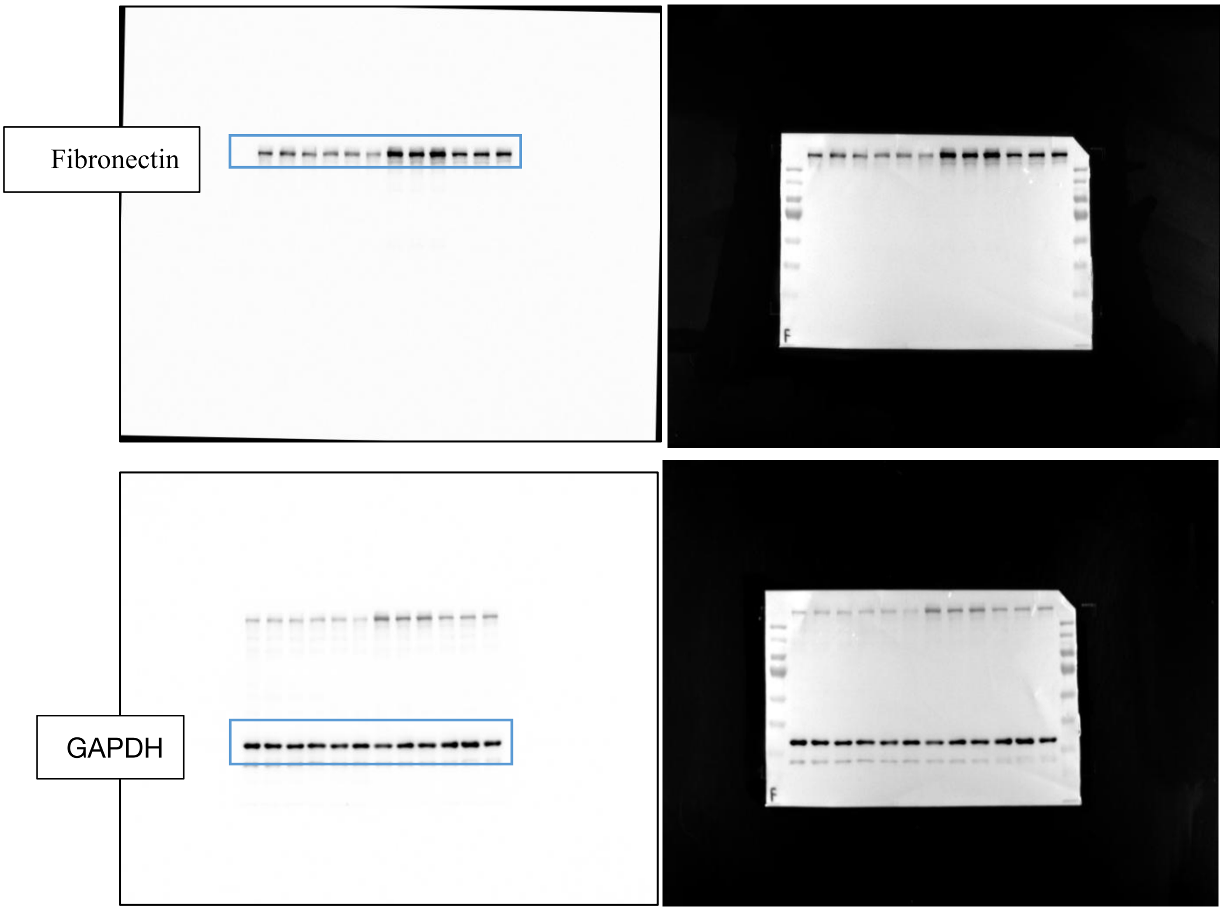

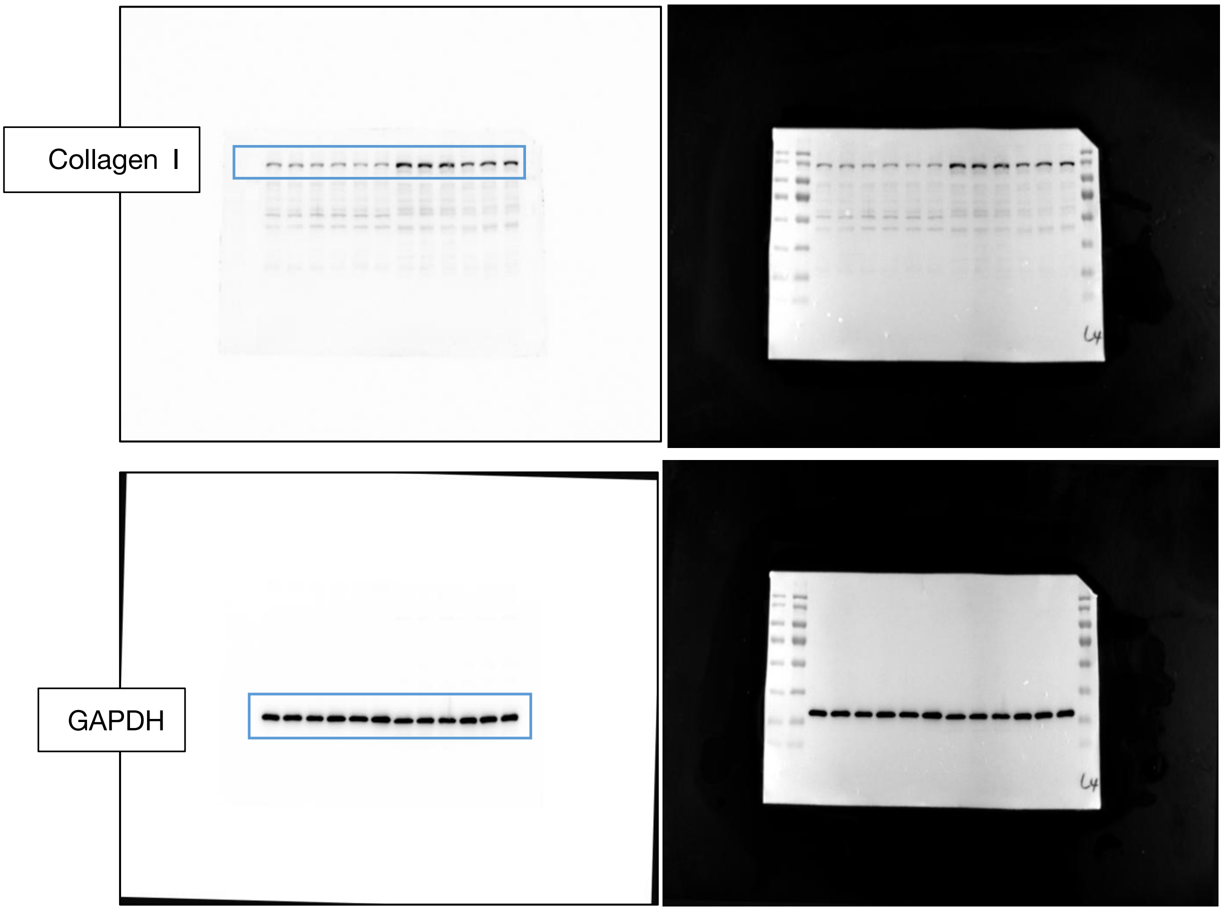
Collagen I
GAPDH

Figure 4l

The MARKER from top to bottom are sequentially 180, 130, 100, 70, 55, 40, 35, 25, 15 (ThermoFisher Scientific #26616).

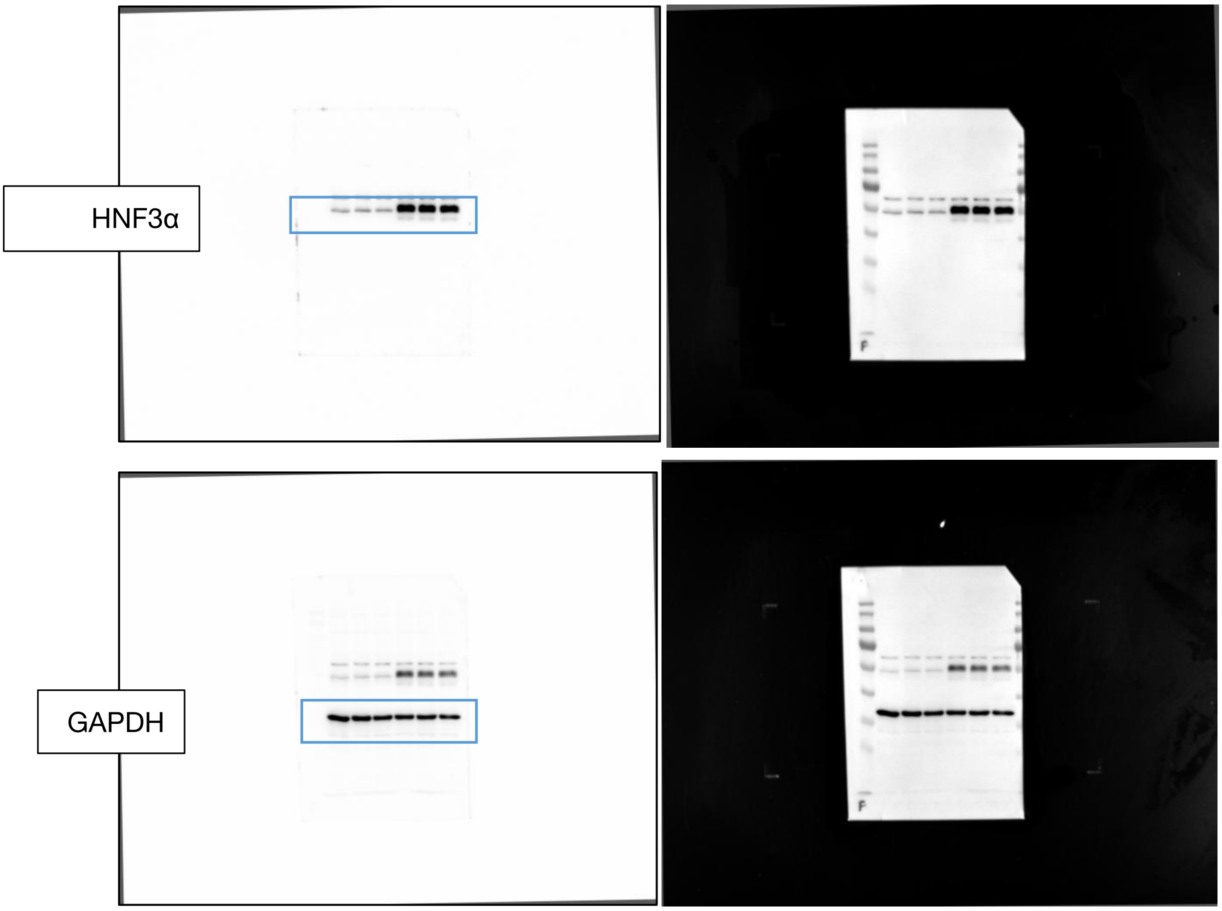

Figure 4n

The MARKER from top to bottom are sequentially 180, 130, 100, 70, 55, 40, 35, 25, 15 (ThermoFisher Scientific #26616).

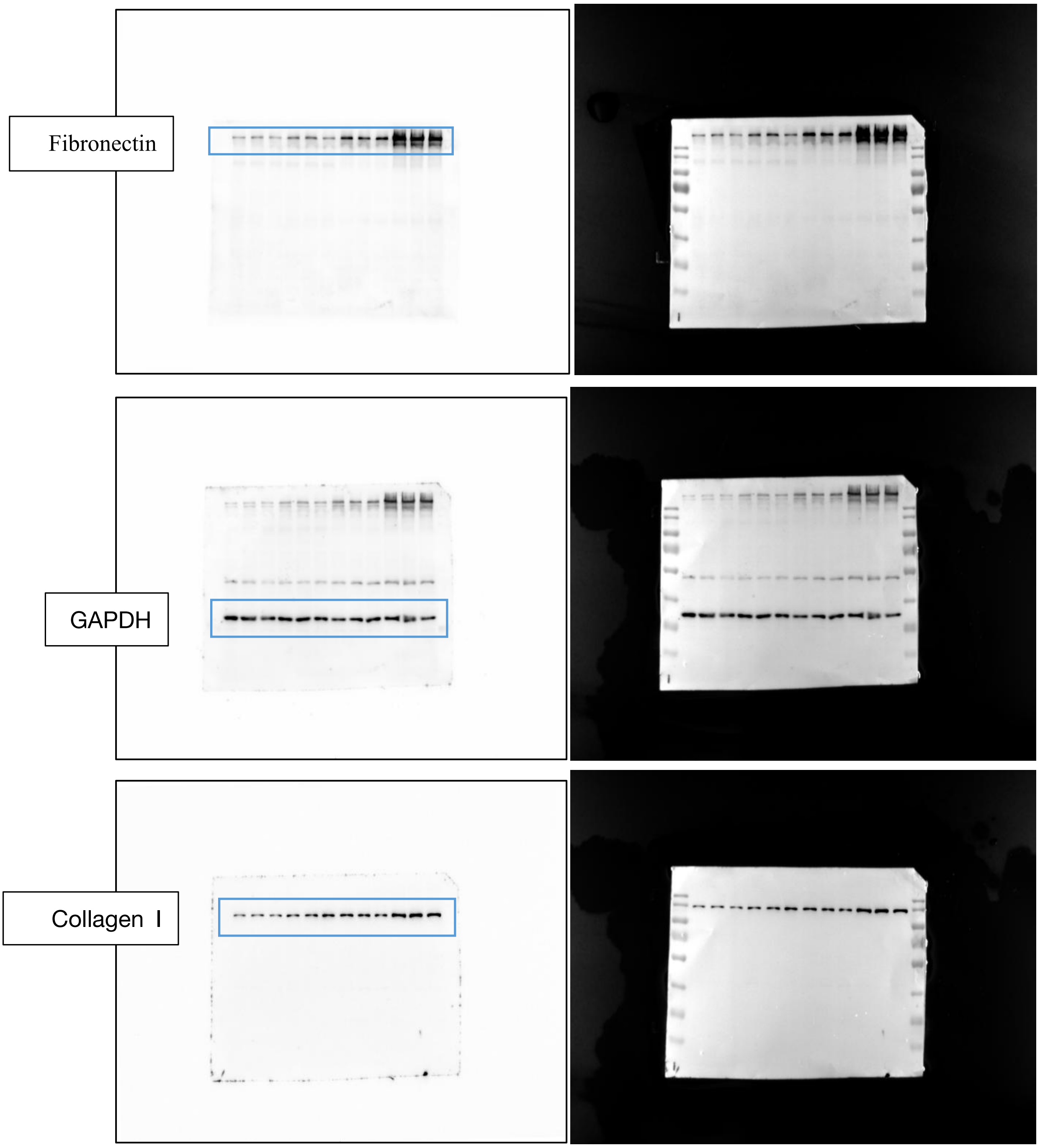

Figure 4p

The MARKER from top to bottom are sequentially 180, 130, 100, 70, 55, 40, 35, 25, 15 (ThermoFisher Scientific #26616).

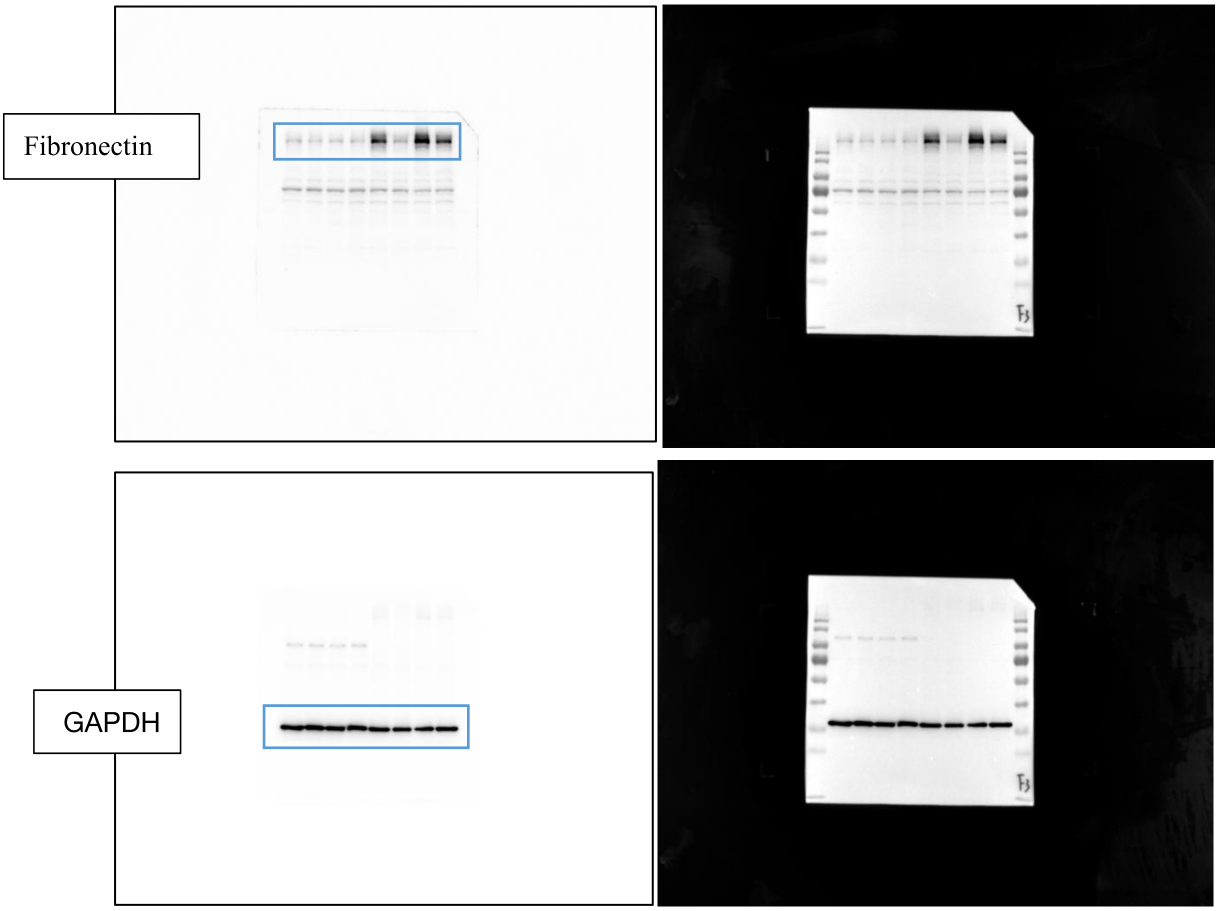

Figure 5g

The MARKER from top to bottom are sequentially 180, 130, 100, 70, 55, 40, 35, 25, 15 (ThermoFisher Scientific #26616).

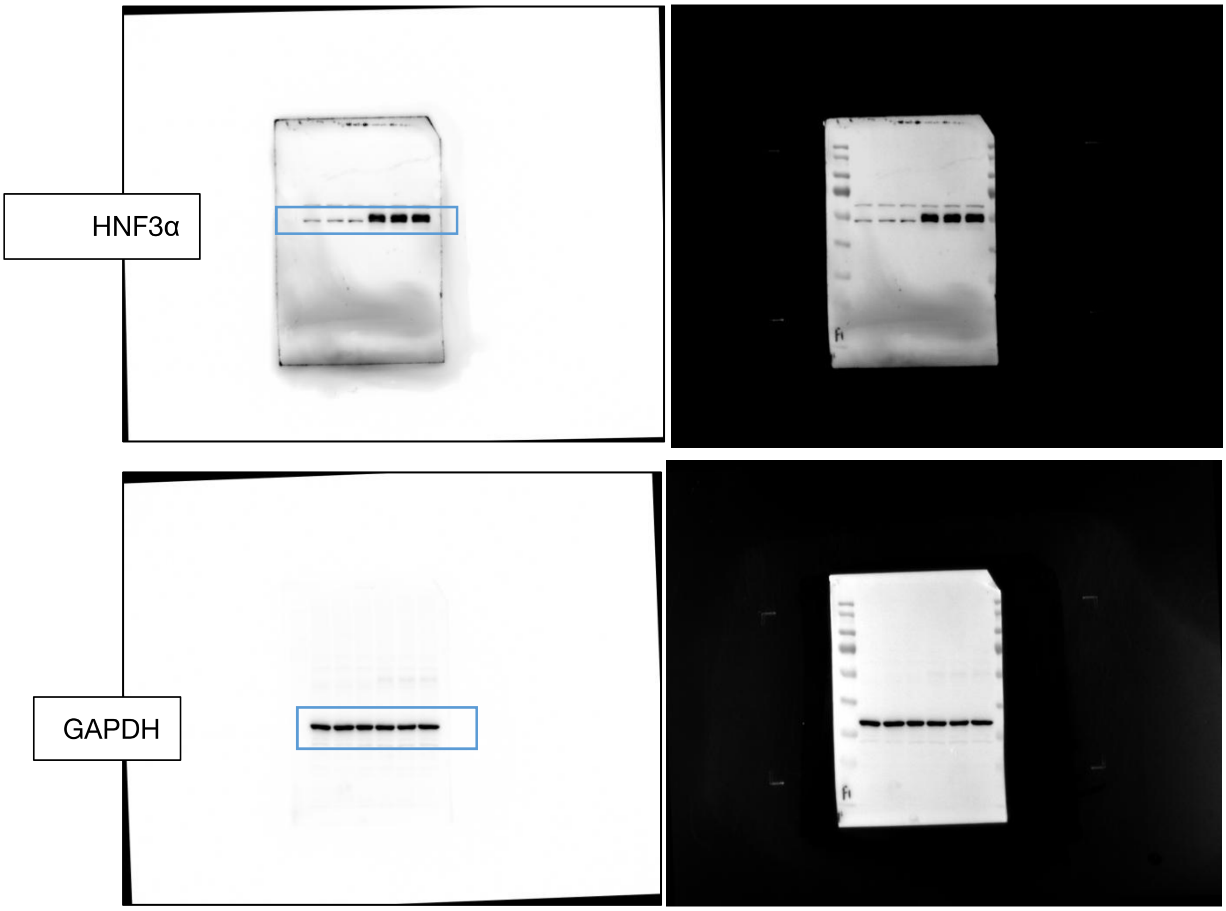

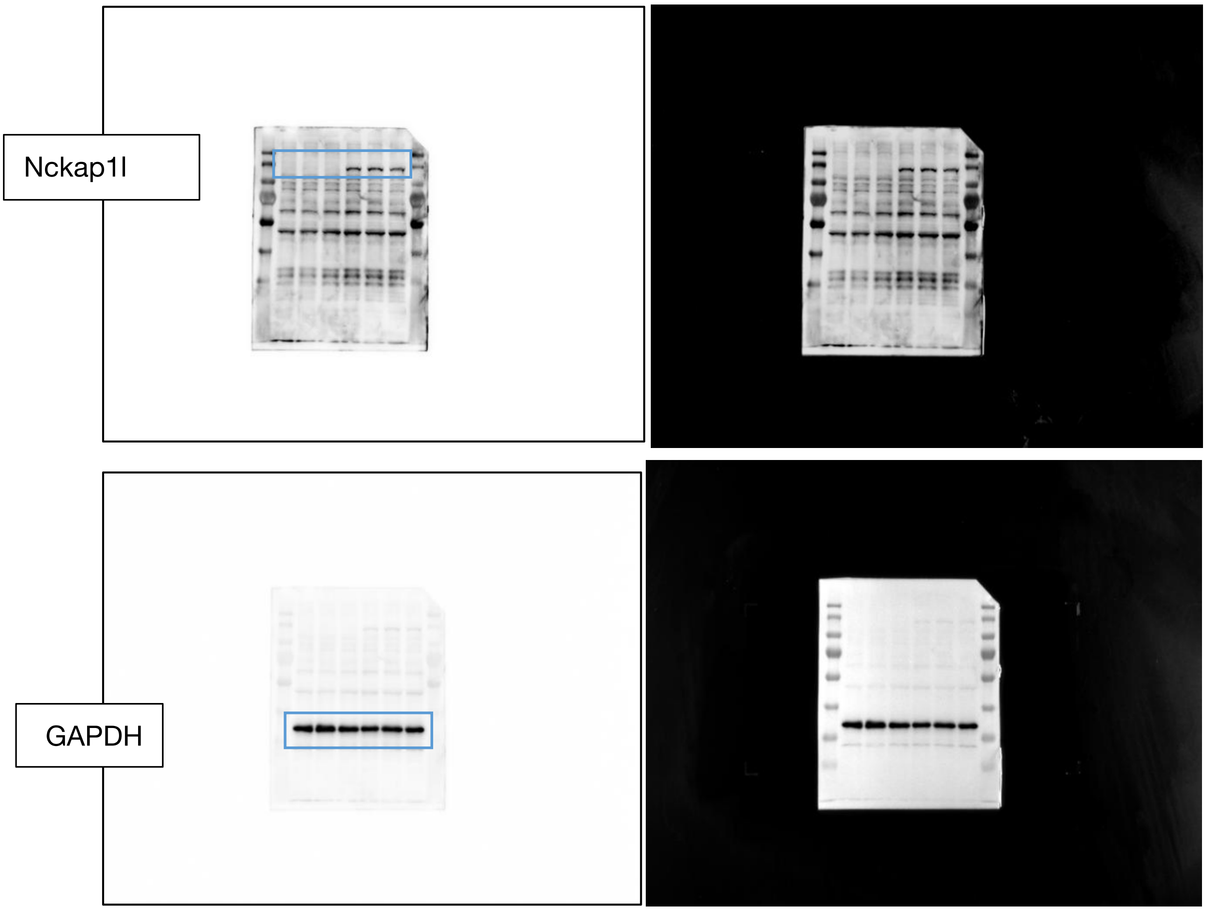
Nckap1l
GAPDH

Figure 5i

The MARKER from top to bottom are sequentially 180, 130, 100, 70, 55, 40, 35, 25, 15 (ThermoFisher Scientific #26616).

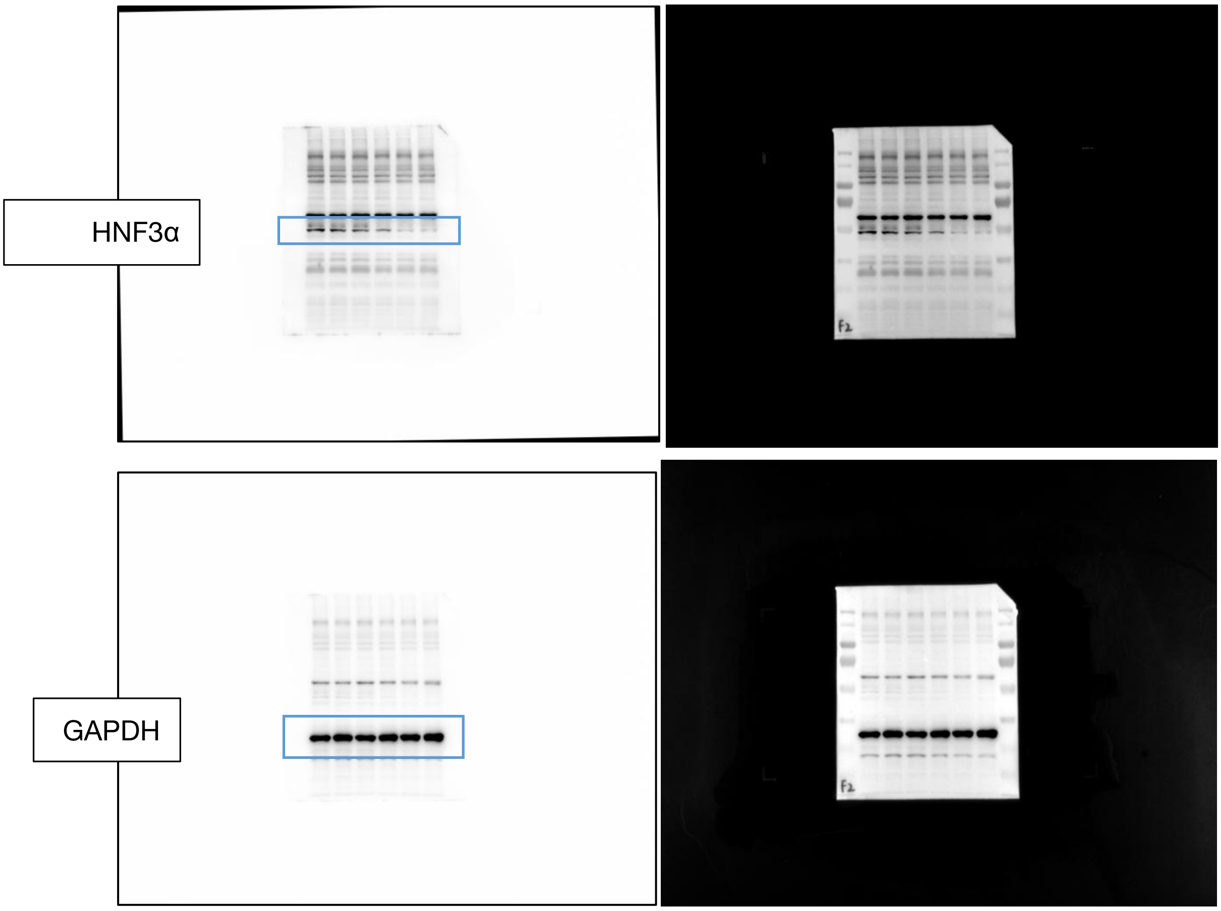

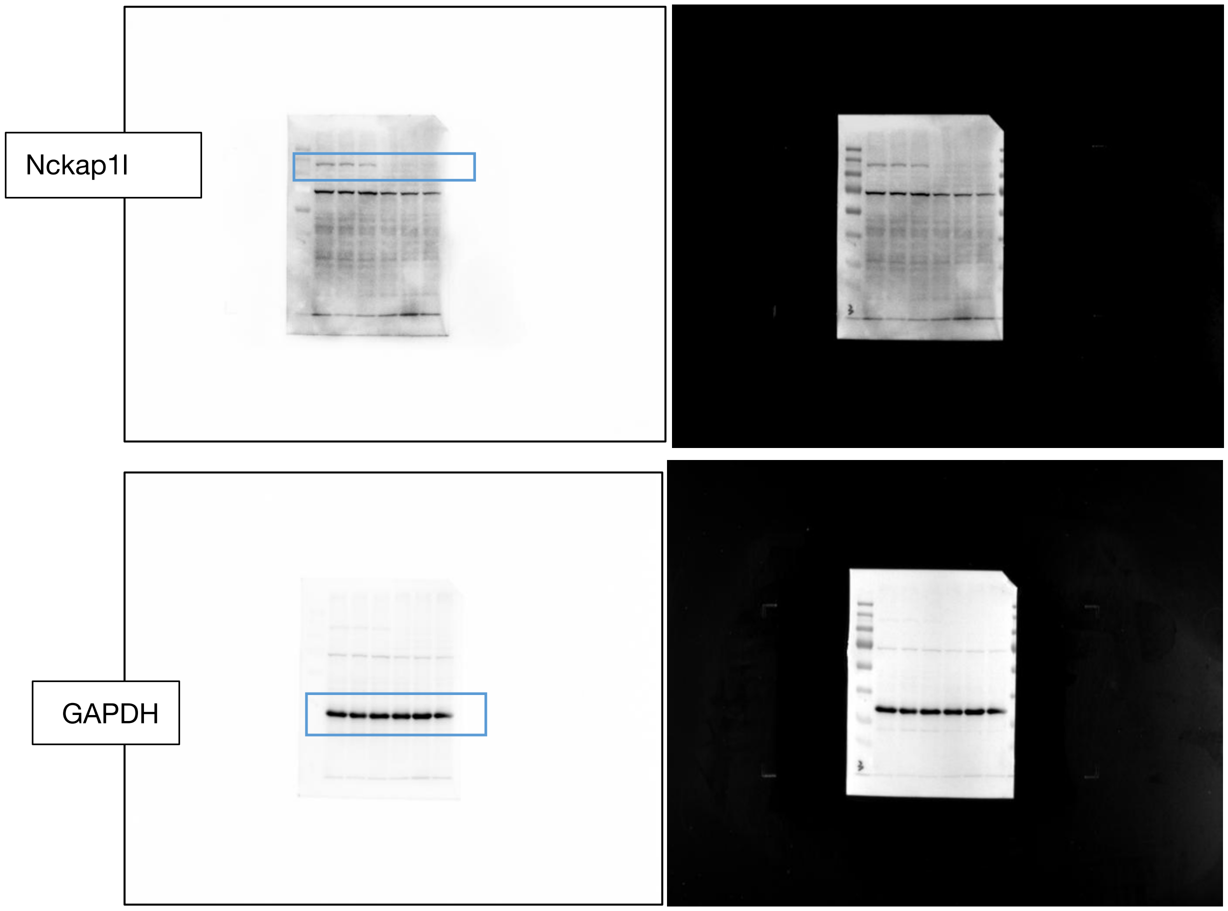
Nckap1l
GAPDH

Figure 5k

The MARKER from top to bottom are sequentially 180, 130, 100, 70, 55, 40, 35, 25, 15 (ThermoFisher Scientific #26616).

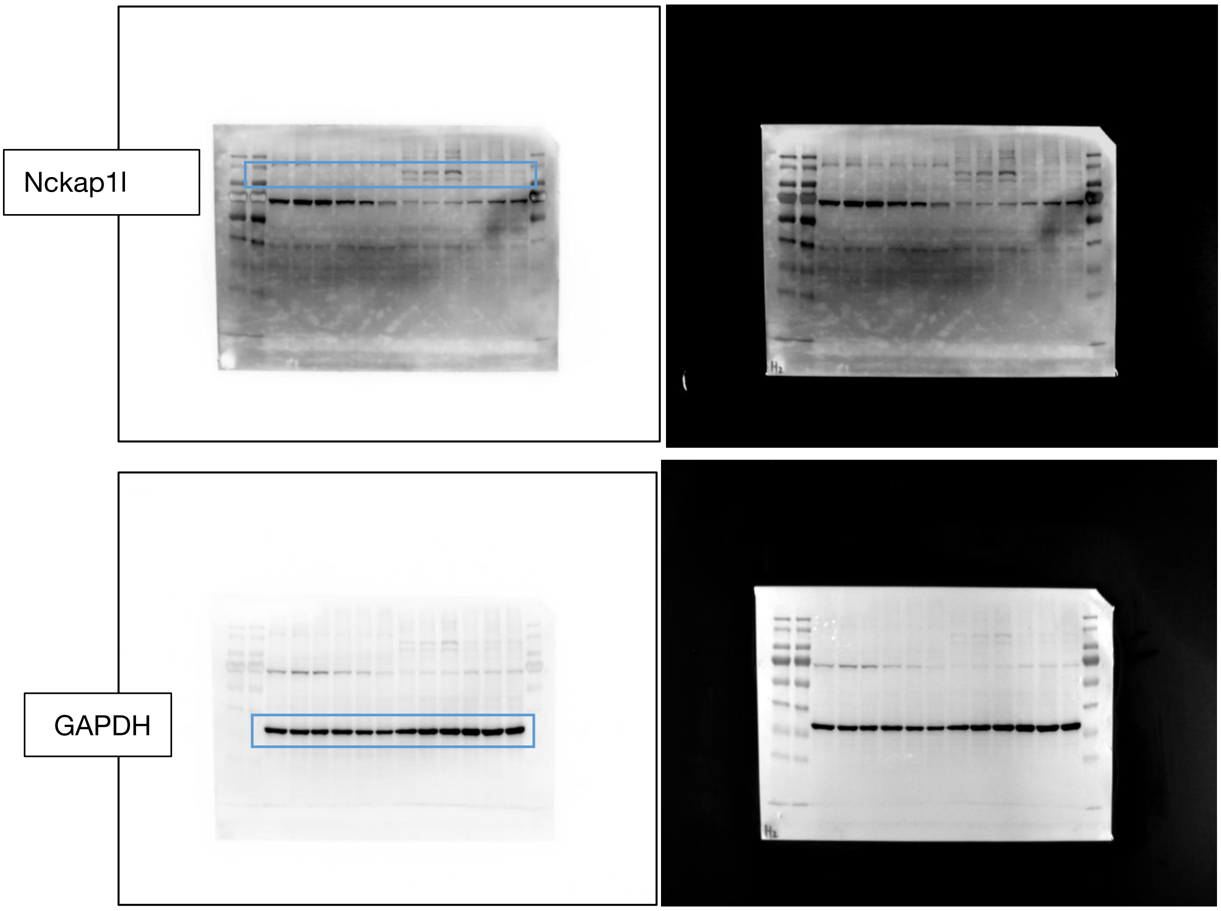

Figure 6c

The MARKER from top to bottom are sequentially 180, 130, 100, 70, 55, 40, 35, 25, 15 (ThermoFisher Scientific #26616).

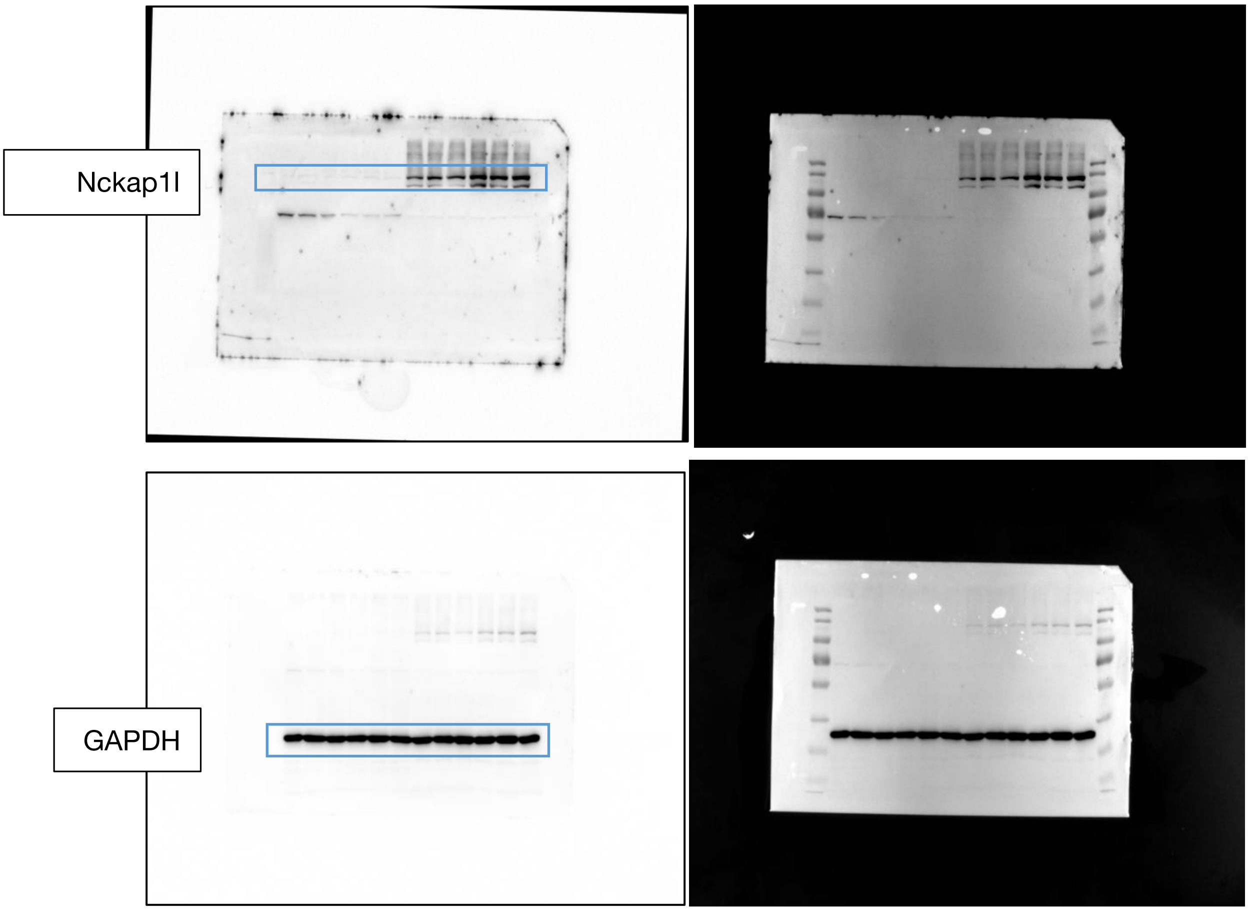

Figure 6g

The MARKER from top to bottom are sequentially 180, 130, 100, 70, 55, 40, 35, 25, 15 (ThermoFisher Scientific #26616).

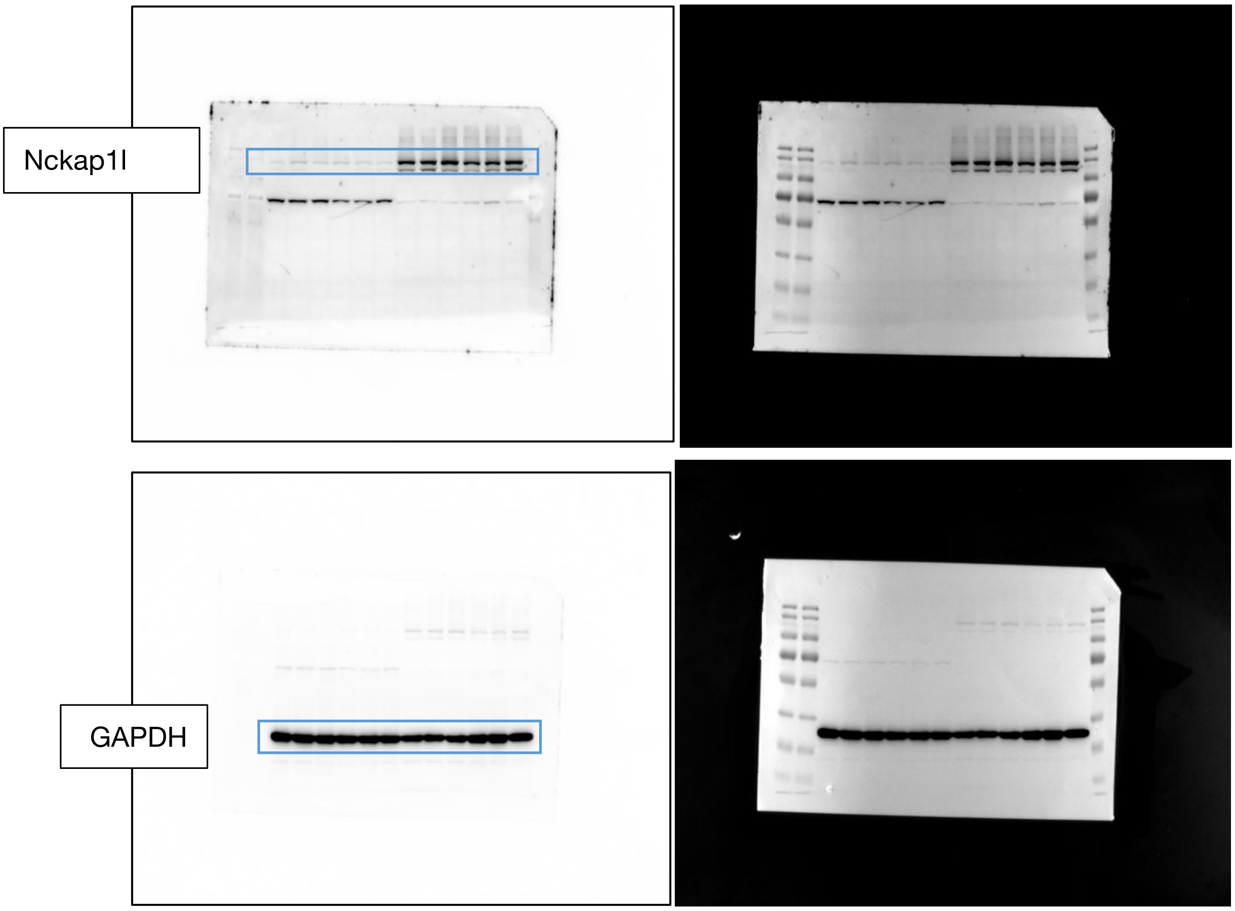

Figure 7c

The MARKER from top to bottom are sequentially 180, 130, 100, 70, 55, 40, 35, 25, 15 (ThermoFisher Scientific #26616).

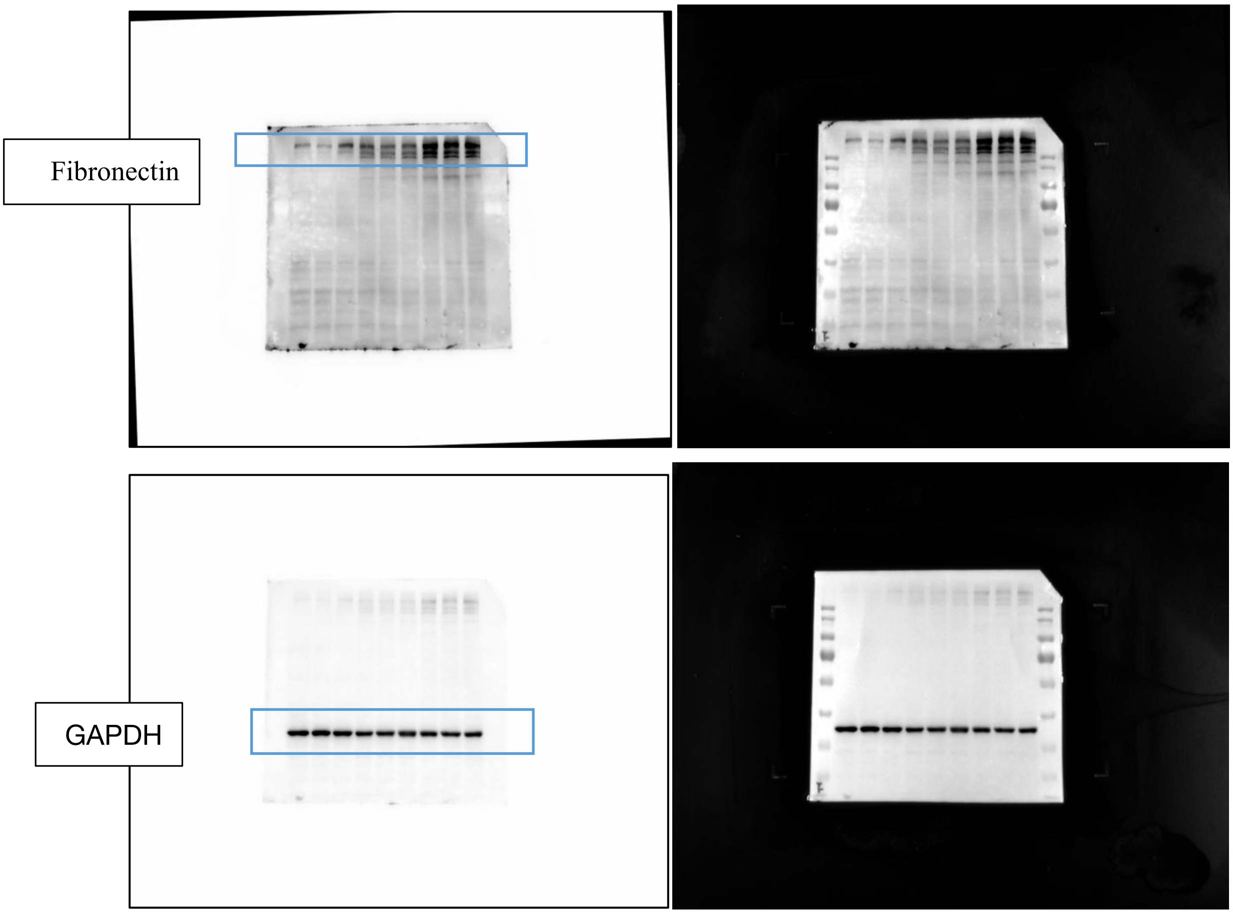

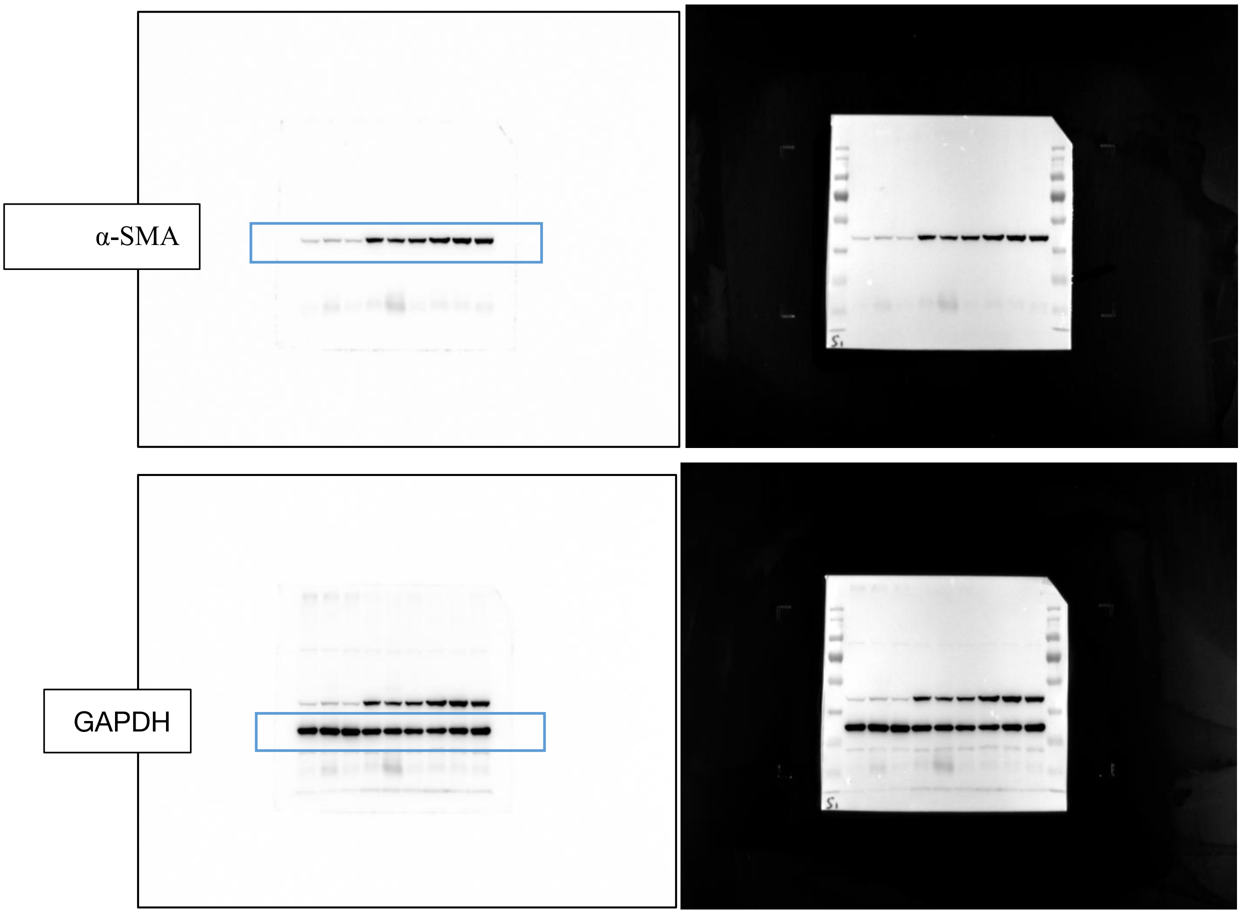
α-SMA
GAPDH

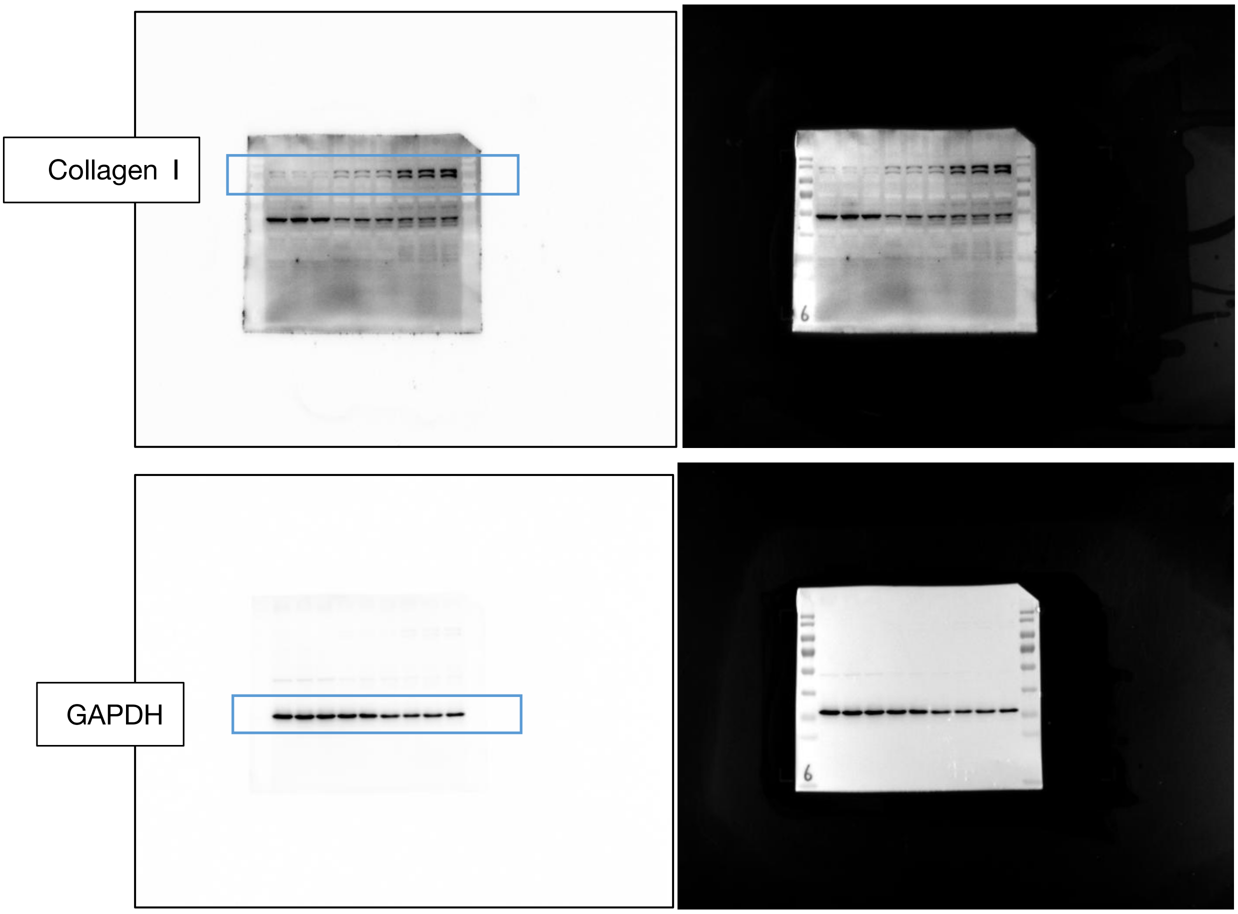
Collagen I
GAPDH

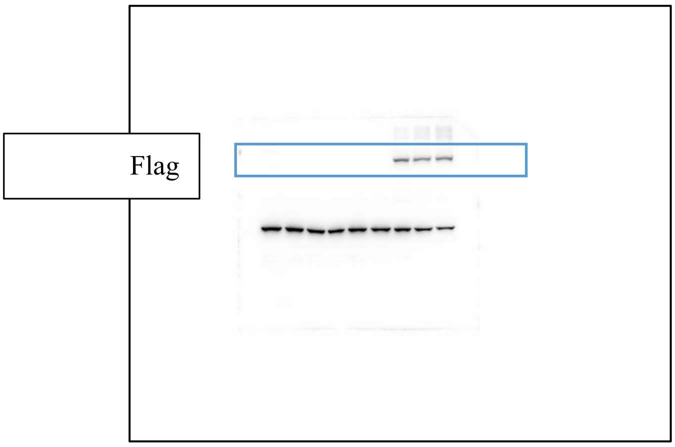
Flag

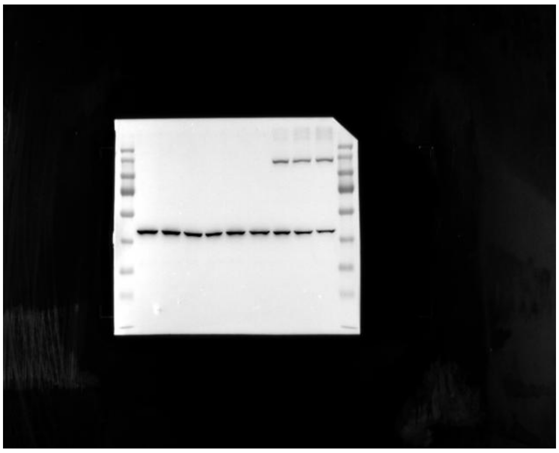

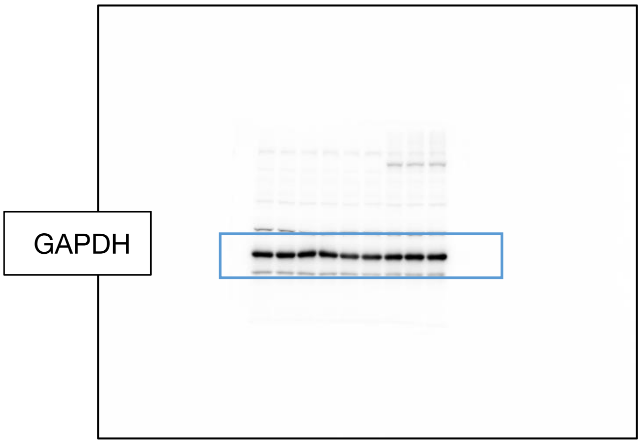
GAPDH

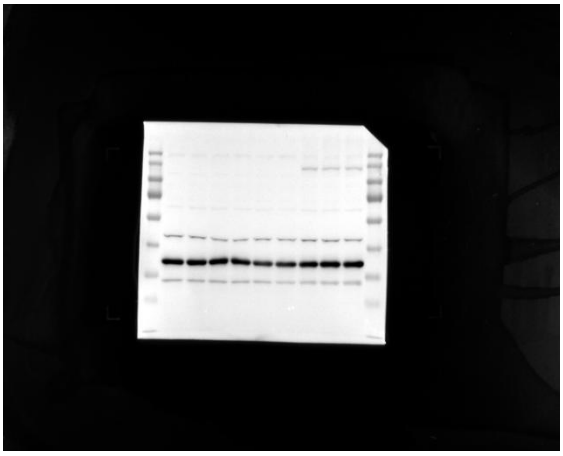

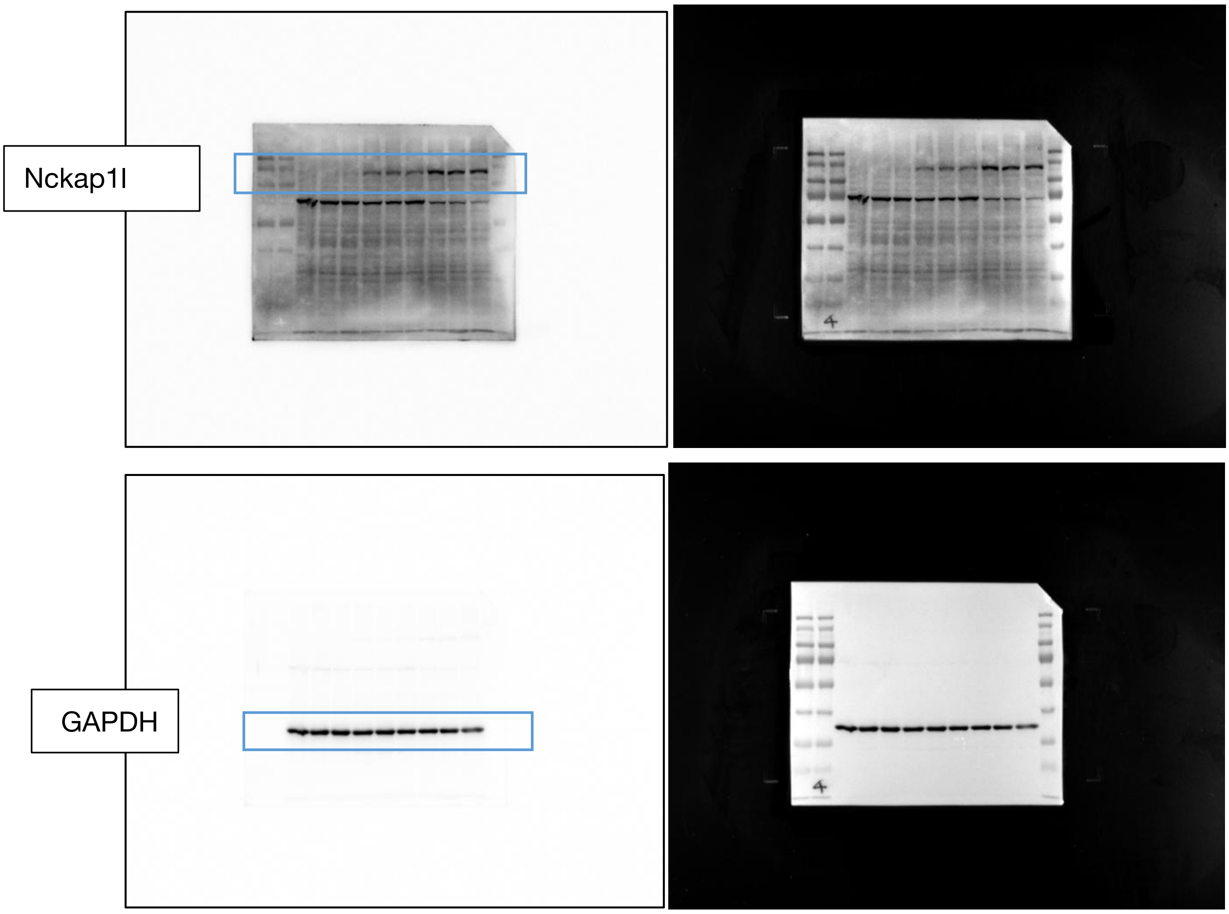

Nckap1l
GAPDH

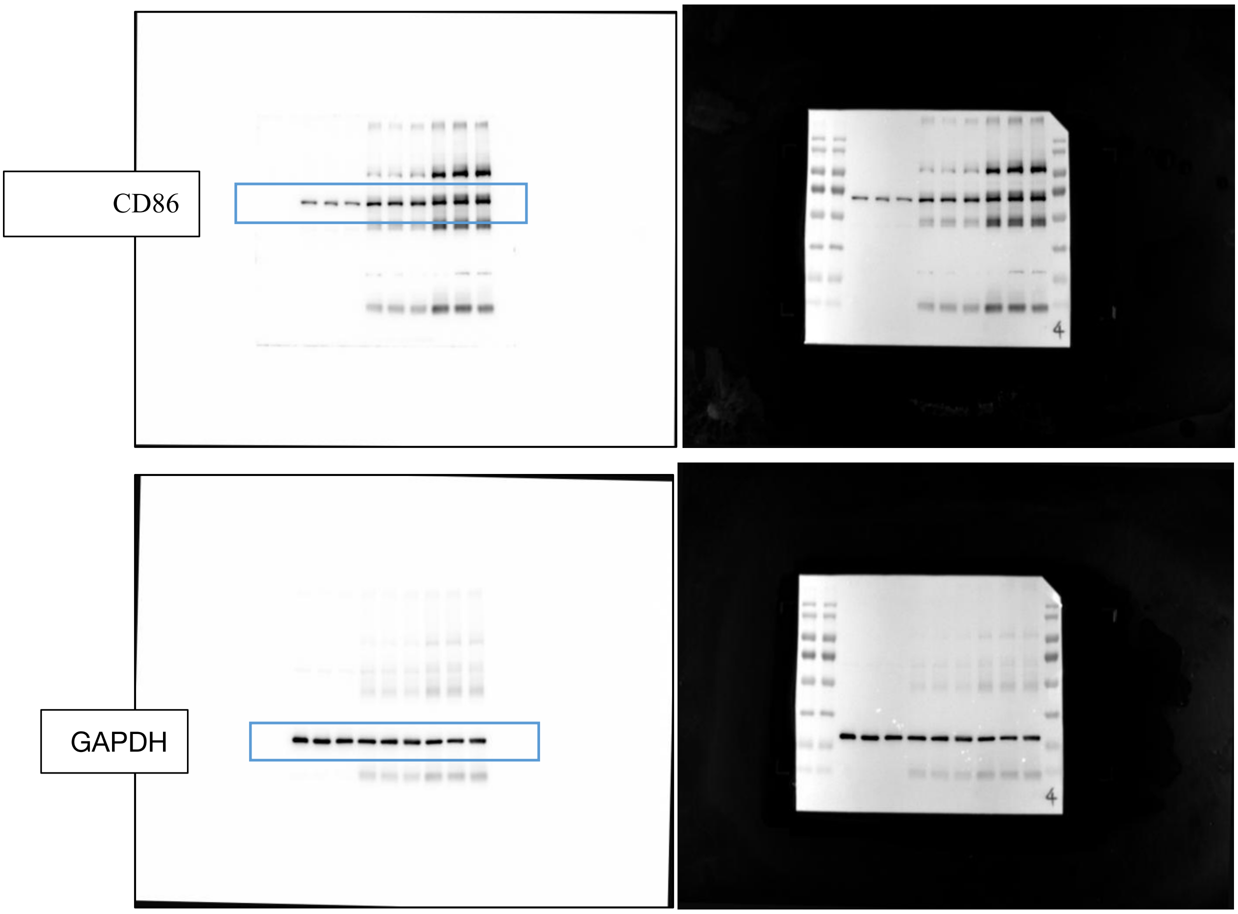
CD86
GAPDH
4
4

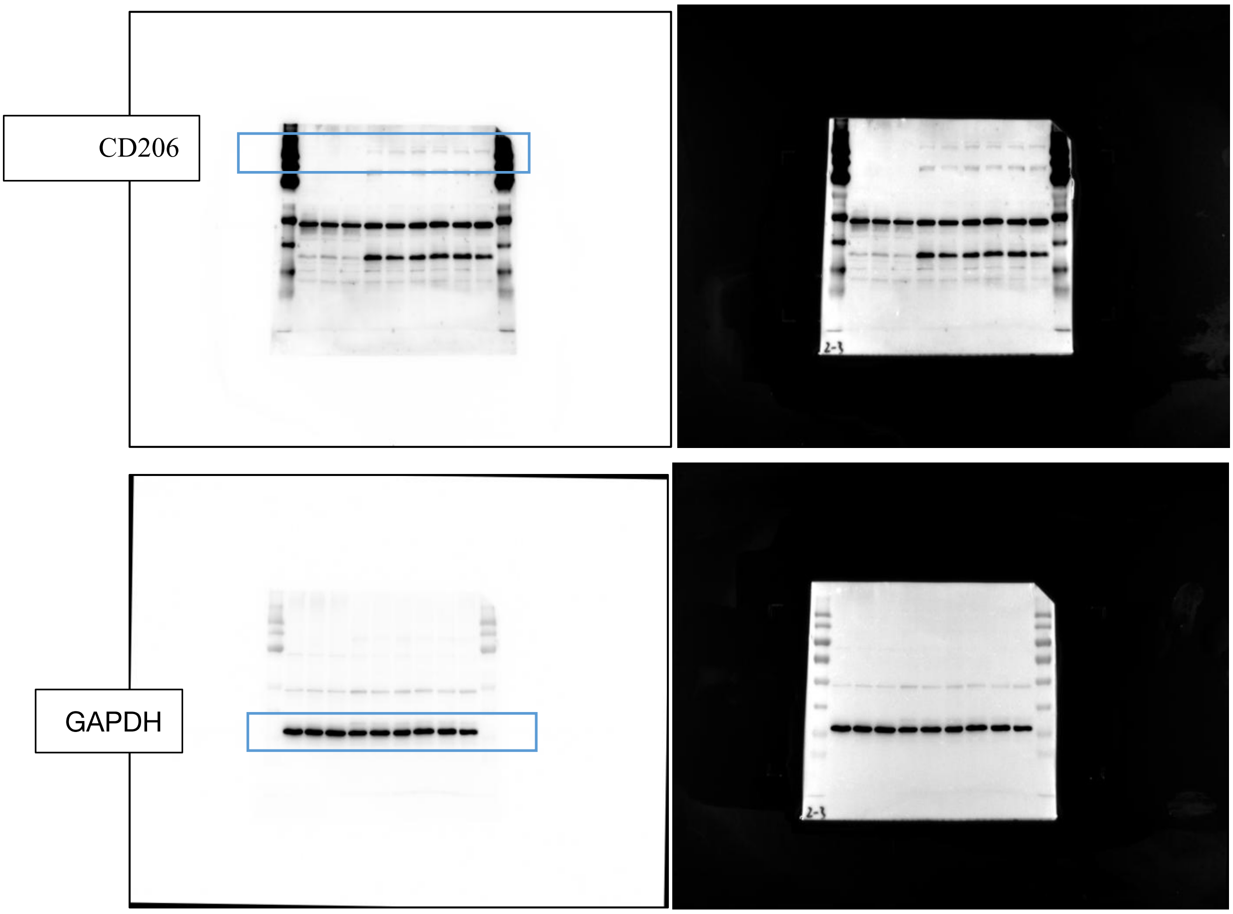
CD206
GAPDH

Figure 8a

The MARKER from top to bottom are sequentially 180, 130, 100, 70, 55, 40, 35, 25, 15

(ThermoFisher Scientific #26616).

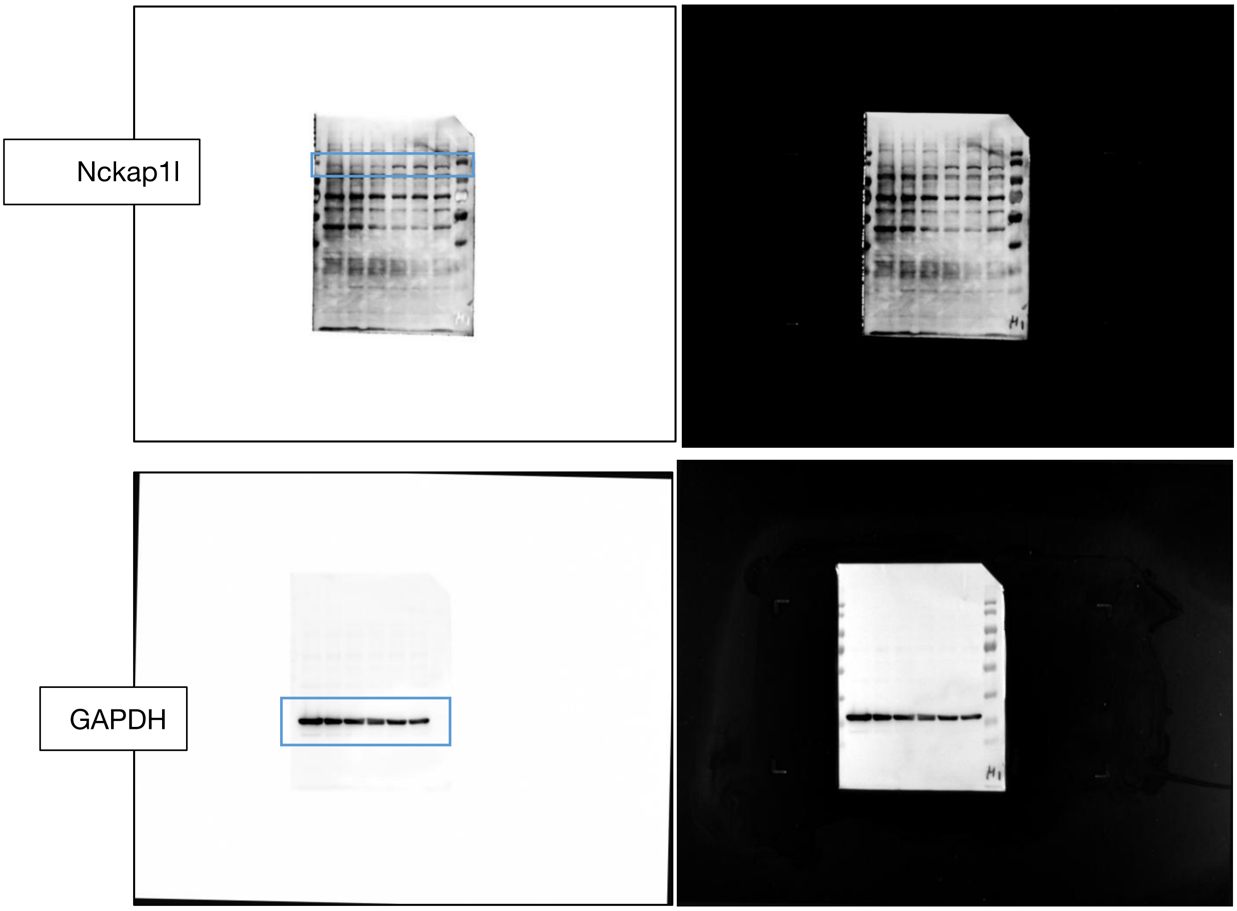

Figure 8c

The MARKER from top to bottom are sequentially 180, 130, 100, 70, 55, 40, 35, 25, 15

(ThermoFisher Scientific #26616).

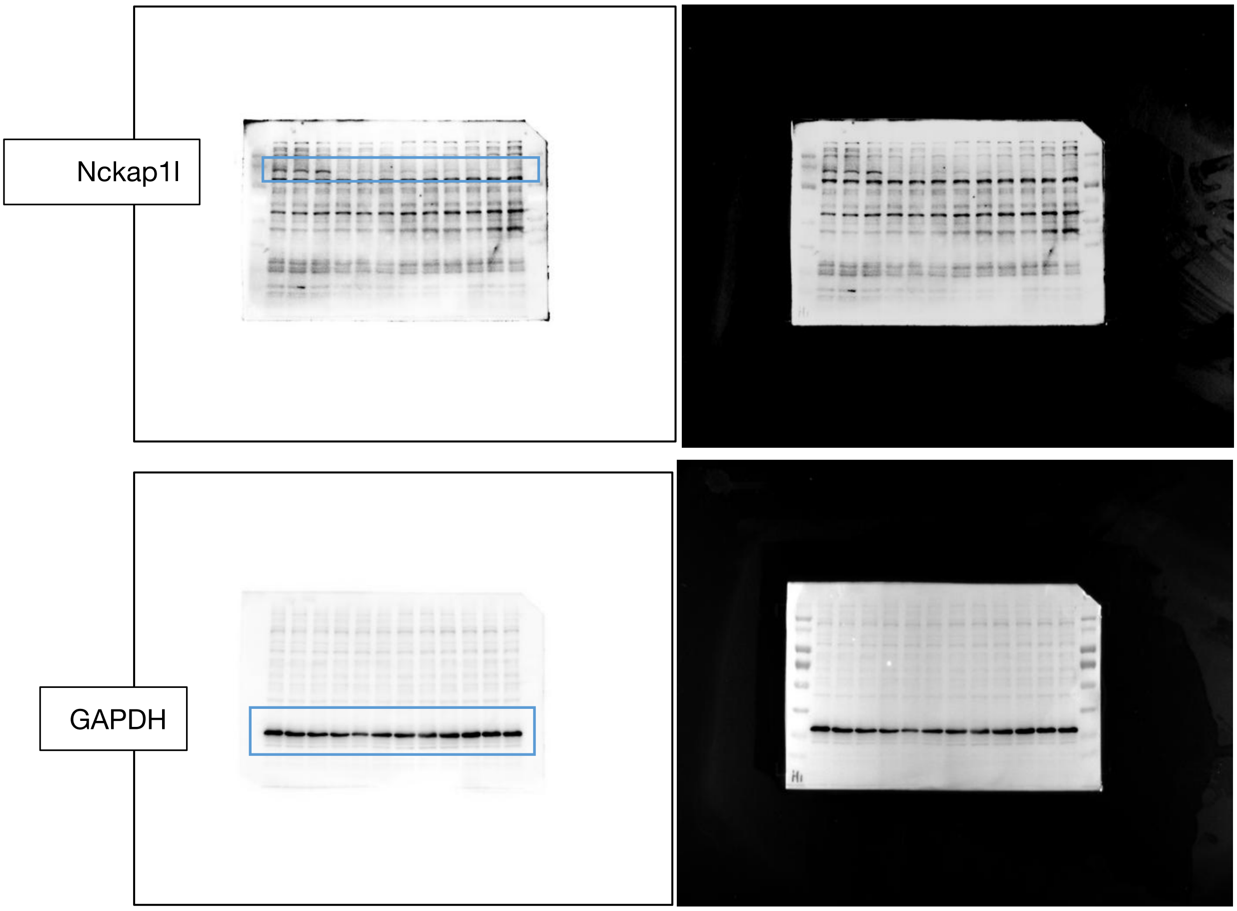

Figure 8e

The MARKER from top to bottom are sequentially 180, 130, 100, 70, 55, 40, 35, 25, 15 (ThermoFisher Scientific #26616).

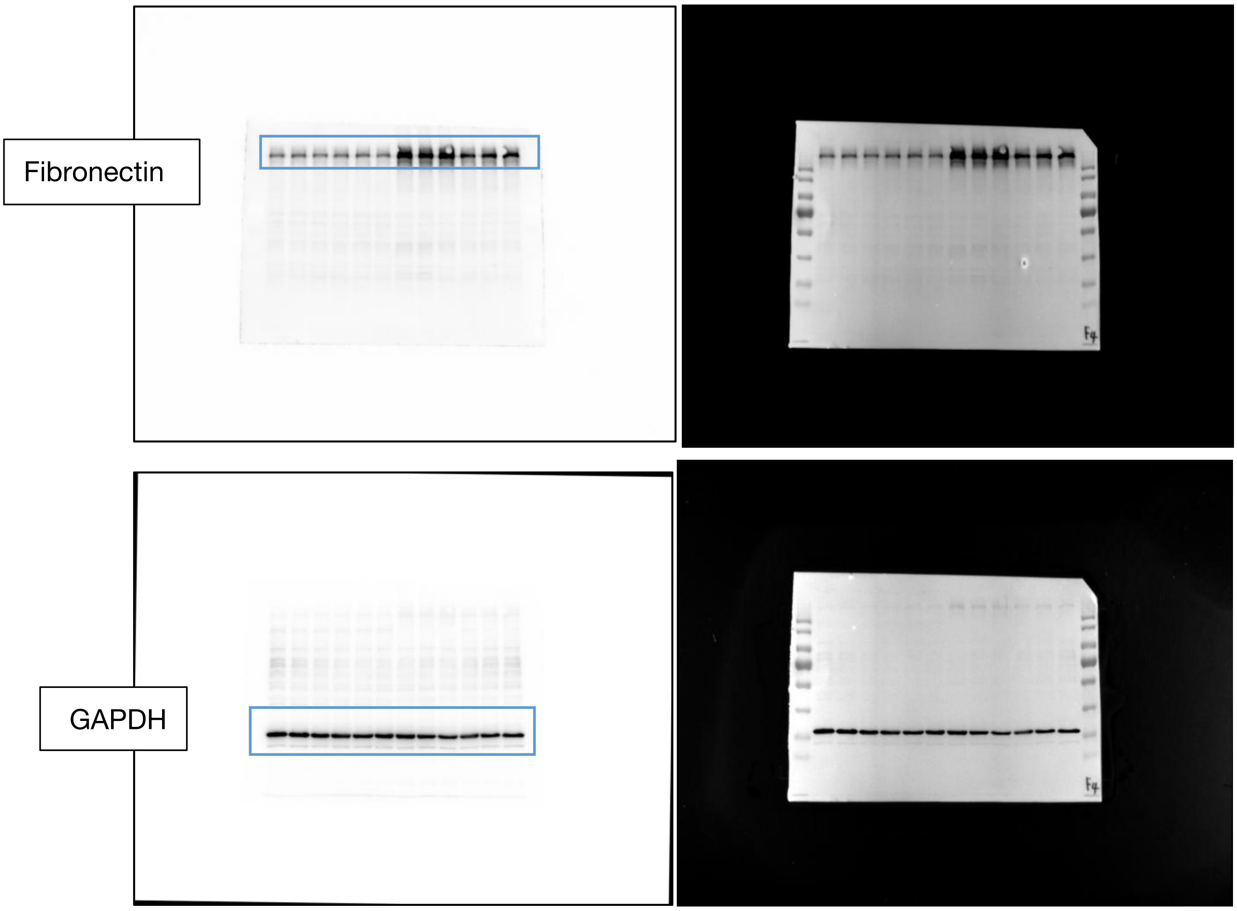

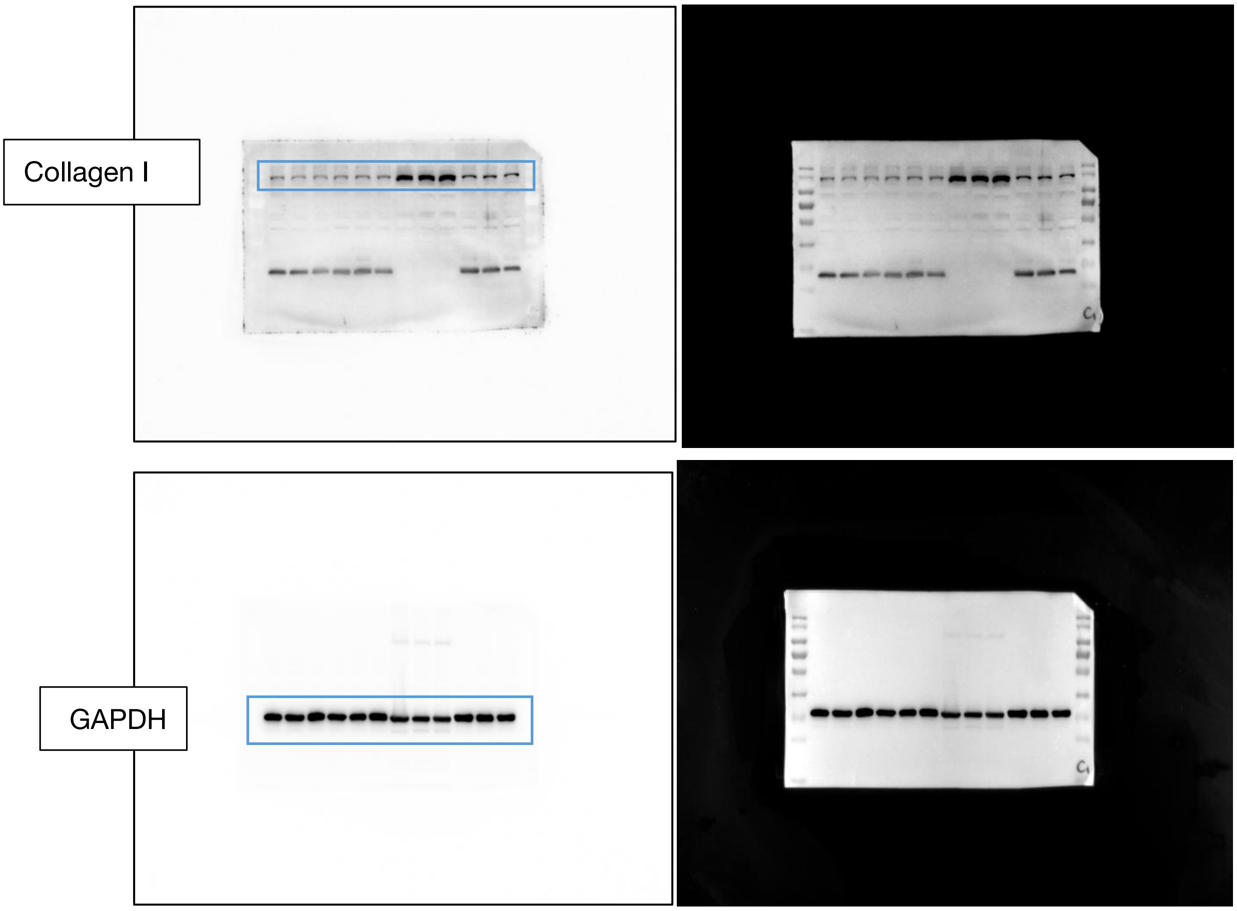
Collagen I
GAPDH

Figure 8h

The MARKER from top to bottom are sequentially 180, 130, 100, 70, 55, 40, 35, 25, 15

(ThermoFisher Scientific #26616).

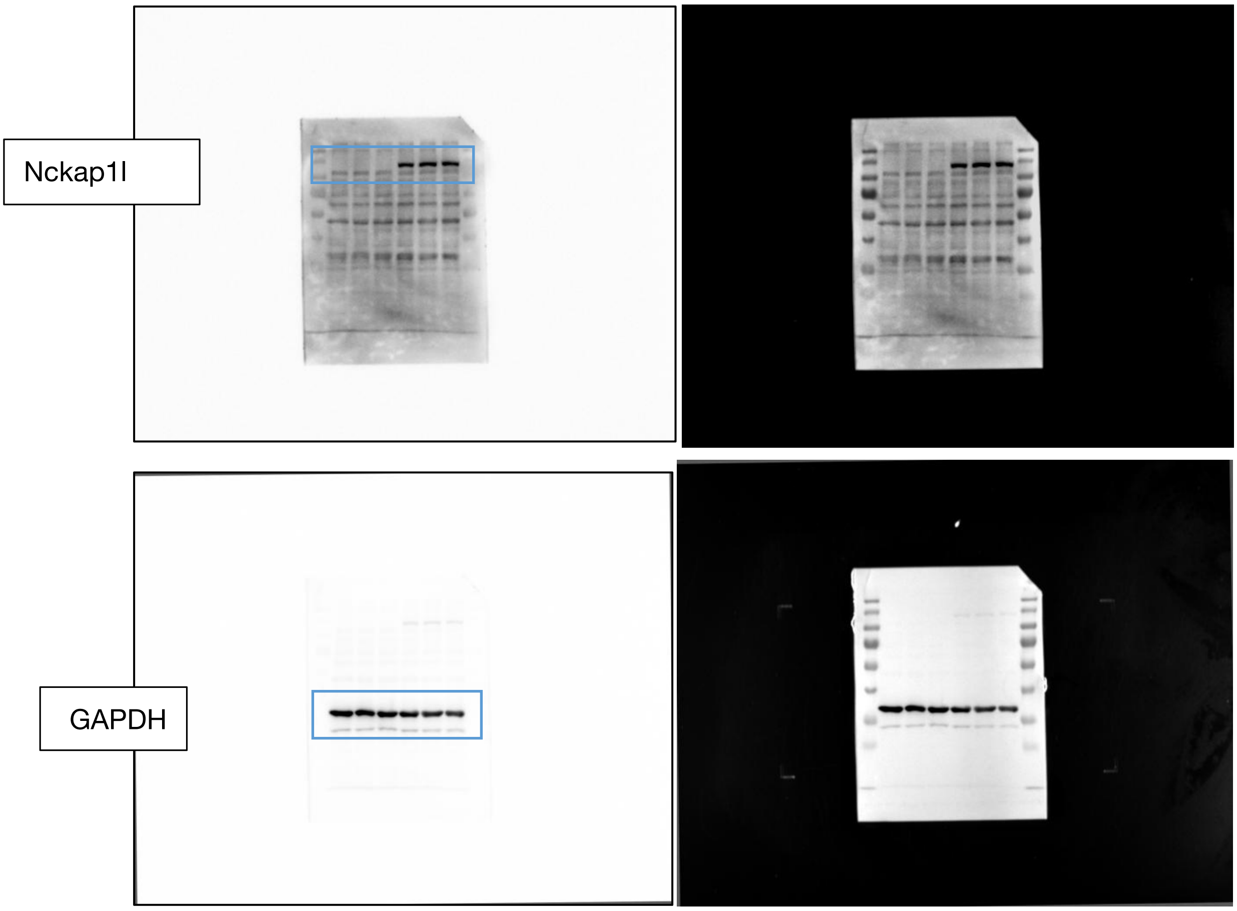

Figure 8j

The MARKER from top to bottom are sequentially 180, 130, 100, 70, 55, 40, 35, 25, 15

(ThermoFisher Scientific #26616).

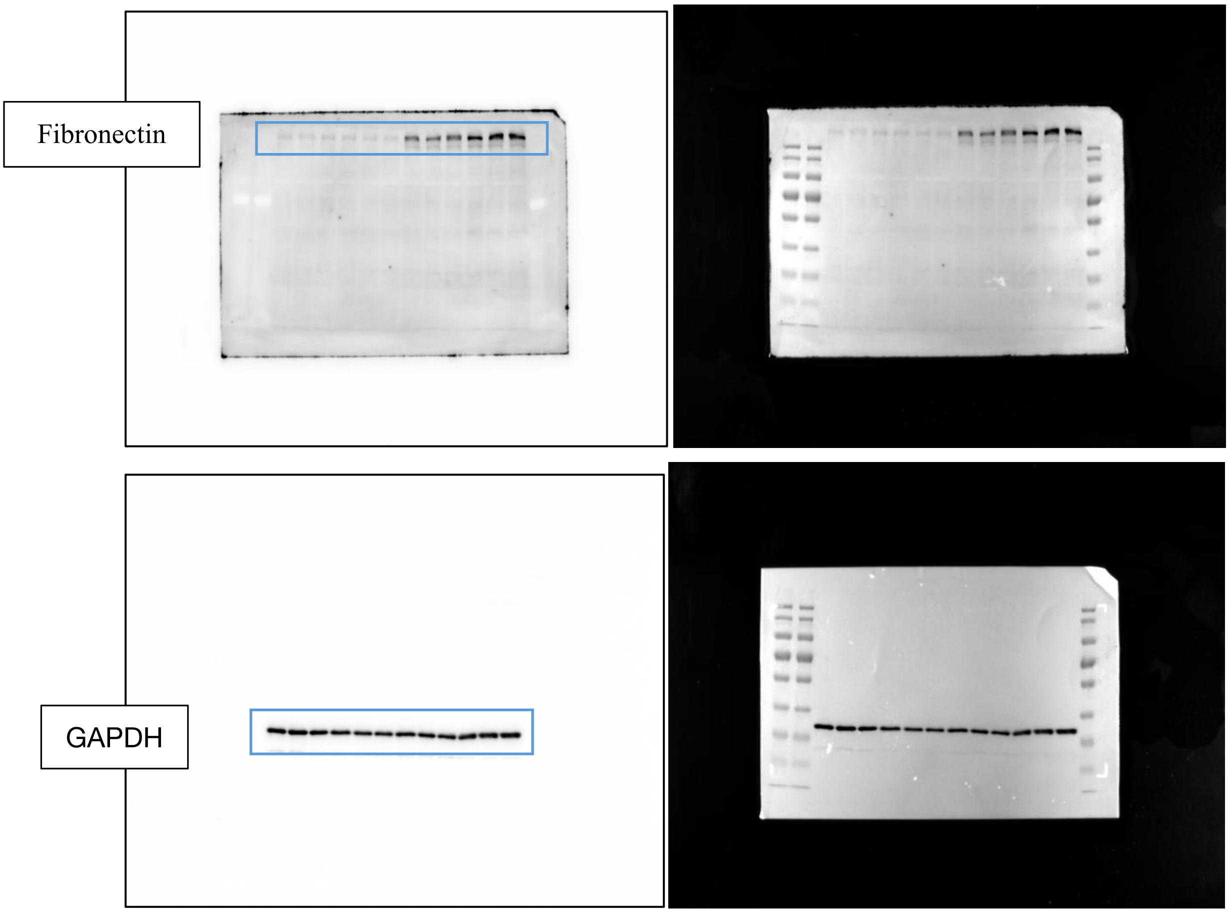

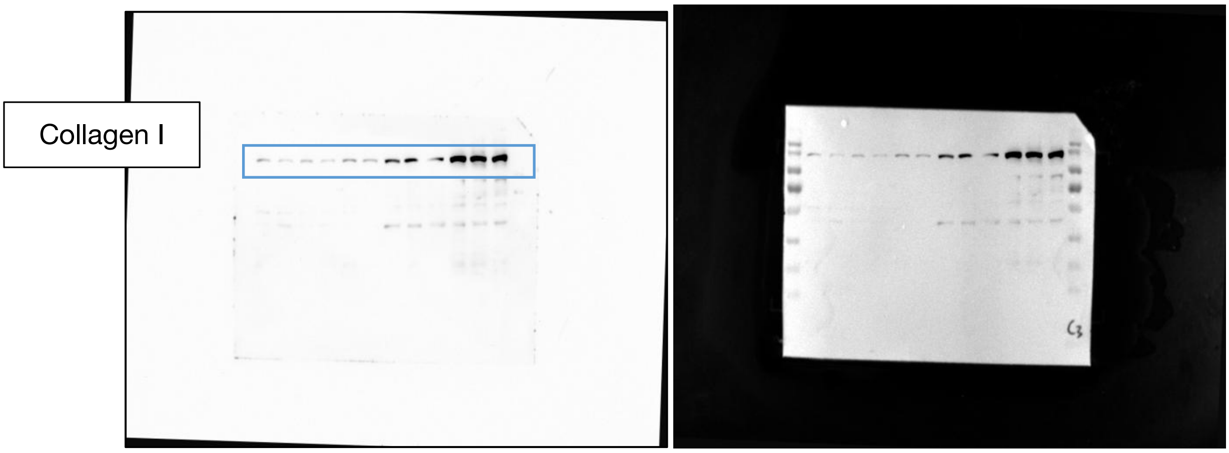
Collagen I

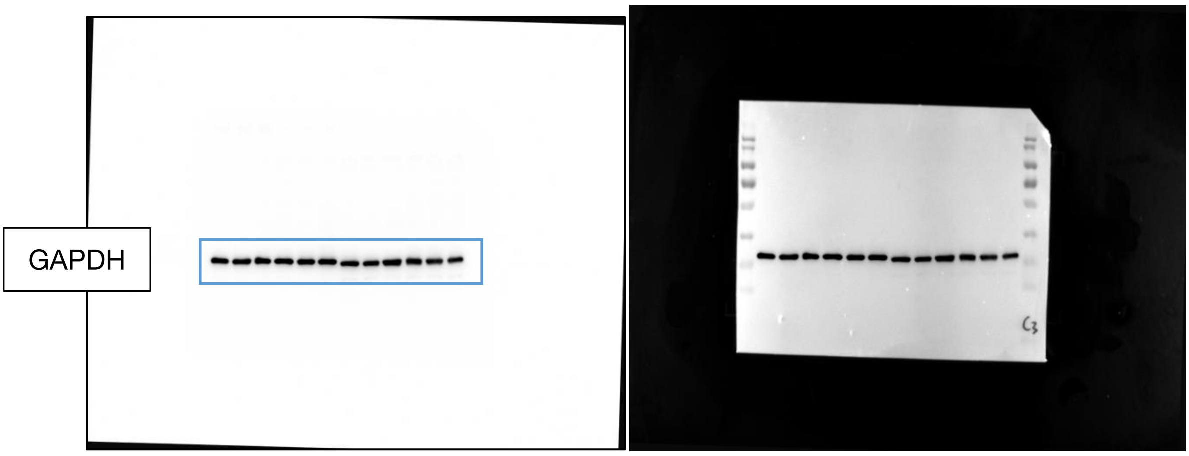
GAPDH

Figure 8l

The MARKER from top to bottom are sequentially 180, 130, 100, 70, 55, 40, 35, 25, 15

(ThermoFisher Scientific #26616).

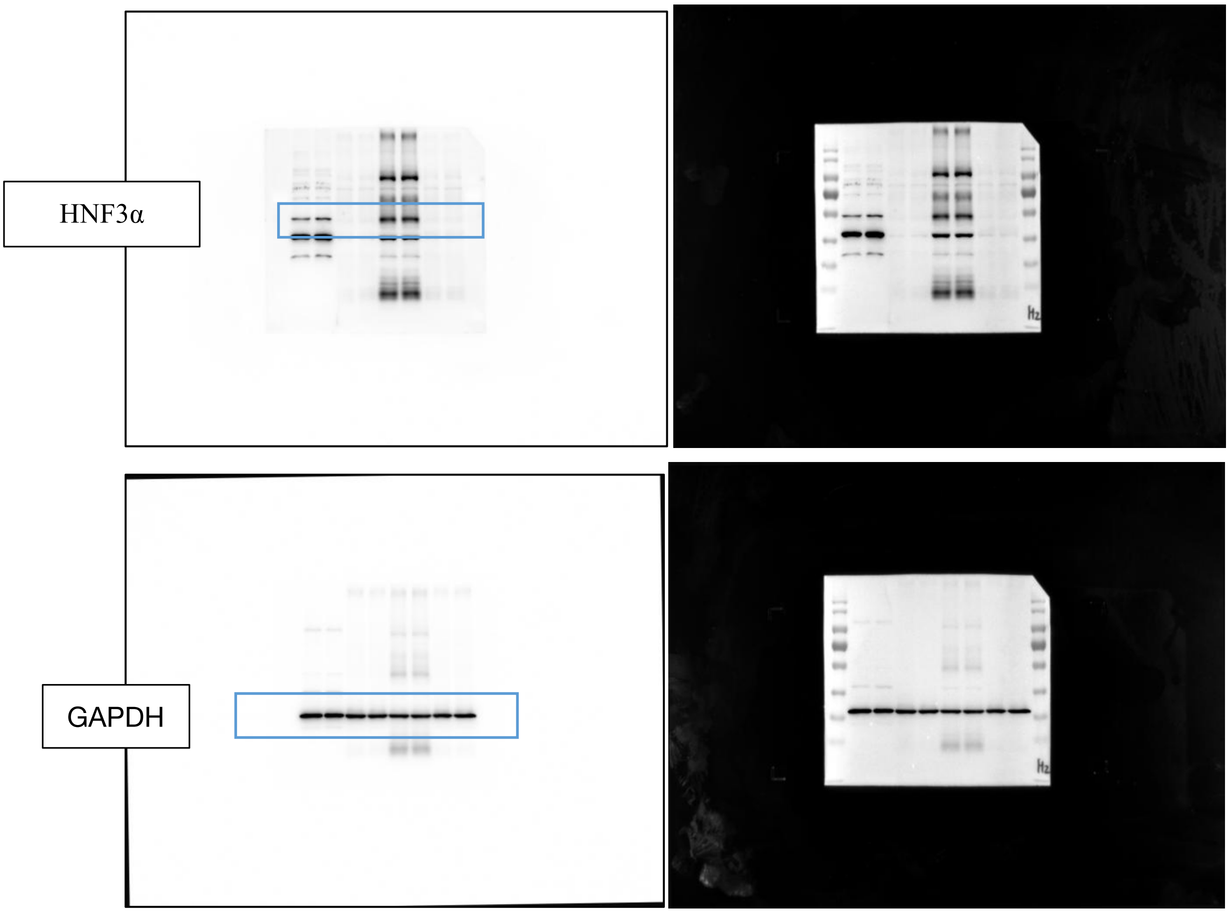

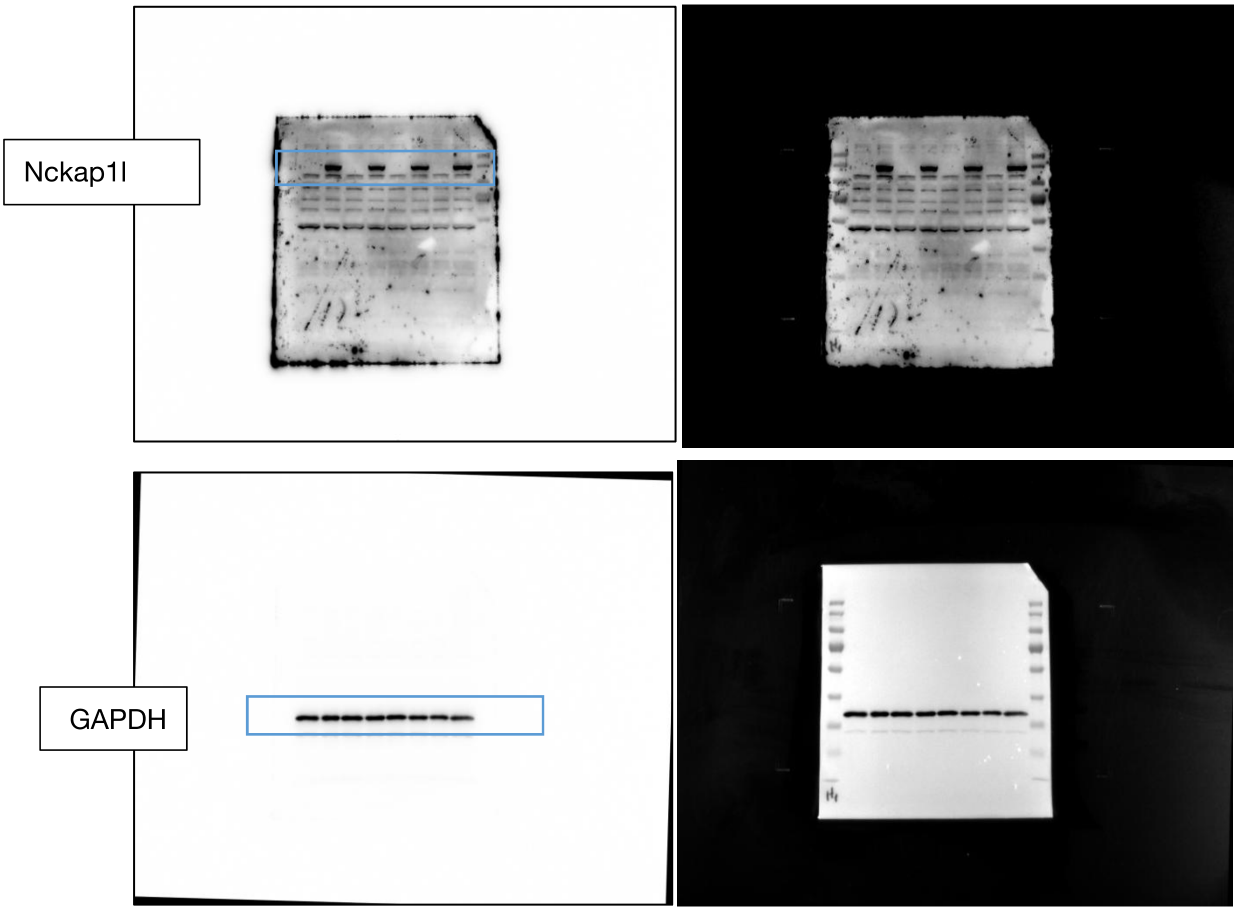

Nckap1l
GAPDH

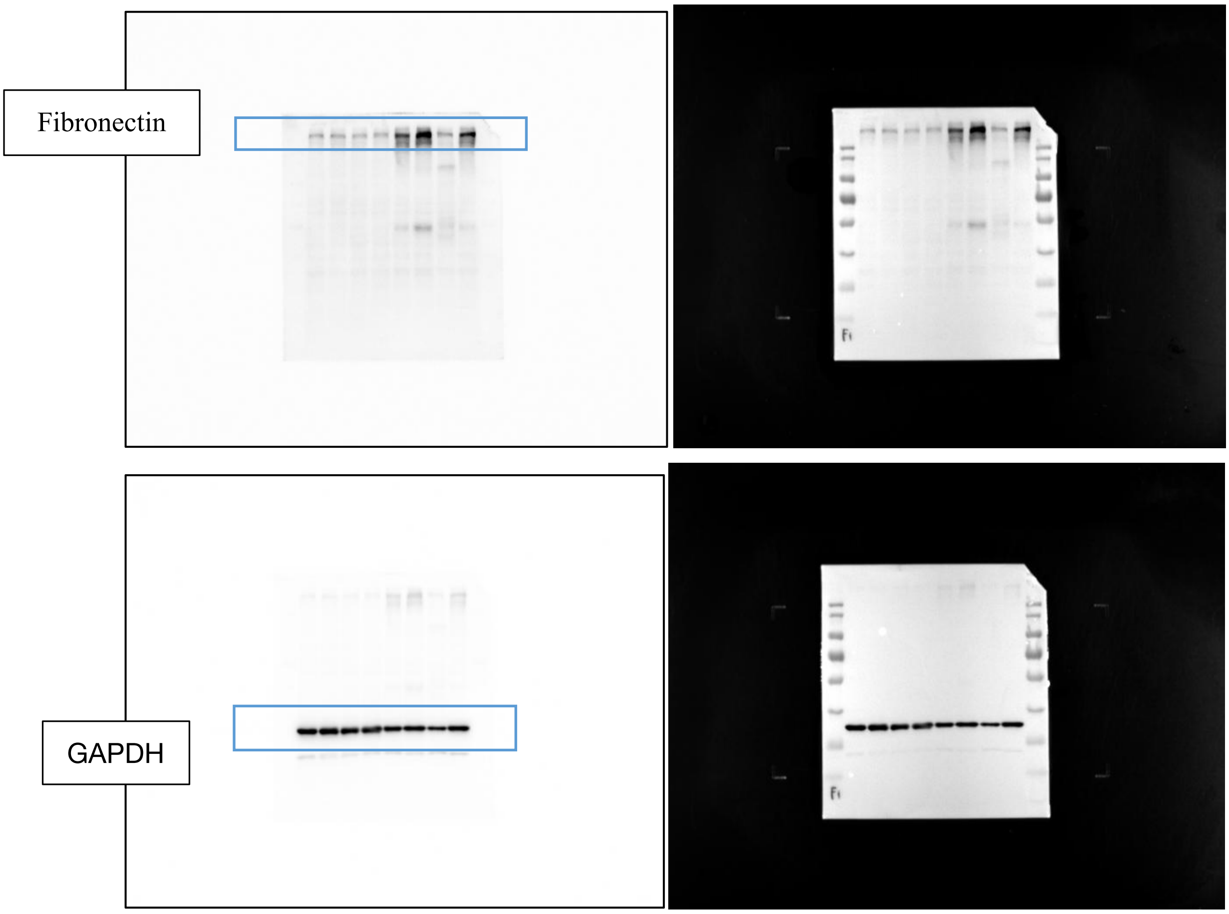
Fibronectin
GAPDH

Figure 8p

The MARKER from top to bottom are sequentially 180, 130, 100, 70, 55, 40, 35, 25, 15 (ThermoFisher Scientific #26616).

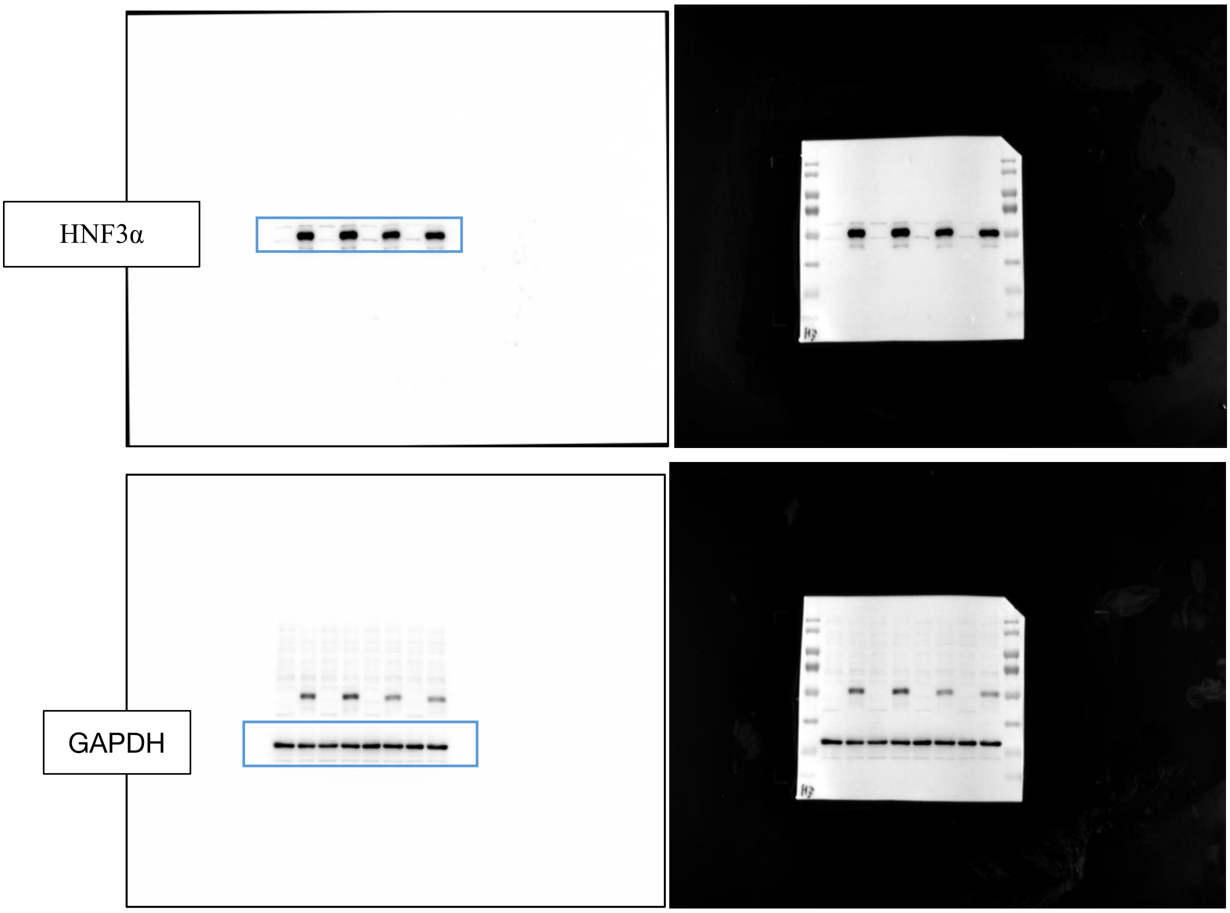

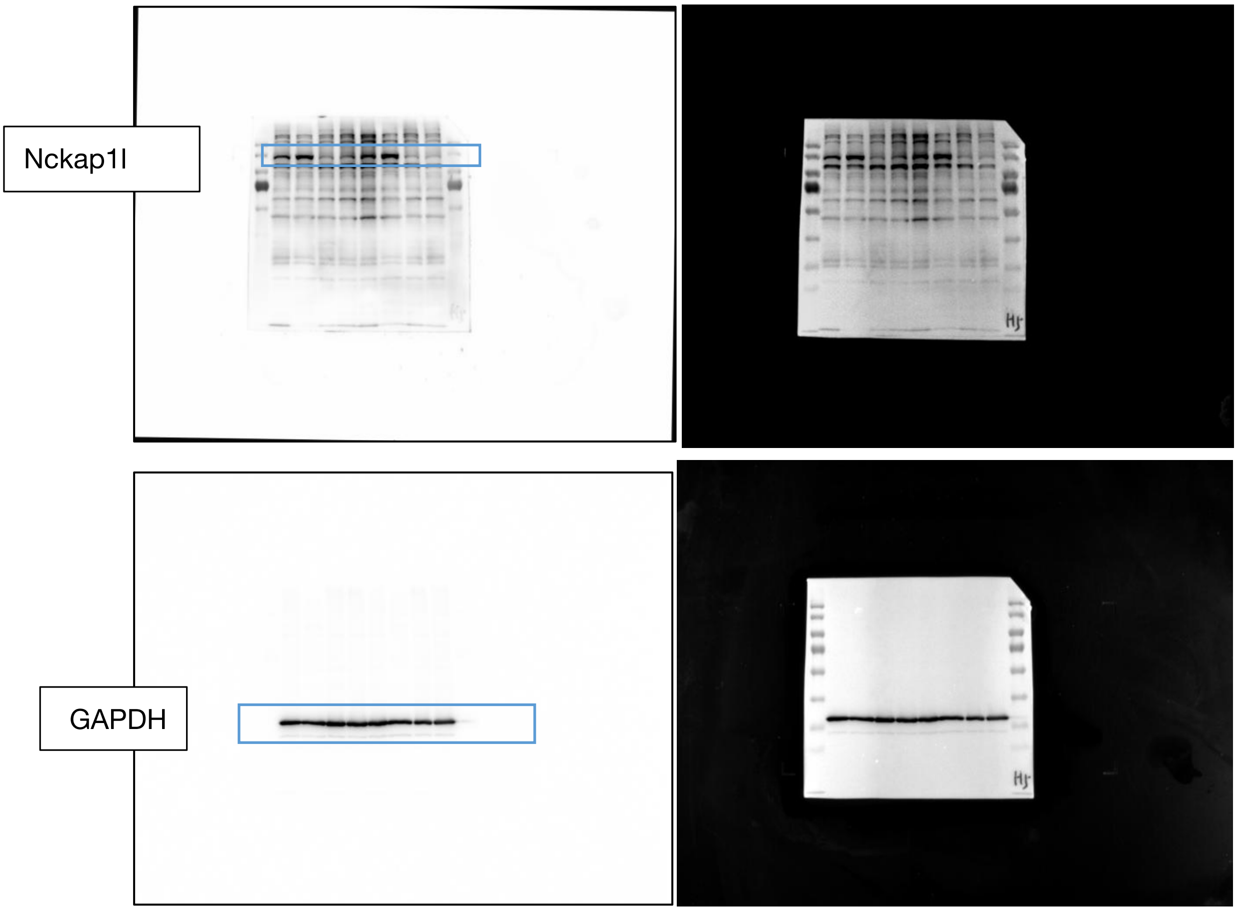
Nckap1l
GAPDH

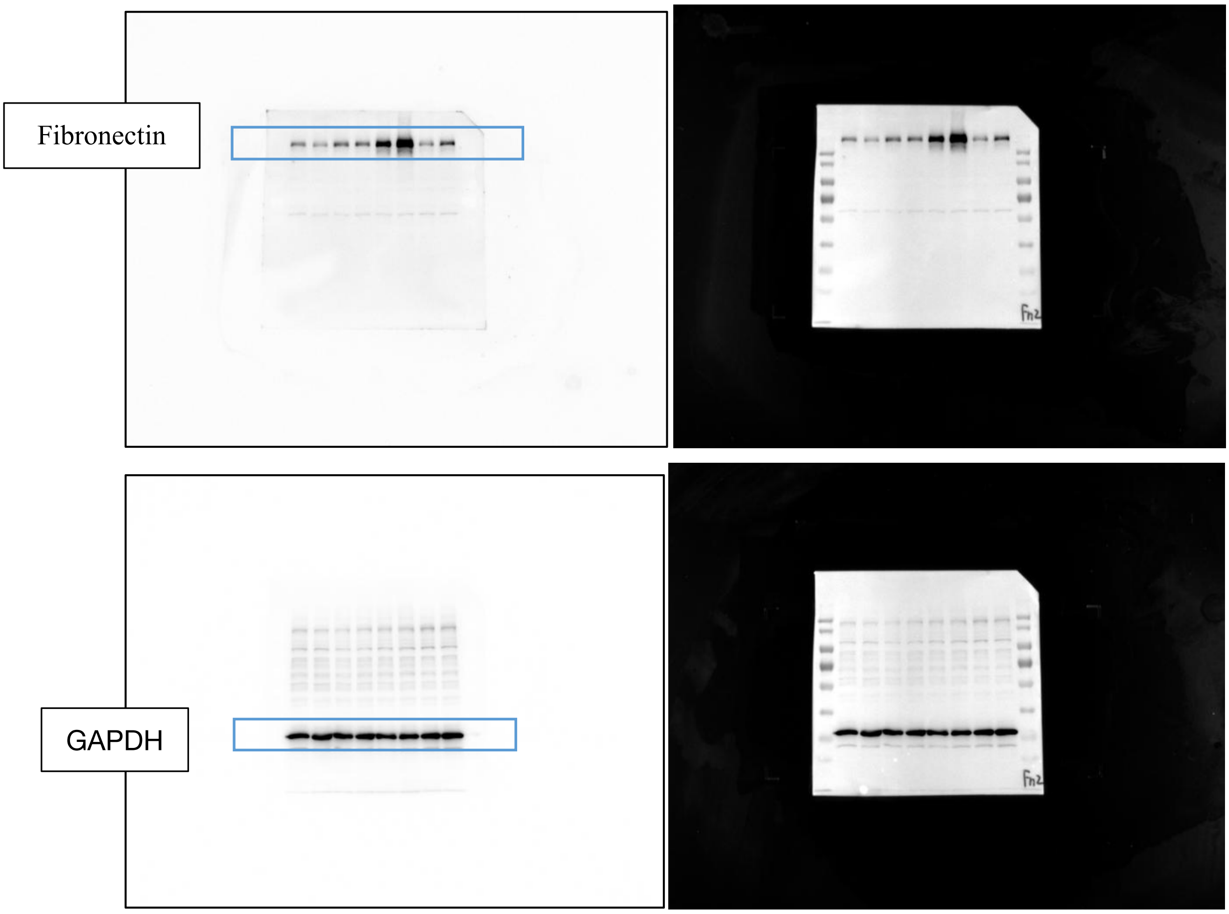

Fibronectin
GAPDH

Supplementary Figure 3e

The MARKER from top to bottom are sequentially 180, 130, 100, 70, 55, 40, 35, 25, 15 (ThermoFisher Scientific #26616).

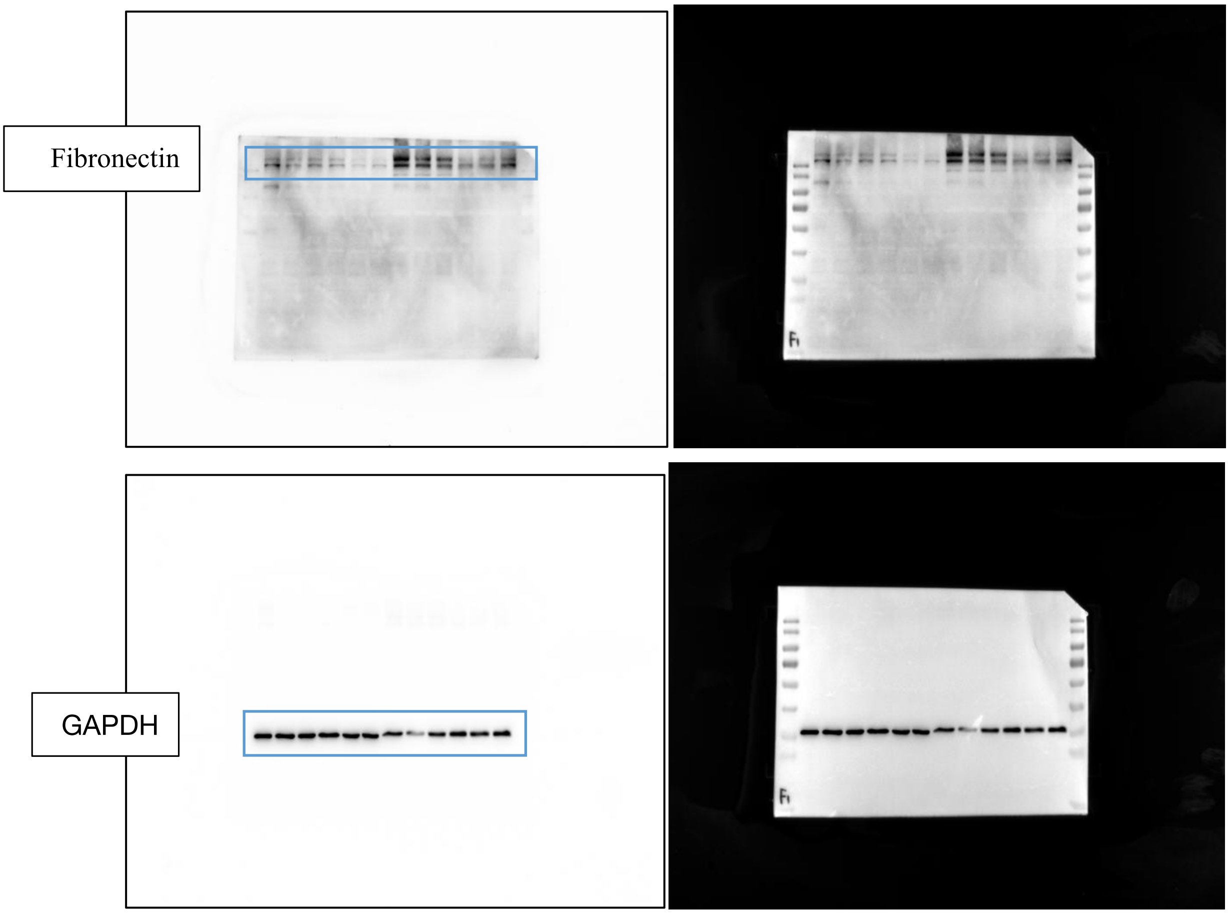

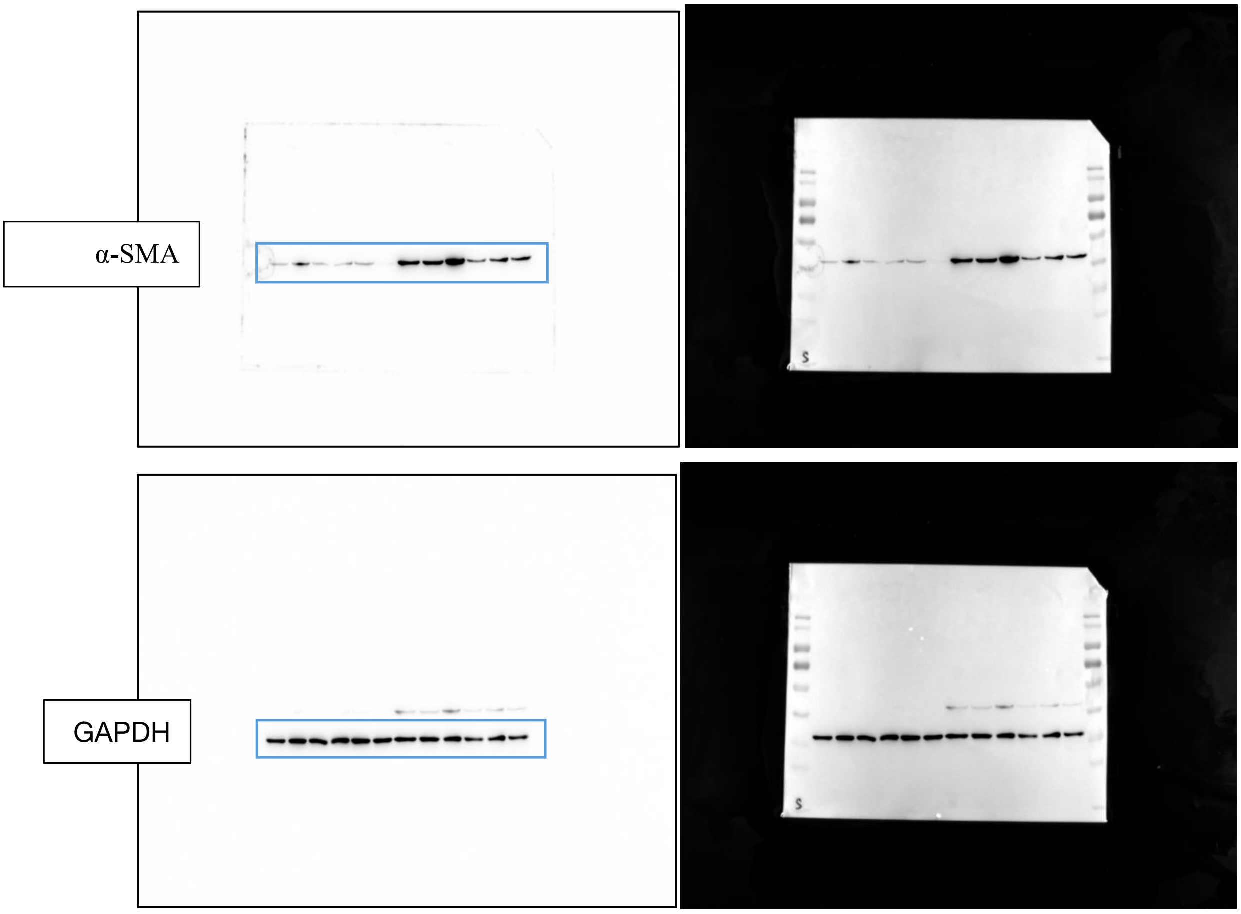
α-SMA
GAPDH

Supplementary Figure 4c

The MARKER from top to bottom are sequentially 180, 130, 100, 70, 55, 40, 35, 25, 15 (ThermoFisher Scientific #26616).

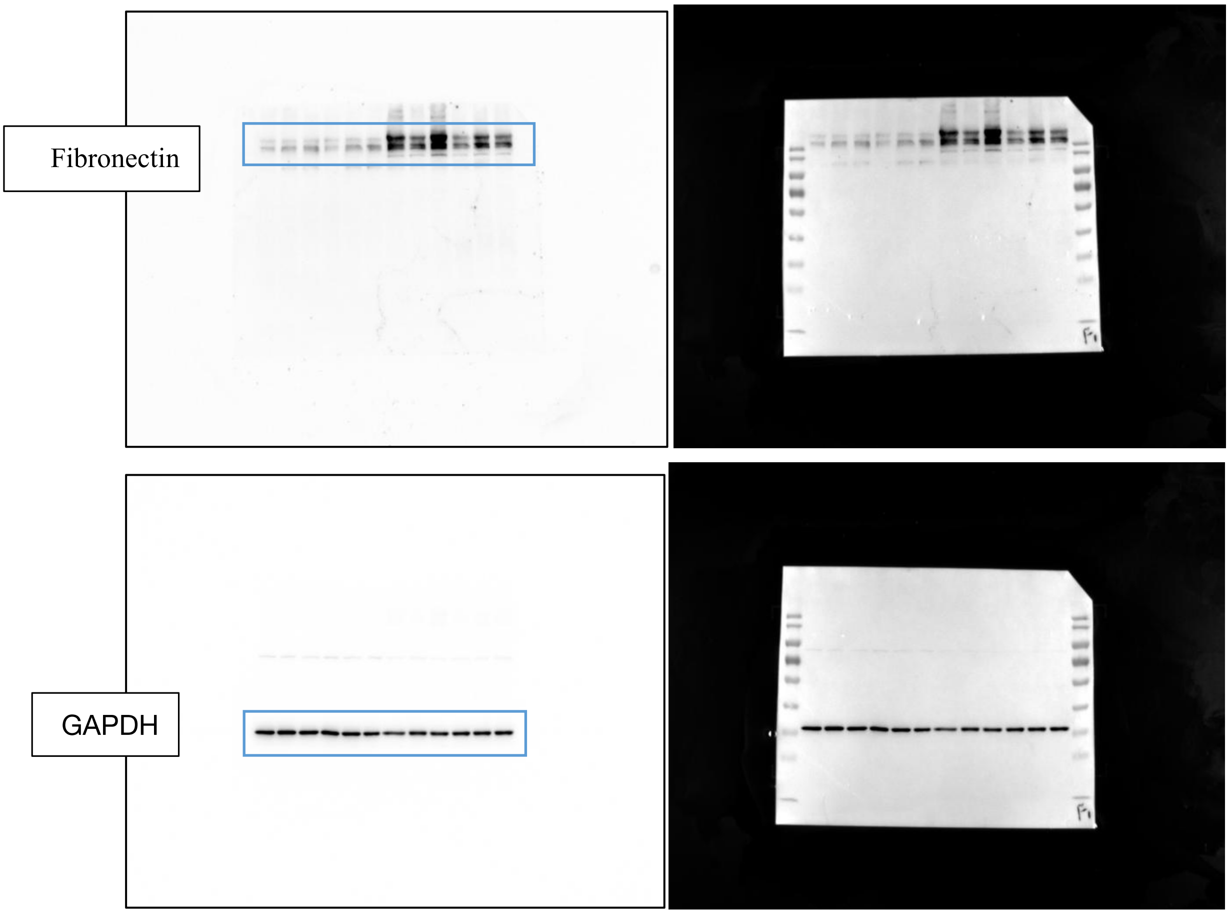

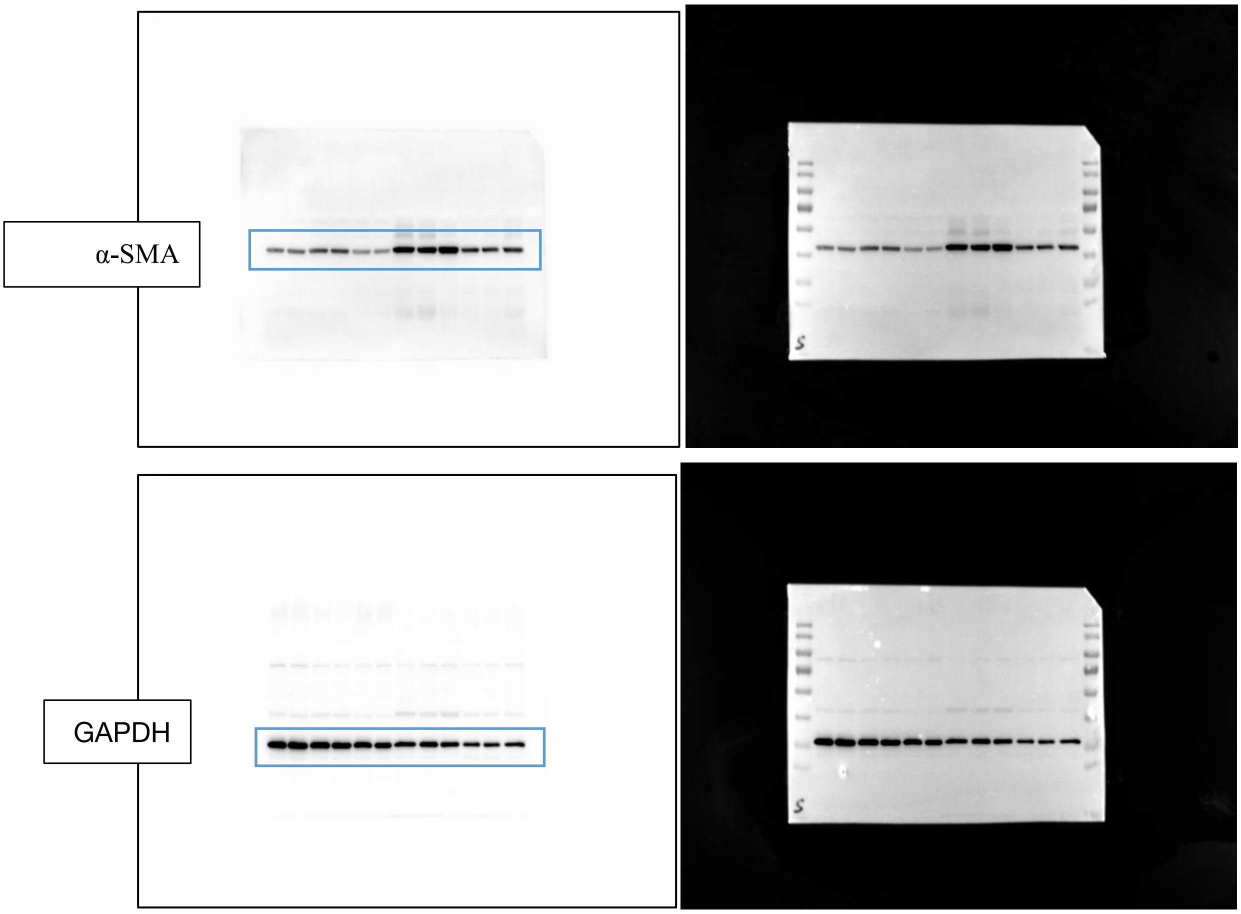
α-SMA
GAPDH

Supplementary Figure 5a

The MARKER from top to bottom are sequentially 180, 130, 100, 70, 55, 40, 35, 25, 15 (ThermoFisher Scientific #26616).

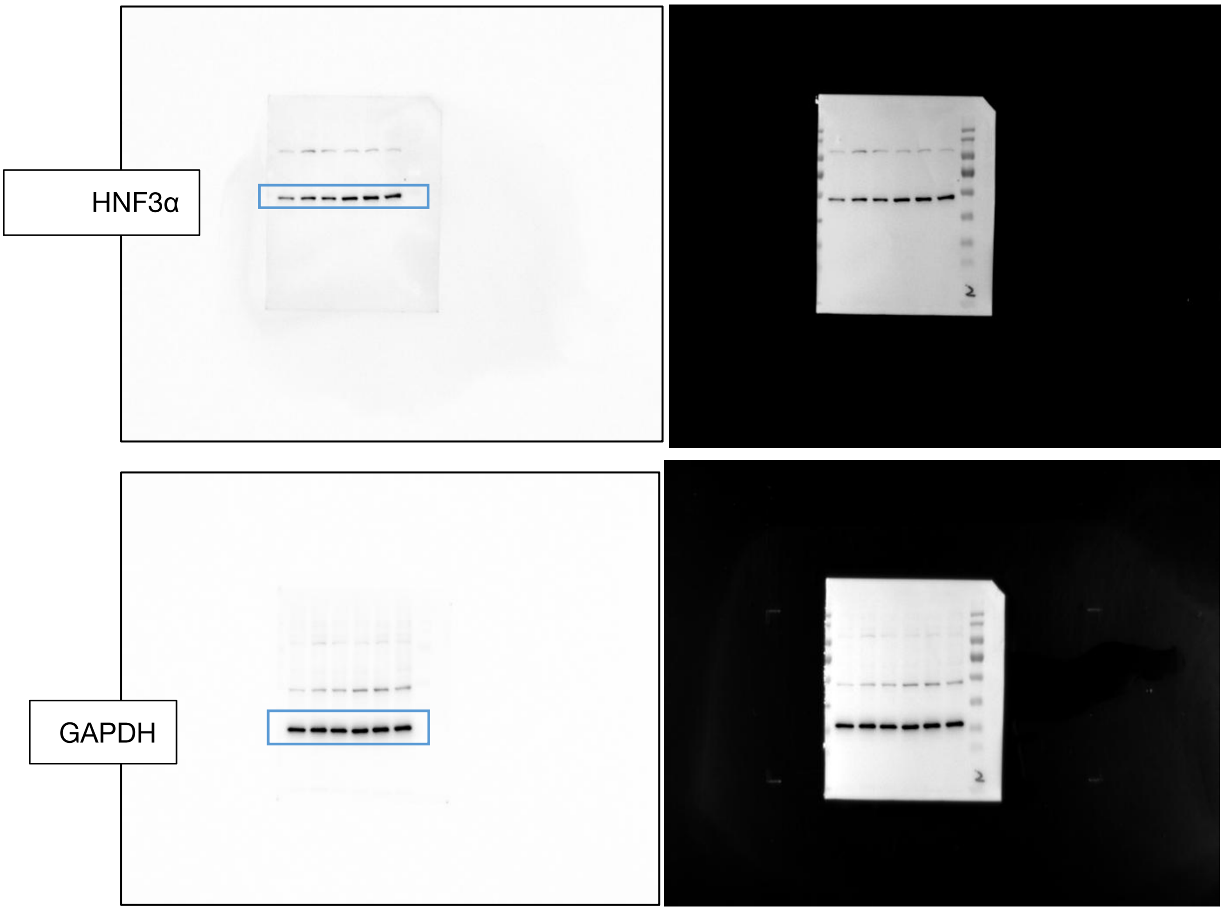

Supplementary Figure 5c

The MARKER from top to bottom are sequentially 180, 130, 100, 70, 55, 40, 35, 25, 15 (ThermoFisher Scientific #26616).

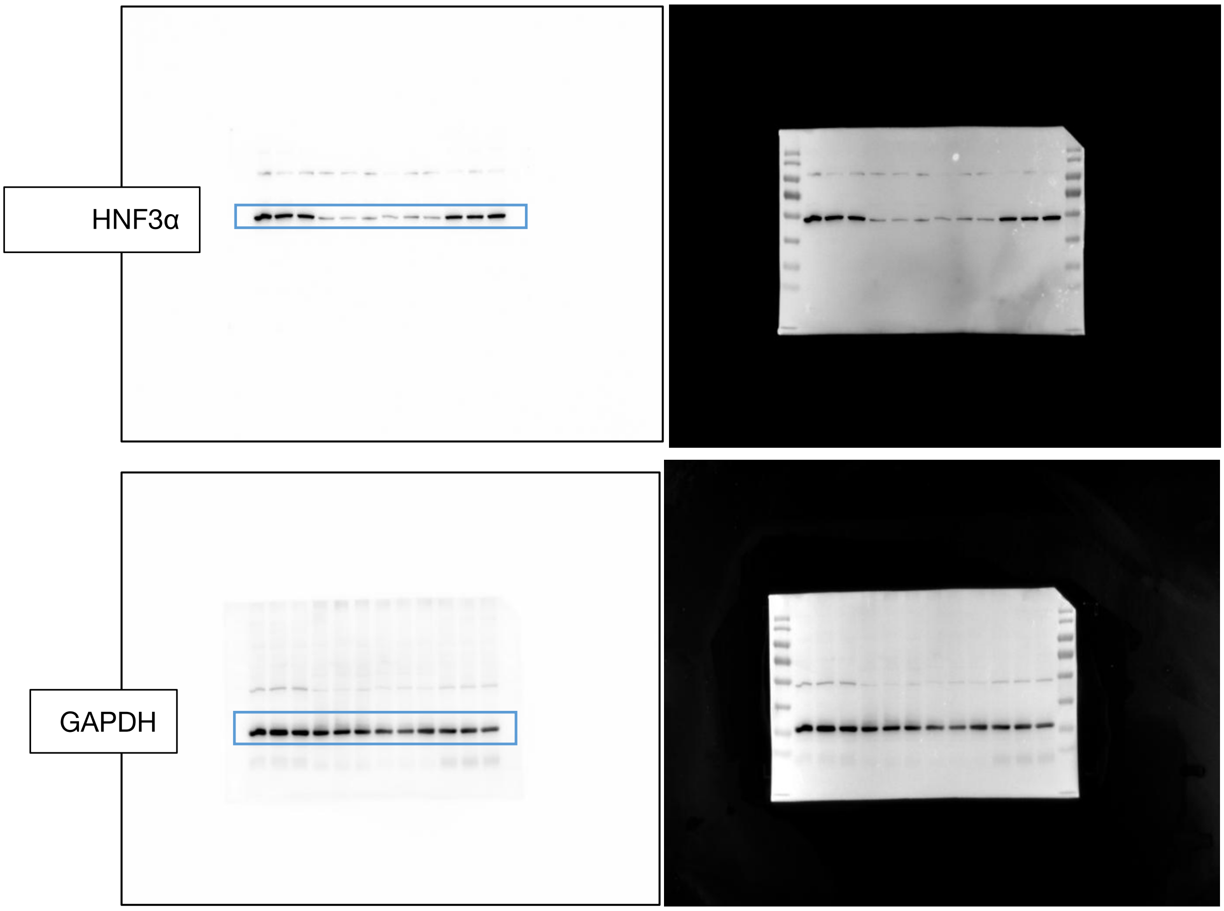

Supplementary Figure 5e

The MARKER from top to bottom are sequentially 180, 130, 100, 70, 55, 40, 35, 25, 15 (ThermoFisher Scientific #26616).

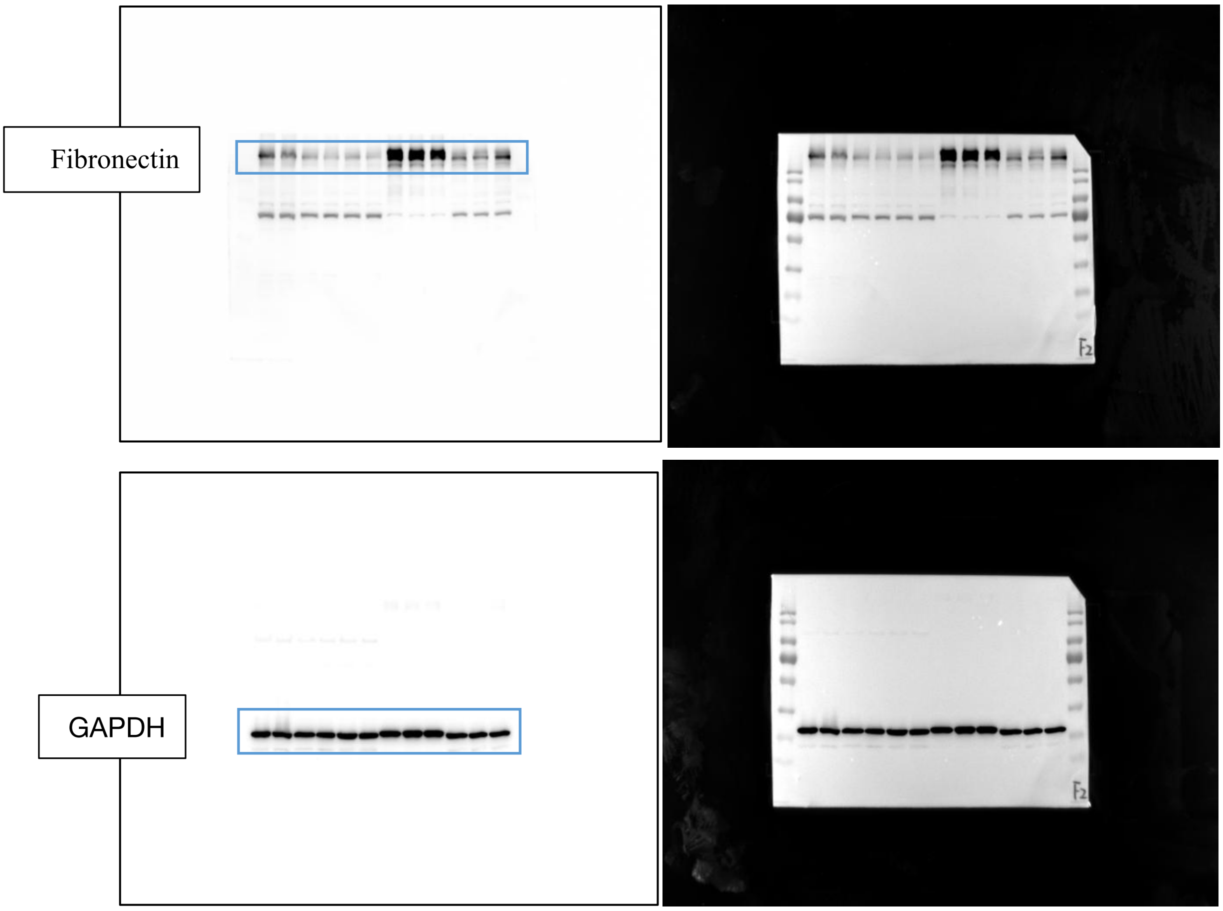

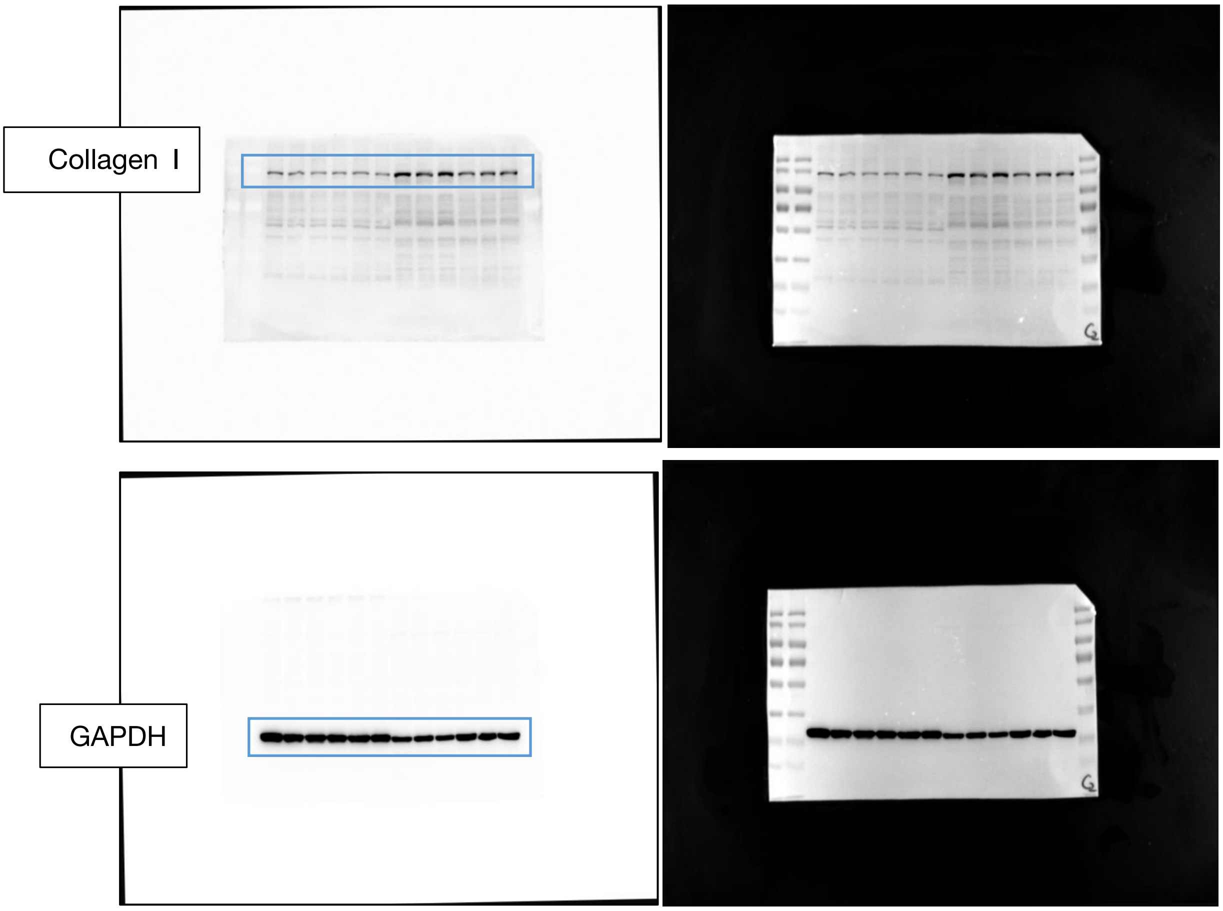
Collagen I
GAPDH

Supplementary Figure 5g

The MARKER from top to bottom are sequentially 180, 130, 100, 70, 55, 40, 35, 25, 15 (ThermoFisher Scientific #26616).

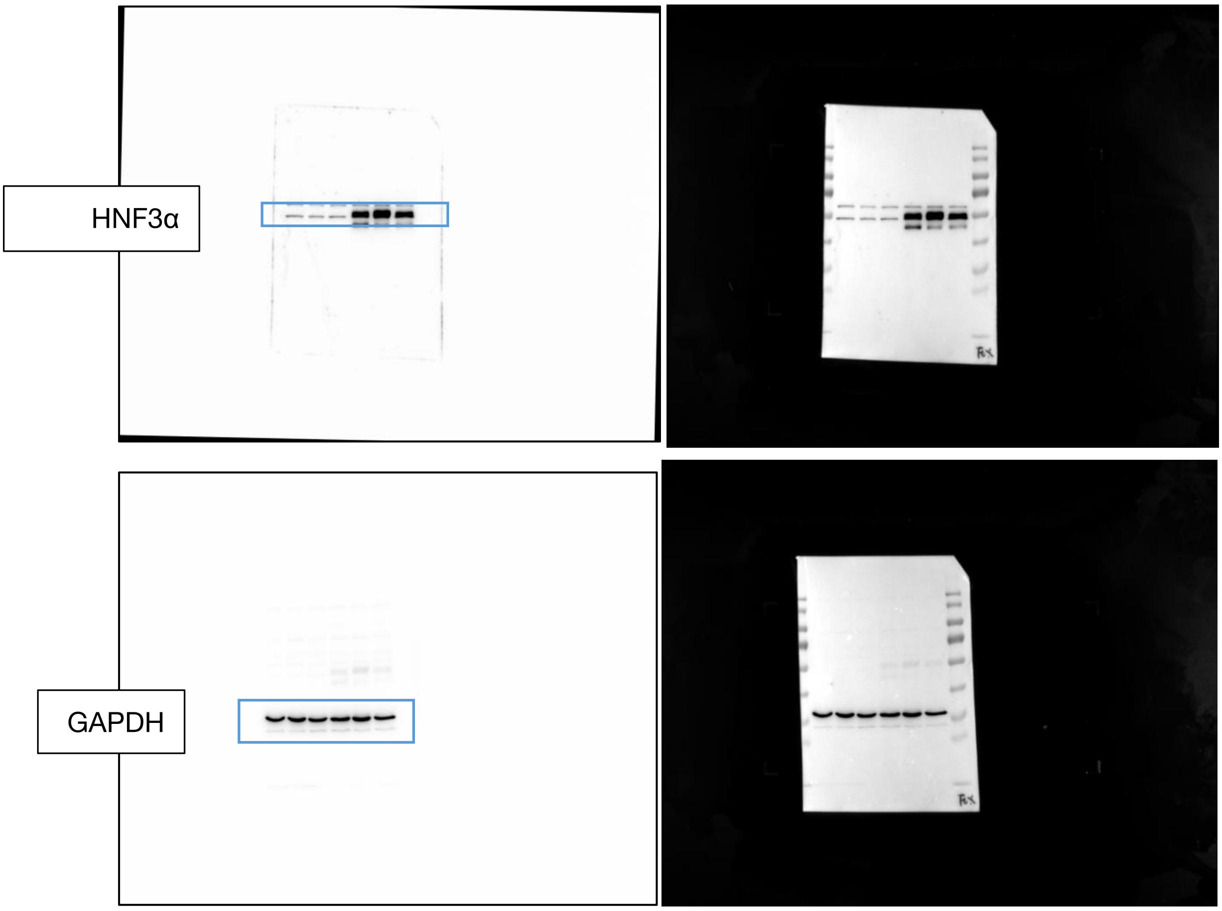

Supplementary Figure 5i

The MARKER from top to bottom are sequentially 180, 130, 100, 70, 55, 40, 35, 25, 15 (ThermoFisher Scientific #26616).

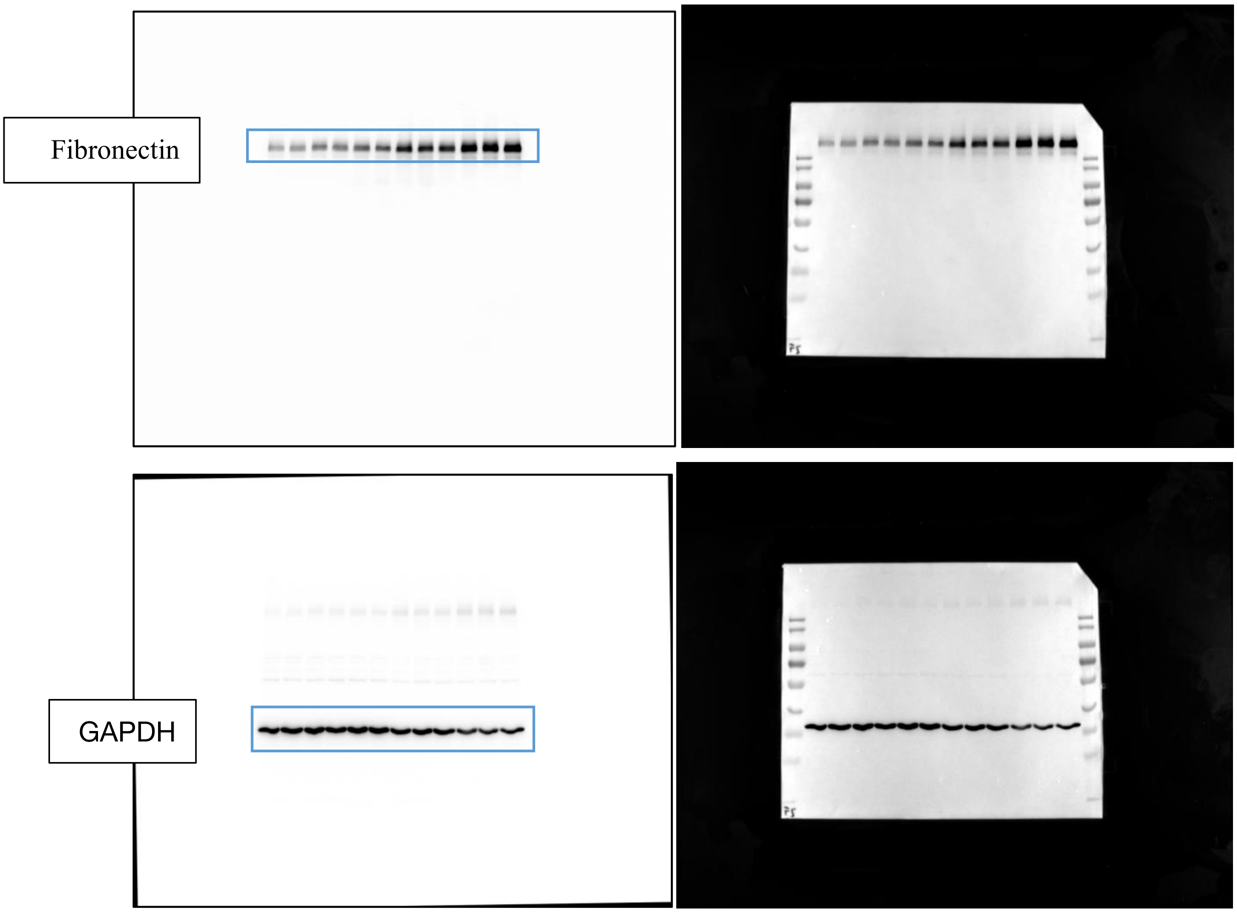

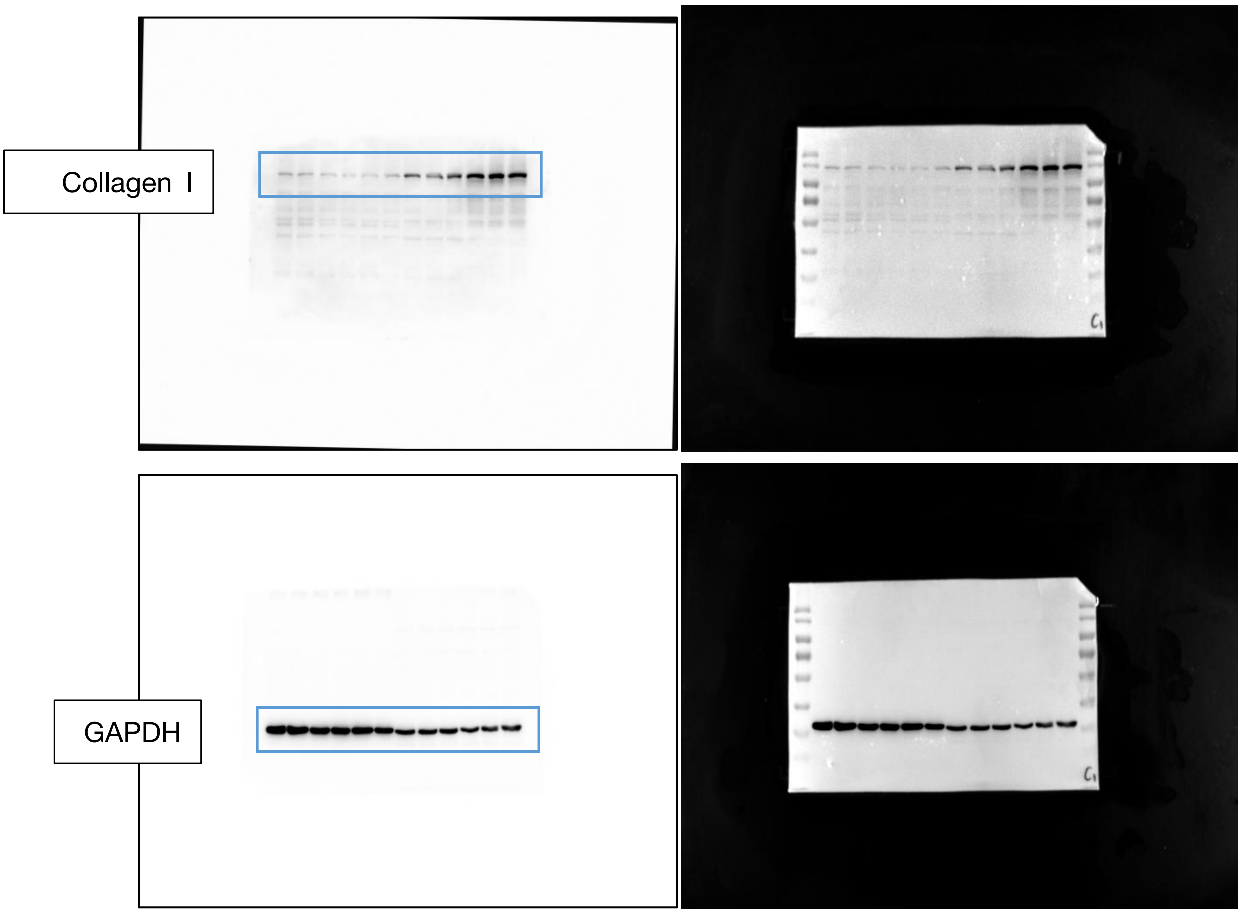
Collagen I
GAPDH

Supplementary Figure 5k

The MARKER from top to bottom are sequentially 180, 130, 100, 70, 55, 40, 35, 25, 15

(ThermoFisher Scientific #26616).

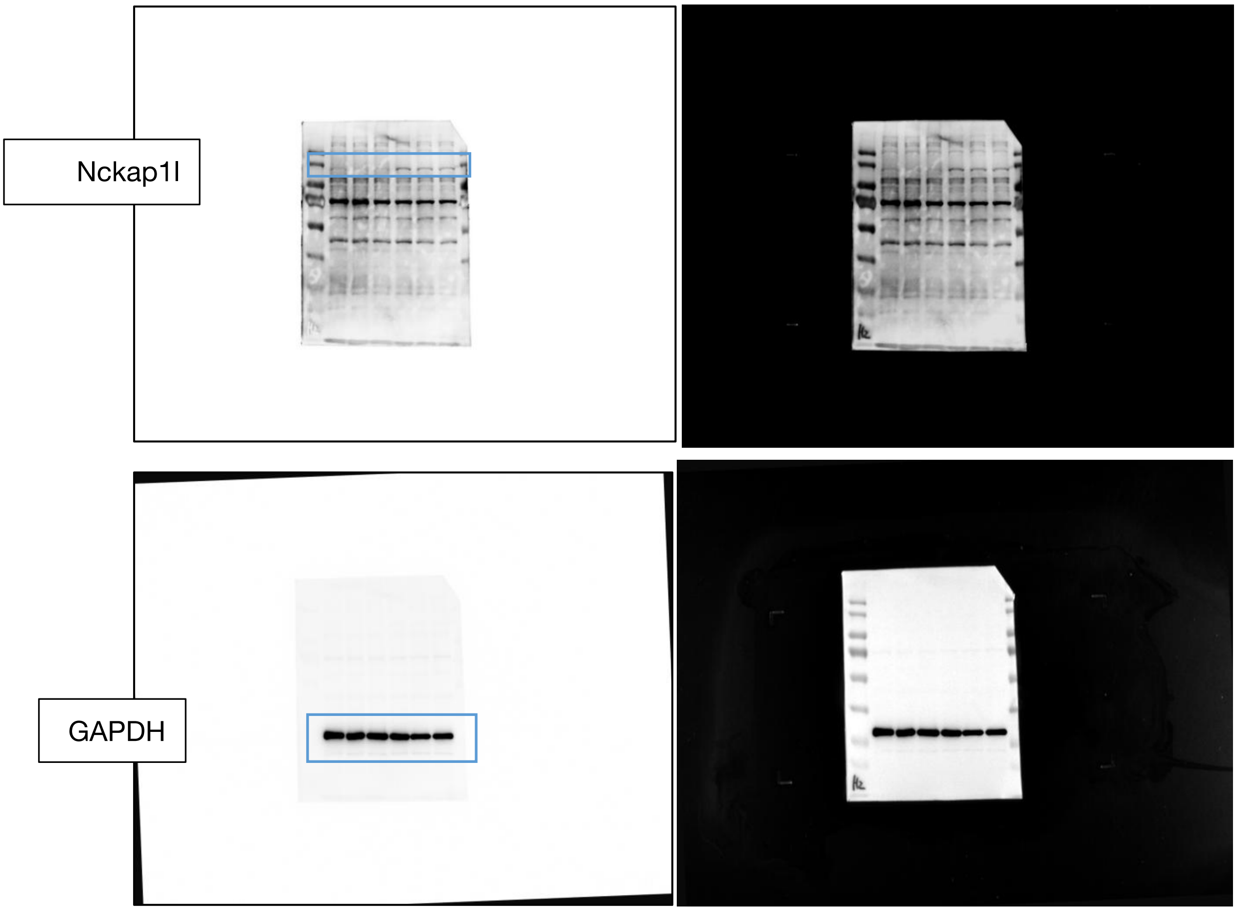

Supplementary Figure 6a

The MARKER from top to bottom are sequentially 180, 130, 100, 70, 55, 40, 35, 25, 15 (ThermoFisher Scientific #26616).

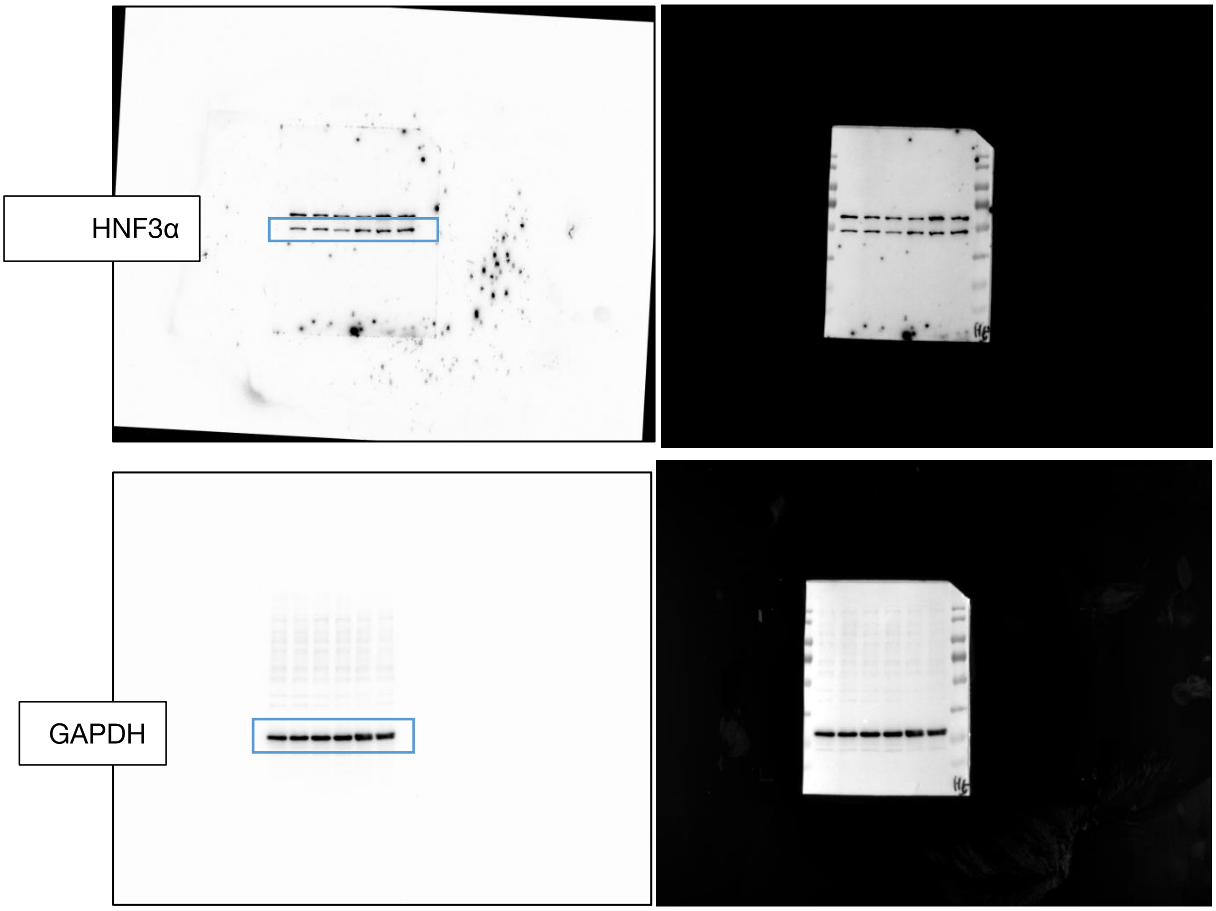

Supplementary Figure 6c

The MARKER from top to bottom are sequentially 180, 130, 100, 70, 55, 40, 35, 25, 15 (ThermoFisher Scientific #26616).

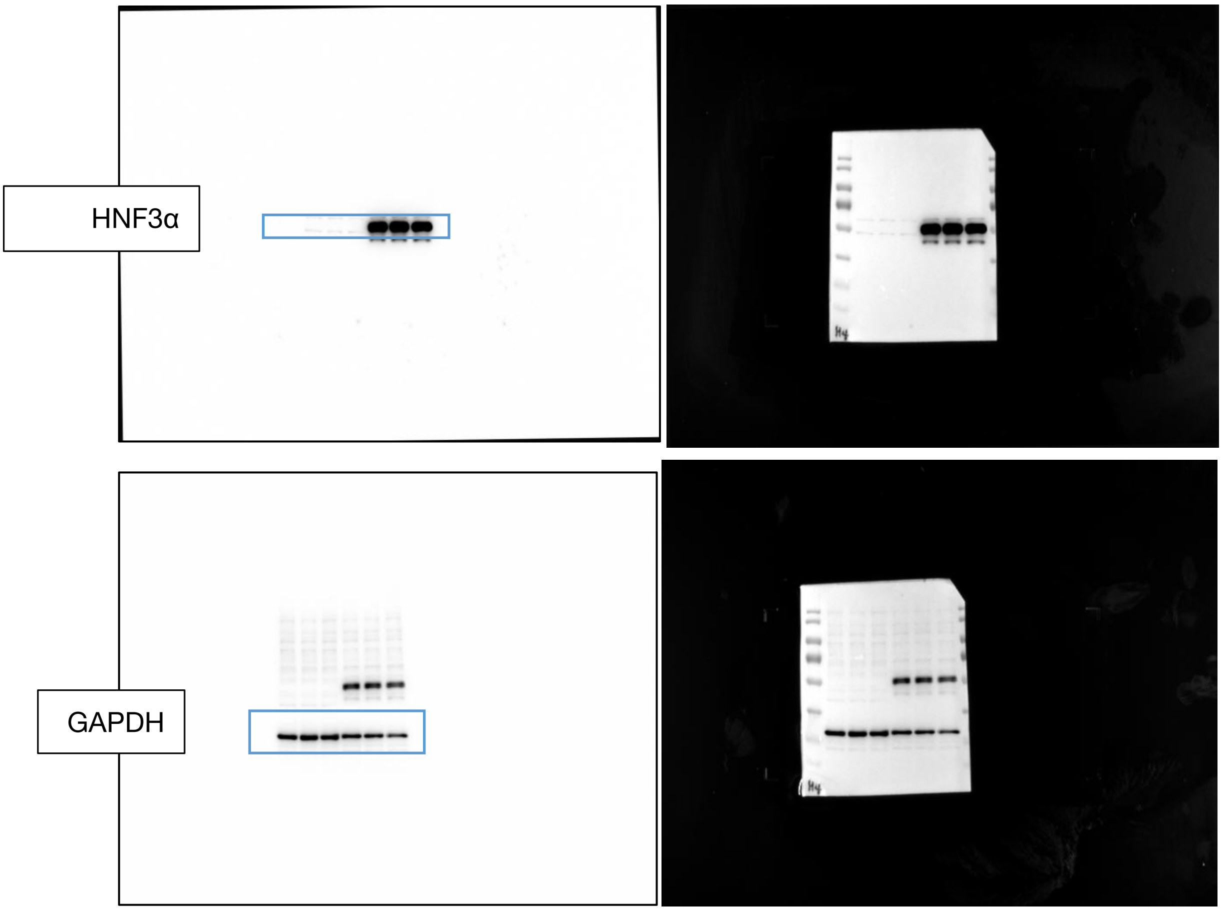

Supplementary Figure 6e

The MARKER from top to bottom are sequentially 180, 130, 100, 70, 55, 40, 35, 25, 15 (ThermoFisher Scientific #26616).

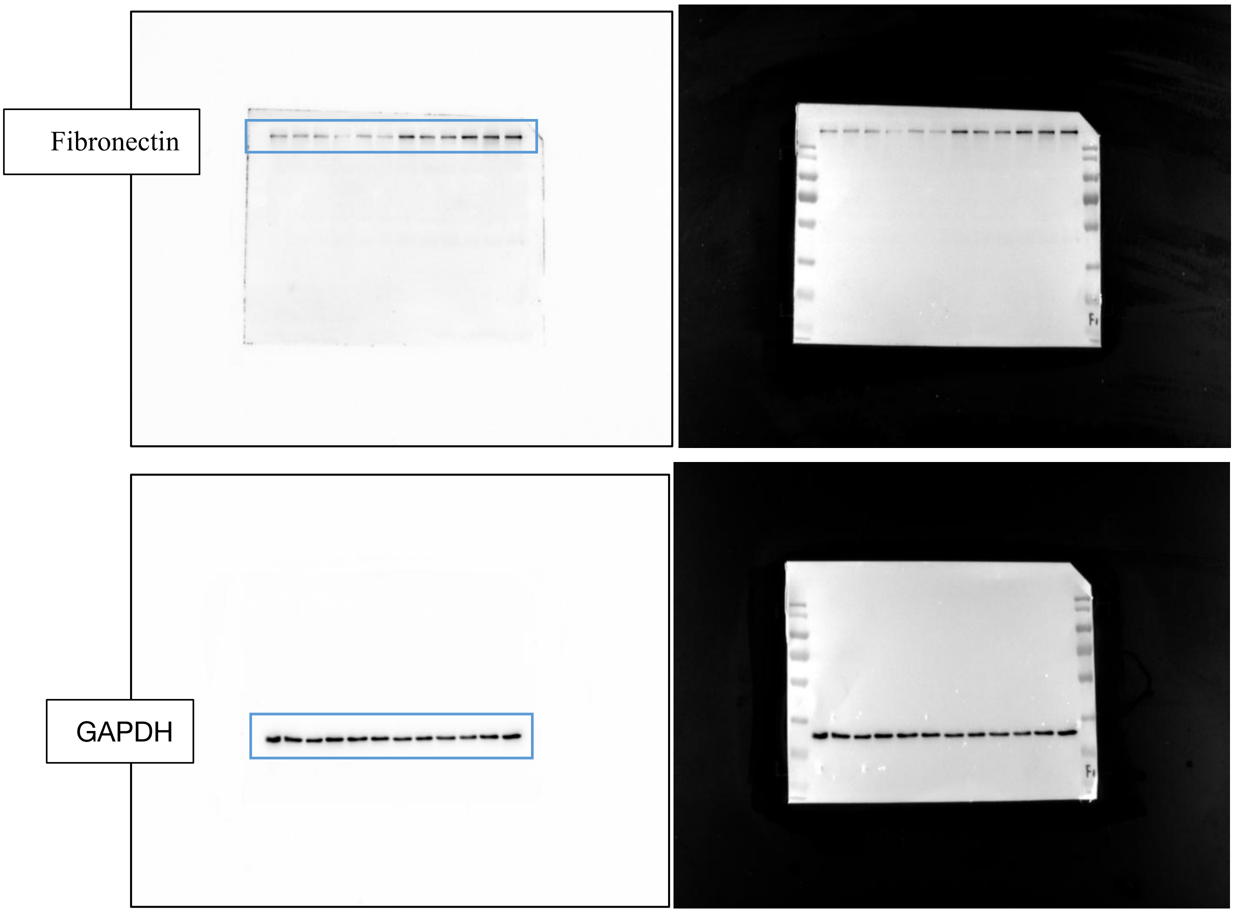

Supplementary Figure 6g

The MARKER from top to bottom are sequentially 180, 130, 100, 70, 55, 40, 35, 25, 15

(ThermoFisher Scientific #26616).

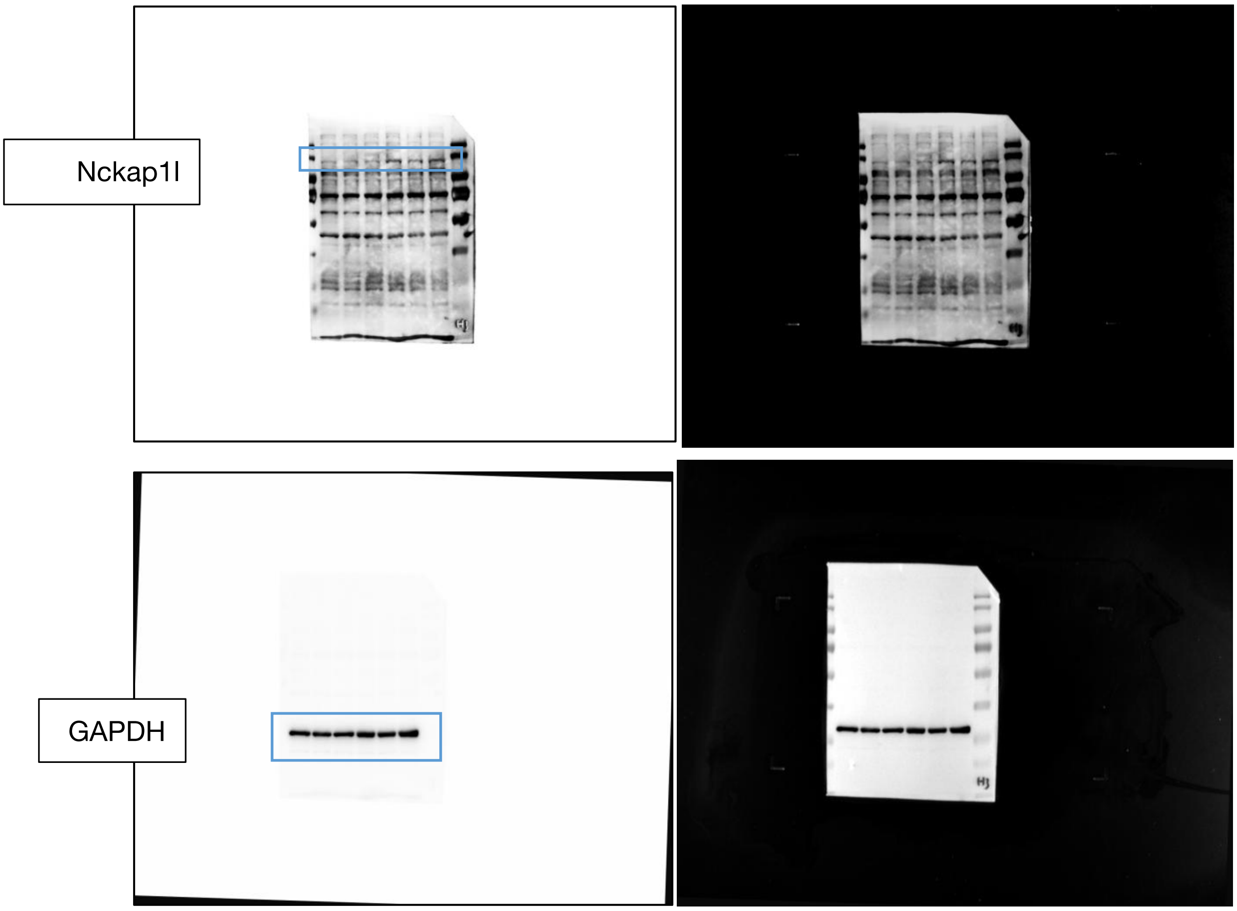

Supplementary Figure 7b

The MARKER from top to bottom are sequentially 180, 130, 100, 70, 55, 40, 35, 25, 15 (ThermoFisher Scientific #26616).

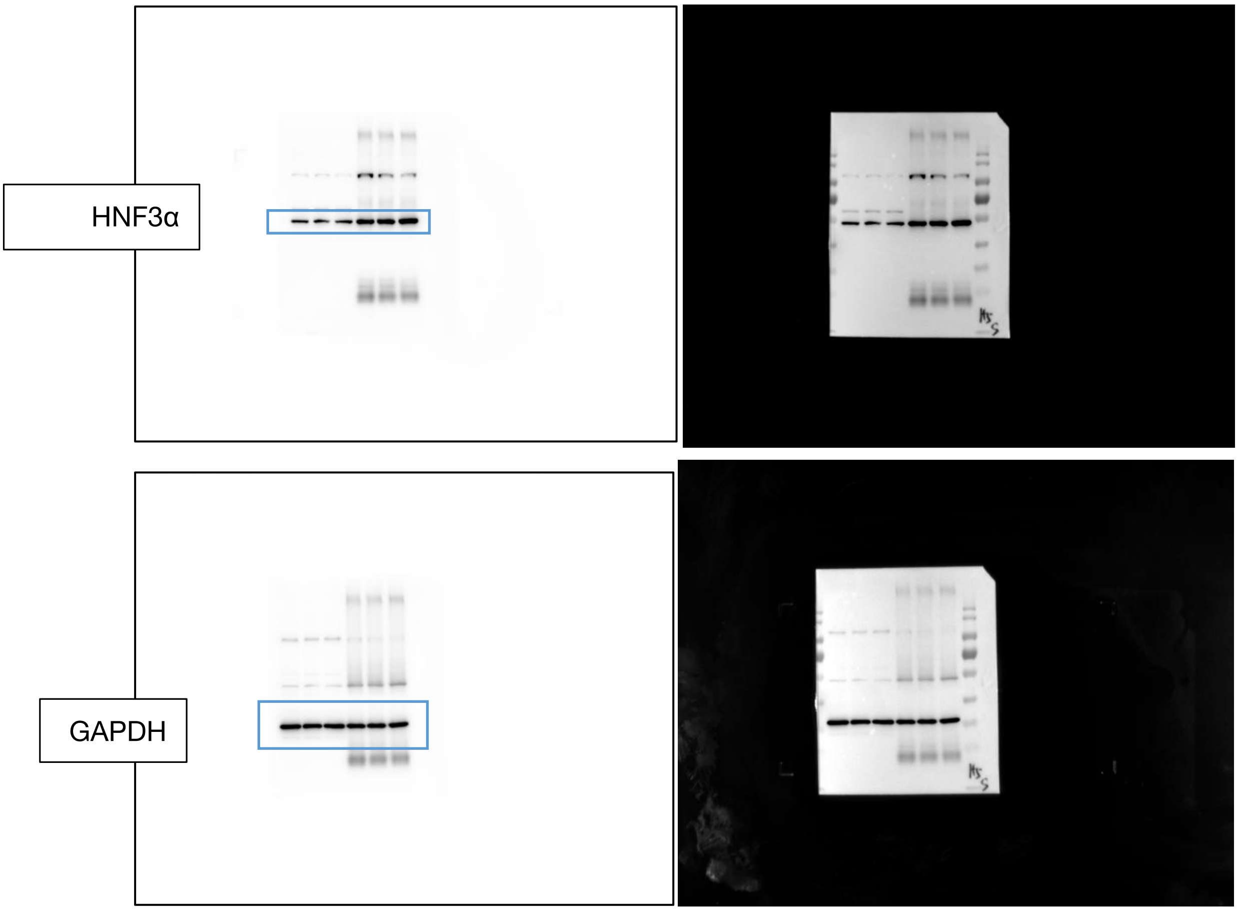

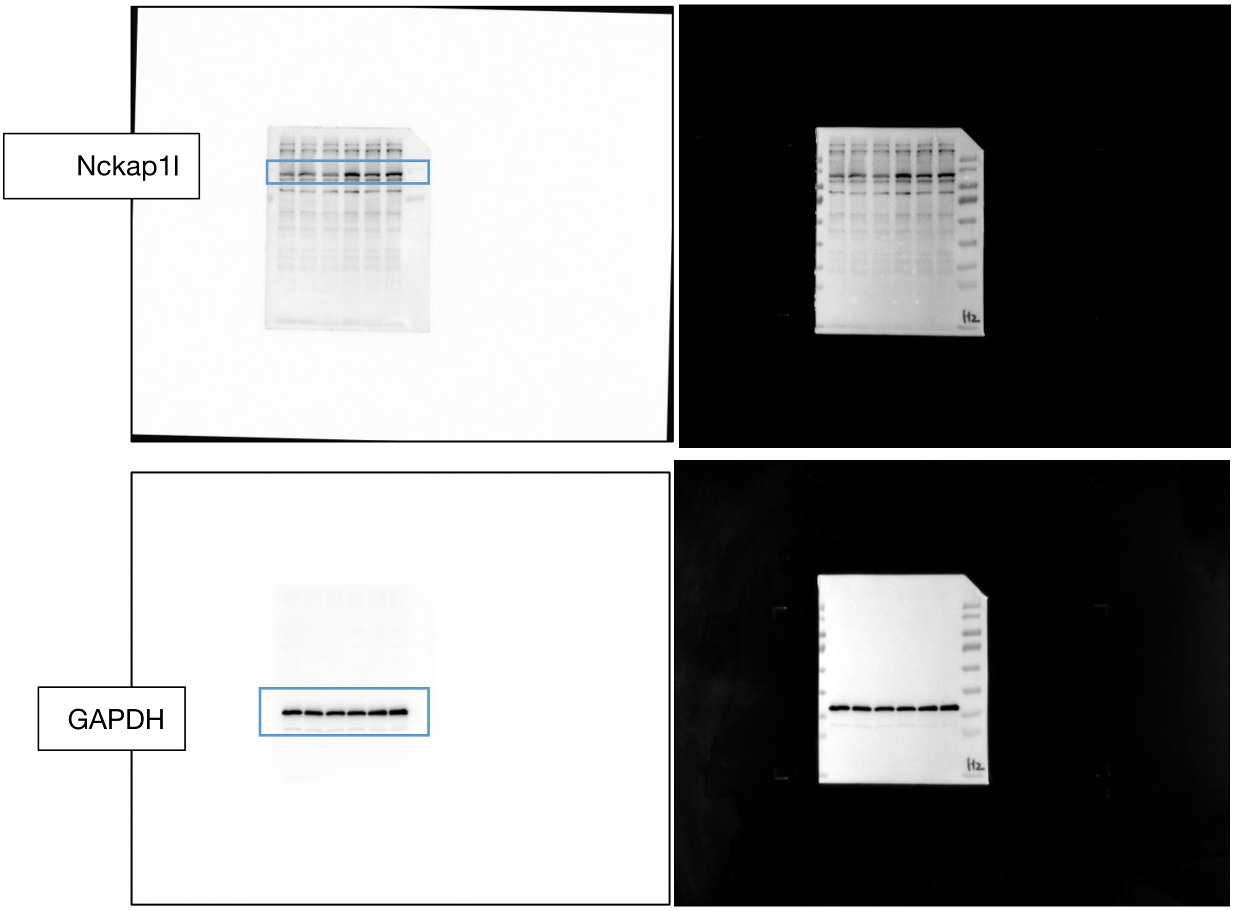
Nckap1l
GAPDH
